# CO_2_/N_2_ Separation on Highly Selective Carbon Nanofibers Investigated by Dynamic Gas Adsorption

**DOI:** 10.1002/cssc.202200761

**Published:** 2022-05-24

**Authors:** Victor Selmert, Ansgar Kretzschmar, Henning Weinrich, Hermann Tempel, Hans Kungl, Rüdiger‐A. Eichel

**Affiliations:** ^1^ Institute of Energy and Climate Research – Fundamental Electrochemistry (IEK-9) Forschungszentrum Jülich GmbH 52425 Jülich Germany; ^2^ Institute of Physical Chemistry RWTH Aachen University 52056 Aachen Germany

**Keywords:** adsorption, carbon, carbon dioxide capture, CO_2_/N_2_ selectivity, gas separation

## Abstract

The development of highly selective adsorbents for CO_2_ is a key part to advance separation by adsorption as a viable technique for CO_2_ capture. In this work, polyacrylonitrile (PAN) based carbon nanofibers (CNFs) were investigated for their CO_2_ separation capabilities using dynamic gas adsorption. The CNFs were prepared by electrospinning and subsequent carbonization at various temperatures ranging from 600 to 1000 °C. A thorough investigation of the CO_2_/N_2_ selectivity resulted in measured values of 53–106 at 1 bar and 25 °C on CNFs carbonized at 600, 700, or 800 °C. Moreover, the selectivity increased with lower measurement temperatures and lower CO_2_ partial pressures, reaching values up to 194. Further analysis revealed high long‐term stability with no degradation over 300 cycles and fast adsorption kinetics for CNFs carbonized at 600 or 700 °C. These excellent properties make PAN‐based CNFs carbonized at 600 or 700 °C promising candidates for the capture of CO_2_.

## Graphical abstract

**CO_2_ separation**: Dynamic gas adsorption makes it possible to study the adsorption of gas mixtures and analyze the separation of gases under conditions close to application. With this method, polyacrylonitrile‐based carbon nanofibers are assessed as adsorbent for carbon capture. Unlike other carbon materials, they exhibit an excellent CO_2_/N_2_ selectivity while featuring high stability as well as a simple and scalable synthesis procedure.

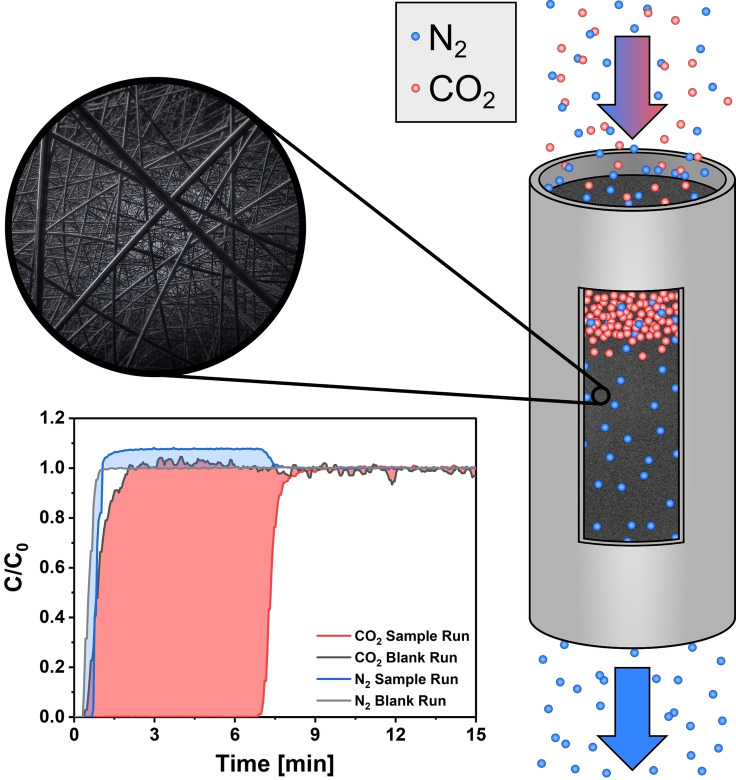

## Introduction

The emission of CO_2_ as greenhouse gas from fossil resources is one of the main contributions to the anthropogenic climate change.^[1]^ To reduce the amount of emitted CO_2_ and to limit the effect of global warming, several concepts like carbon capture and storage (CCS)^[2]^ as well as carbon capture and utilization (CCU)^[3]^ have been proposed. However, as both efficient storage and most of the investigated CCU technologies demand pure CO_2_,^[4]^ it is mandatory to effectively separate CO_2_ from other gases first. The latter can include CO_2_ separation from either flue gas or ambient air (direct air capture, DAC). However, in comparison to air (>400 ppm),^[5]^ the CO_2_ content in typical flue gases (3–15 % CO_2_)^[6]^ is significantly higher. Consequently, CO_2_ separation from flue gas seems more practical.

Currently, amine scrubbing is the only process applied on an industrial scale to remove CO_2_ from various gas streams.^[7]^ However, this technology has a number of drawbacks. In fact, the working capacity is quite low, the regeneration is very energy demanding, and degradation of the amine solution is an issue.^[7]^

Among other technologies, the separation of CO_2_ from flue gas by adsorption processes is considered as an interesting alternative.[^8^, ^9^] Porous adsorbents feature high working capacities and superior stability. Moreover, the CO_2_ is only weakly bound via physi‐ rather than chemisorption, leading to a reduced energy demand for sorbent regeneration as compared to amine scrubbing.^[10]^ The regeneration process can either be performed by a change in pressure (pressure swing adsorption, PSA) or temperature (temperature swing adsorption, TSA). Variations of these procedures such as vacuum swing adsorption (VSA),^[10]^ pressure vacuum swing adsorption (PVSA),^[11]^ and electrical swing adsorption (ESA)^[12]^ are known as well.

For an efficient process the sorbent for CO_2_ separation should feature a high working capacity, fast sorption kinetics and mass transfer, an energy‐efficient regeneration procedure, and low production costs.^[13]^ Particularly, the selectivity for CO_2_ is a crucial parameter as it has a significant impact on the process costs.^[14]^ At this, among others, the CO_2_/N_2_ selectivity is most important as N_2_ is the main component in air and in flue gas.

Until now, no sorbent was able to sufficiently incorporate all the material properties mentioned above. The adsorbents that are commonly investigated for CO_2_ separation like zeolites,^[15]^ metal‐organic frameworks (MOF),^[11]^ porous carbons,^[16]^ porous polymers,^[17]^ and hybrid materials^[18]^ have different strengths and weaknesses. Zeolites, for example, can achieve high selectivity due to size exclusion effects. However, the regeneration of zeolites is energy‐intensive, and they are sensitive to moisture.^[19]^ Carbons on the other hand are usually easy to regenerate, insensitive to gas impurities and moisture, and feature low costs.^[16]^ However, their selectivity is usually comparatively low and requires improvements.

Most recent reports regarding modified carbon materials that exhibit high CO_2_/N_2_ selectivity as predicted by calculations based on the ideal adsorbed solution theory (IAST) are as follows: Abdelmoaty et al. showed that porous carbon synthesized from a mixture of pyrazole and KOH may feature an IAST selectivity of up to 128 (10 % CO_2_, 298 K).^[20]^ To et al. reported an IAST value of 139 (10 % CO_2_, 323 K) for hierarchical N‐doped carbon based on a polypyrrole precursor.^[21]^ KOH‐activated polyacrylonitrile (PAN)‐based carbons were found to exhibit an IAST selectivity of 79 (15 % CO_2_, 323 K).^[22]^

In a recent study of ours, it was shown that non‐modified electrospun PAN‐based carbon nanofibers (CNFs) may exhibit a CO_2_/N_2_‐IAST selectivity of up to 350 (10 % CO_2_, 273 K).^[23]^ Also, it was shown that the adsorption properties of the CNFs can be tailored by the carbonization temperature. Further studies on this material focused on the sorption kinetics^[24]^ and the structural changes of the fibers during carbonization.^[25]^

As described above, with respect to the CO_2_/N_2_ selectivity, often only IAST values are reported. This simple method calculates the adsorption data of gas mixtures from the corresponding and often readily available pure‐component adsorption isotherms fitted with a suitable isotherm model.[^26^, ^27^] Although IAST calculations are rather simple, it is known that the IAST can describe various binary mixtures sufficiently well.^[27]^ However, this requires the isotherm model to match the isotherm precisely, especially at low pressures, and does not allow any violation of the assumptions made, for example the ideal adsorbed phase.^[27]^ But especially for materials with a high selectivity, the assumption of an ideal adsorbed phase might be violated as one component is significantly stronger adsorbed than the other, because of which the predicted values may deviate from the actual values.^[27]^

Therefore, measured selectivity values are more reliable and can be obtained by dynamic gas adsorption. For this technique, the gas mixture is directly applied on the adsorbent by streaming a constant gas flow over the adsorbent in a fixed bed column. From the breakthrough curve of each adsorptive the adsorbed amount, the selectivity and various other parameters can be deduced, highlighting the advantages of this method.[^13^, ^28^, ^29^] Moreover, the flow conditions at which the data is obtained are closer to the conditions in real applications like PSA or TSA processes than for static experiments.^[28]^ Further information on this technique is available in the literature.[^13^, ^28^, ^29^, ^30^, ^31^]

With this work, we seek to elucidate the agreement of the IAST results previously reported for PAN‐based CNFs^[23]^ and the experimental selectivity values under flow conditions, using dynamic gas adsorption with CO_2_/N_2_ gas mixtures. For this purpose, the selectivity at 0 °C was measured and compared to the IAST calculations. To investigate the separation capabilities of the material at conditions more typical for flue gas, the selectivity was thoroughly measured at additional temperatures as well as different pressures and gas compositions. In addition, the breakthrough and desorption curves are analyzed with regard to the sorption kinetics since fast mass transfer and adsorption kinetics are vital for an efficient separation process. For a comparison with N_2_ and CO_2_ isotherms obtained by static gas adsorption, N_2_ and CO_2_ isotherms were acquired by dynamic adsorption as well. Additionally, the isotherm data were complemented by high‐pressure isotherms up to a partial pressure of 18 bar.

## Results and Discussion

### Sorption isotherms

**Figure 1:**
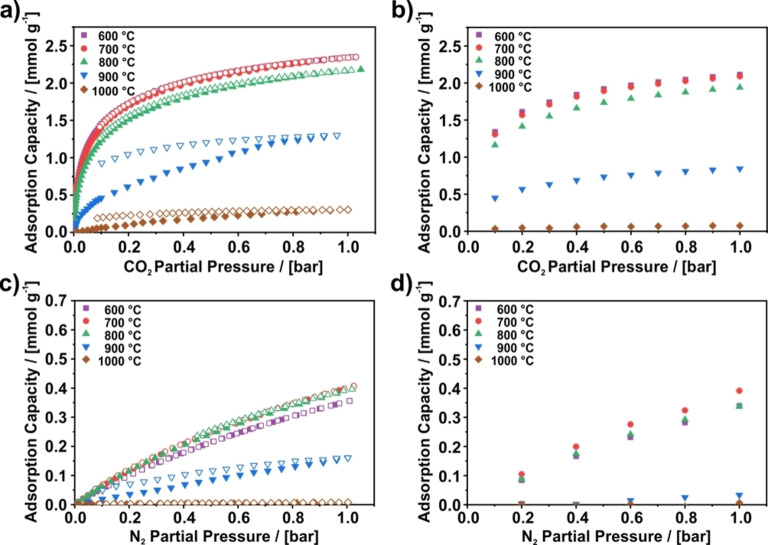
Isotherms of (a,b) CO_2_ and (c,d) N_2_ measured at 273 K on PAN‐based carbon nanofibers carbonized at various temperatures ranging from 600 to 1000 °C. (a,c) Results for the static sorption method. Closed symbols represent the adsorption, open symbols describe the desorption. (b,d) Results for the dynamic sorption method. A direct comparison of the static and dynamic isotherms can be found in Figures S1 and S2. The corresponding breakthrough curves are shown in Figures S15 and S16.

To study the CO_2_/N_2_ separation potential of PAN‐based carbon nanofibers (CNFs) carbonized at temperatures ranging from 600 to 1000 °C, at first, pure component sorption isotherms of CO_2_ and N_2_ are discussed. The sorption isotherms of CO_2_ and N_2_ are shown in Figure 1a–d, respectively. For both gases, the isotherms have been acquired applying static and dynamic gas adsorption to obtain both data in equilibrium and under dynamic flow conditions with the latter being closer to application in a PSA device.

Briefly summarizing the results of the static CO_2_ adsorption, the CNFs carbonized in a temperature range from 600 to 800 °C exhibit concave isotherms with a high CO_2_ adsorption capacity of up to 2.3 mmol g^−1^ adsorbed CO_2_ at 1.0 bar. The CO_2_ uptake is already high at low pressures, which is attributed to a large amount of ultramicropores.^[23]^ In contrast, the CO_2_ adsorption capacity of CNFs carbonized at 900 and 1000 °C is drastically reduced, which was explained previously by a size reduction of the ultramicropores due to an increasing carbonization temperature. As a consequence, the ultramicropores become partially inaccessible for CO_2_ at 900 °C and completely inaccessible at 1000 °C. A correlation with the amount of surface functional groups was not apparent.^[23]^

In Figure 1b the CO_2_ isotherms acquired using the dynamic gas adsorption technique are shown. Again, the samples carbonized at 600, 700, and 800 °C exhibit concave isotherms with a high CO_2_ adsorption capacity of up to 2.1 mmol g^−1^ at 1 bar. In contrast, the sample carbonized at 900 °C exhibits a significantly lower CO_2_ adsorption capacity of 0.84 mmol g^−1^ at 1 bar, and the sample carbonized at 1000 °C yields a linear isotherm with an even lower CO_2_ adsorption capacity of 0.07 mmol g^−1^ at 1 bar.

A comparison of the static and dynamic CO_2_ isotherms in Figure 1a,b shows that the CO_2_ adsorption capacity of the samples carbonized at 800 °C or below is in good agreement with each other as the deviations between both methods amount to less than 10 % and are within experimental deviation. However, CNFs prepared at higher carbonization temperature exhibit a reduced adsorption capacity under dynamic flow conditions. For the sample carbonized at 900 °C, the deviation in capacity between the dynamic and static measurement equals 35 % at 1.0 bar and increases for the sample carbonized at 1000 °C to 75 %. A deviation from the statically measured loading usually indicates slow adsorption kinetics or diffusion, because the equilibrium is not attained within the breakthrough time.^[13]^ As the deviation becomes apparent for samples that were carbonized at 900 and 1000 as compared to 800 °C, it is obvious that the adsorption kinetics slow down for an increased carbonization temperature. Additionally, this finding is supported by the strong pseudo‐irreversibility in Figure 1a observed for these two samples.

Summarizing the results for the static N_2_ adsorption in Figure 1c, all the isotherms exhibit a similar, almost linear shape and a low adsorption capacity. However, while the N_2_ adsorption capacities at 1 bar of samples carbonized below 900 °C reach about 0.35 to 0.40 mmol g^−1^, the adsorption capacity is only 0.16 and 0.008 mmol g^−1^ for the CNFs carbonized at 900 and 1000 °C, respectively.

Similar to the CO_2_ adsorption, the reduction of the ultramicropore size might explain the significant drop of the N_2_ adsorption capacity for CNFs carbonized at 900 and 1000 °C as compared to the CNFs carbonized at 800 °C, since CO_2_ and N_2_ are of similar size according to Breck^[32]^ (0.33 vs. 0.364 nm) or Webster et al.^[33]^ (0.319 vs. 0.299 nm). However, due to the lower adsorption capacity for N_2_ the affinity of the fibers towards N_2_ appears to be much lower than towards CO_2_, which might stem from the reduced polarizability and quadrupole moment of N_2_ compared to CO_2_ ^[34]^ and the lower condensability of N_2_ compared to CO_2_ as their evaporation temperatures deviate significantly (77.35^[35]^ vs. 194.65 K^[36]^).

Figure 1d depicts N_2_ isotherms that were recorded using the dynamic gas adsorption technique. For this technique, the linear isotherms of the CNFs carbonized at 600 to 800 °C exhibit a N_2_ adsorption capacity that is about 0.3 mmol g^−1^ higher at 1 bar than the N_2_ adsorption capacity of the samples carbonized at higher temperatures. A comparison of the static and dynamic N_2_ isotherms in Figure 1c,d reveals mostly marginal differences between both methods. Only the sample prepared at 900 °C exhibits a significantly lower adsorption capacity under dynamic flow conditions. As discussed for CO_2_, this can be attributed to kinetic limitations present on this sample, which is supported by the pseudo‐irreversibility of N_2_ isotherm (Figure 1c).

Separate plots comparing the static and dynamic isotherms for each sample can be found in Figures S1 and S2 in the Supporting Information.

### High‐pressure isotherms

**Figure 2:**
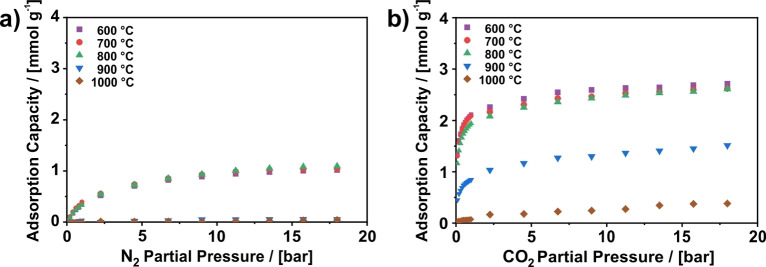
High‐pressure adsorption isotherms of (a) N_2_ and (b) CO_2_ measured at 273 K on PAN‐based CNFs carbonized at various temperatures ranging from 600 to 1000 °C using the dynamic sorption method. The dynamic isotherms from Figure 1b,d up to 1 bar are included again for direct comparison. The corresponding breakthrough curves are shown in Figures S17 and S18.

For separation processes like PSA, the adsorption properties in pressure ranges beyond standard sorption isotherms (<1 bar) are relevant as well. Therefore, high‐pressure adsorption isotherms up to a partial pressure of 18 bar were recorded using dynamic gas adsorption. Figure 2a depicts the isotherms for N_2_ adsorption. Here, even under high pressure, the CNFs prepared at 900 and 1000 °C still do not adsorb significant amounts of N_2_. In contrast, the samples carbonized at 600, 700, and 800 °C exhibit an increase in N_2_ uptake from 0.39 mmol g^−1^ at 1 bar to 1.0 mmol g^−1^ at 18 bar. However, the linear shape of the isotherms in the range from 0.1 to 1 bar is not retained at higher pressures as the slope of the isotherms flattens and becomes almost horizontal at 18 bar. A similar effect is visible for the CO_2_ isotherms in Figure 2b, where the slopes of the high‐pressure isotherms flatten as well with increasing pressure. However, for CO_2,_ this is already observable at pressures below 1 bar (Figure 1a). This observation can be attributed to the pore structure of the CNFs.^[23]^ The majority of pores present on this material are ultramicropores. After filling of these pores, further adsorption can only take place on the outer surface of the fibers, which have a relatively low Brunauer‐Emmett‐Teller (BET) area (measured with Ar) of 13 to 16 m^2^ g^−1^ and, therefore, result in a low additional uptake of CO_2_ or N_2_.^[23]^

For the application in a PSA process this means increasing the partial pressure of CO_2_ above 1 bar has nearly no beneficial influence on the working capacity. Thus, depending on the CO_2_ content in the gas mixture, this material might be most interesting for VSA or VPSA applications.

### Kinetics

In this section, breakthrough curves are analyzed to obtain insight into the sorption kinetics of CO_2_ on the CNFs, since the shape of a breakthrough contains information about the adsorption and diffusion processes taking place in a fixed bed column.^[31]^ To do so, breakthrough curves were measured with 3 % CO_2_ in He at 0 °C and 5 bar. The shape of the breakthrough curves and their corresponding desorption curves are discussed qualitatively. For a quantitative assessment, the breakthrough curves were modeled with the linear driving force approach using the commercial software 3PSim.^[37]^ In this approach, the sorption rate is considered to be proportional to the difference between the equilibrium loading and the current loading. The proportionality constant is the mass transfer coefficient *k*_LDF_ that is a measure for the rate of adsorption on the corresponding material. To obtain the mass transfer coefficient *k*_LDF_ for each sample, the modeled breakthrough curve was fitted to the experimental breakthrough curve by varying *k*_LDF_. For the modeling the following assumptions are made:

    

- Heat effects are neglected (isothermal conditions).
- The gas phase obeys the ideal gas law.
- There is no pressure drop along the column.
- The flow behavior can be described as a dispersed plug flow.
- The radial dispersion is negligible.
- The column is uniformly packed.

   

A description of the applied formula is given in the Supporting Information. In addition, Table 1 provides a list of the applied parameters. For a mathematical description of the isotherm data, needed for the implementation into the model, the dynamic isotherms in Figure 1b were fitted with Tóth's isotherm model (Table S1).

**Table 1:** Parameters for the modeling of the breakthrough curves in Figure 3.

Carbonization temperature [ °C]	Column height^[a]^ [cm]	Column diameter [cm]	Sample mass [g]	Particle diameter [mm]	Bed porosity	Particle porosity	Axial dispersion [cm^2^ min^−1^]
600	8.0	0.8	1.29	1.0	0.63	0.5	9.68
700	9.5	0.8	1.39	1.0	0.67	0.5	9.09
800	9.5	0.8	1.41	1.0	0.67	0.5	9.08
900	10.0	0.8	1.44	1.0	0.67	0.5	9.15

[a] The column height in the simulation is adjusted to the filling height of the corresponding breakthrough experiment.

**Figure 3:**
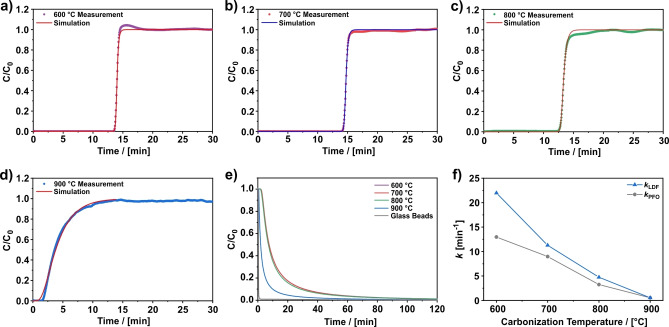
(a–d) Measured and modeled breakthrough curves of CO_2_. (e) Corresponding desorption curves. (f) Mass transfer coefficients resulting from the modeled breakthrough curves (*k*_LDF_) shown in (a–d) in comparison to results from static gas adsorption experiments (*k*_PFO_).^[24]^ The breakthrough curves were measured on PAN‐based CNFs carbonized at various temperatures ranging from 600 to 900 °C with 3 % CO_2_ in Helium at 5 bar, 0 °C, and a flow rate of 100 mL min^−1^. The desorption curves were measured with 100 % He at 5 bar, 273 K, and 97 mL min^−1^. Plotted for each curve is the normalized concentration *C*/*C*_0_, where *C* denotes the concentration at the column outlet and *C*_0_ the concentration at the column inlet. The parameters of the simulation are given in Table 1.

Measured breakthrough curves for samples carbonized in a temperature range from 600 to 900 °C are shown in Figure 3a–d in comparison to the corresponding modeled breakthrough curve as normalized concentration *C*/*C*_0_ versus time plots. For all samples a good agreement of the model with the experimental shape of the breakthrough curve is observed. A qualitative comparison of the shape between the individual samples reveals a clear difference. For CNFs carbonized at 600, 700, and 800 °C steep and focused breakthrough curves are found, whereas a further increase of the carbonization temperature to 900 °C results in a disperse and asymmetric curve as shown in Figure 3d. The latter is an unexpected observation because, in general, the shape of a breakthrough curve is primarily defined by thermodynamics.^[28]^ A concave isotherm should result in a sharp and focused breakthrough curve, whereas a convex isotherm will broaden the concentration front.^[38]^ As the CO_2_ isotherms of the CNFs carbonized between 600 and 900 °C all have concave isotherms (Figure 1), steep and focused breakthrough curves for all samples are expected. However, the thermodynamically favored shape is only obtained for the CNFs carbonized up to 800 °C, whereas the CNFs prepared at 900 °C exhibit a disperse curve. Besides the isotherm shape, various other factors like adsorption kinetics, axial dispersion, and temperature changes can affect the shape as well.[^28^, ^38^, ^39^] Thus, a possible explanation for the broad and asymmetric breakthrough curve can be slow adsorption kinetics or an increase in the experimental temperature (here: set to 0 °C) due to the release of the heat of adsorption, which is a known effect in literature described as tailing.^[40]^ However, as the amount of sample used in the present study is small and the rise in temperature is only about 0.4 °C, the actual measurement conditions can be regarded as isothermal and the tailing effect can be precluded. Thus, the flattening of the breakthrough curve can be attributed to kinetic limitations.

The CO_2_ desorption is investigated as well, and the results are shown in Figure 3e. In contrast to the adsorption behavior, all samples exhibit disperse desorption curves. Furthermore, the desorption curves of the samples carbonized at 600, 700, and 800 °C are almost identical, whereas the desorption curve of the CNFs prepared at 900 °C distinguishes itself not by its shape but its position. Due to the smaller CO_2_ adsorption capacity compared to the other samples, less CO_2_ desorbs, and thus, the desorption curve appears earlier. The shape of all desorption curves can be attributed to thermodynamics. In contrast to the adsorption, thermodynamics of a concave isotherm cause a disperse concentration front during the desorption,^[38]^ prolonging the desorption process and masking the possible differences in adsorption rate between the samples. As a consequence, longer desorption times prolong the regeneration period, which is potentially disadvantageous for practical application since the adsorption and regeneration in swing processes are typically performed in parallel.[^28^, ^41^]

The mass transfer coefficient *k*_LDF_ obtained from the modeling approach is a measure for the adsorption rate on a sample and is shown in Figure 3f. With increasing carbonization temperature *k*_LDF_ declines. In the range of 600 to 800 °C, *k*_LDF_ roughly halves for every 100 °C in temperature increase. A more significant drop is observable from 800 (4.75 min^−1^) to 900 °C (0.55 min^−1^) as the mass transfer coefficient decreases almost one order of magnitude, highlighting the strong kinetic limitations of this sample and resulting in the disperse breakthrough curve in Figure 3d. These strong kinetic limitations have been discussed for this sample in the isotherms section, where deviations of the adsorption capacity between the dynamic and static measured CO_2_ isotherm in Figure 1a,b are apparent. An additional investigation on the influence of different flowrates on the adsorption capacity can be found in Figures S3 and S4 in the Supporting Information. It was found that the sample prepared at 900 °C exhibits a flowrate‐dependent adsorption capacity with higher CO_2_ uptake at lower flowrates, illustrating the slow kinetics of this sample.

In our previous work, the equilibration curves of static CO_2_ adsorption measurements were fitted using a pseudo‐first‐order (PFO) kinetic model that is similar to the linear driving force approach in this work. The mass transfer coefficient *k*_PFO_ obtained from that work (10 mbar CO_2_ partial pressure) is shown in Figure 3f in comparison to *k*_LDF_ from this work (15 mbar CO_2_ partial pressure). As observed for *k*_LDF_, *k*_PFO_ decreases with increasing carbonization temperature and exhibits a significant decline from 800 (3.24 min^−1^) to 900 °C (0.542 min^−1^) by six times. In addition, the values of both methods match each other reasonably well considering the different experimental origins, thus yielding a good agreement of both approaches.

All in all, the findings are consistent with the pore model for the investigated CNFs.^[23]^ The slowdown of the sorption kinetics with increasing carbonization temperature is attributed to a decrease in pore size. Thus, smaller pores limit the diffusion at some point, leading to a reduced adsorption rate.

### Cycle stability

**Figure 4:**
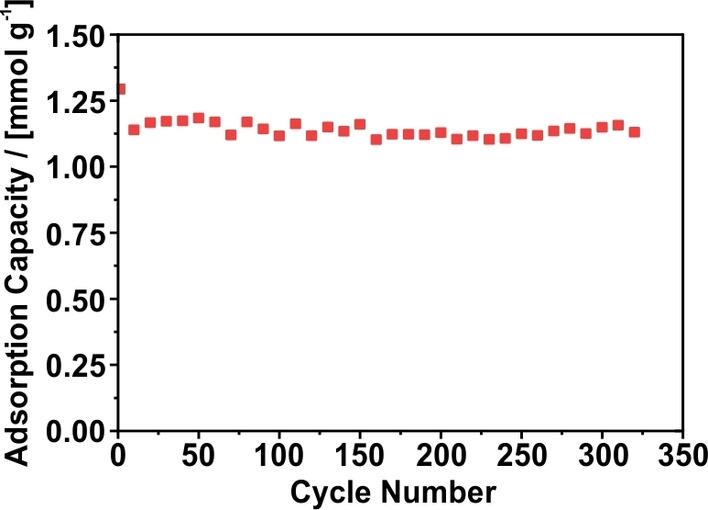
CO_2_ adsorption capacity of PAN‐based CNFs carbonized at 700 °C measured over more than 300 adsorption and desorption cycles with the dynamic sorption method. Adsorption was performed with 3 % CO_2_ in N_2_ at 10 bar and 25 °C, whereas desorption was performed with 100 % N_2_ at 1 bar and 25 °C.

Beyond fundamental investigations, the long‐term stability of the analyzed CNFs was investigated, too (Figure 4). For the long‐term testing, a CNF sample carbonized at 700 °C was chosen, given its superior adsorption capacity for CO_2_ as compared to most of the other samples. During the long‐term testing more than 300 cycles of CO_2_ adsorption and desorption were performed, which is shown in Figure 4. The adsorption was performed with 3 % CO_2_ in N_2_ at 25 °C and 10 bar, whereas the desorption was carried out at 1.0 bar with 100 % N_2_. As a result of this investigation, it can be seen that the adsorption capacity is largely stable and as high as 1.14±0.03 mmol g^−1^ during almost the whole experiment. Only the first cycle exhibits a slightly higher adsorption capacity (1.29 mmol g^−1^), prior to which the sample was degassed at 250 °C, indicating that 0.15 mmol g^−1^ of CO_2_ do not desorb during the following regeneration steps with N_2_ at 25 °C.

All in all, the stable adsorption capacity over a long period indicates a high stability of the material itself, which is a significant advantage of carbon materials reported in literature.[^9^, ^42^] However, most studies provide only five to ten adsorption/desorption cycles, whereas this study covers a considerably larger time scale.

### CO_2_/N_2_ selectivity

**Figure 5:**
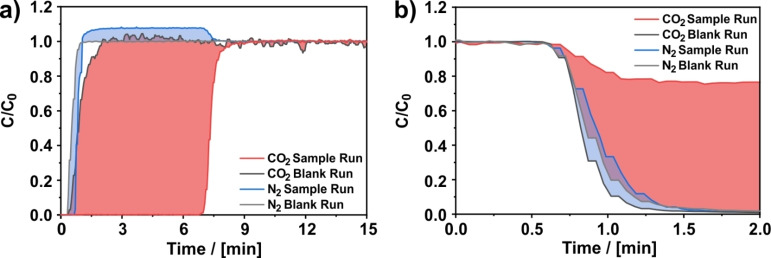
(a) Breakthrough curves and (b) desorption curves of the selectivity measurement. Adsorption was performed with 50 % He, 45 % N_2_, and 5 % CO_2_ at 273 K and 5 bar on PAN‐based CNFs carbonized at 700 °C and is shown in comparison to a blank run. The corresponding desorption curves were measured with 100 % He at 5 bar and 273 K. Plotted for each curve is the normalized concentration *C*/*C*_0_, where *C* denotes the concentration at the column outlet and *C*_0_ the concentration at the column inlet.

When discussing the separation capabilities of a material, the selectivity is one of the most crucial properties.^[14]^ To obtain a detailed insight into the CO_2_/N_2_ selectivity of the investigated CNFs, breakthrough experiments with CO_2_ and N_2_ as adsorptives and helium as non‐adsorbing carrier gas were performed. The breakthrough curves are shown exemplarily in Figure 5a for an experiment performed with 5 % CO_2_ and 45 % N_2_ in helium on CNFs carbonized at 700 °C (all corresponding breakthrough curves are shown in the Supporting Information). In the diagram, the amount of adsorbed gas is proportional to the integral between the breakthrough curve of the sample and the blank run. For CO_2_, there is one large integral up to 8 min corresponding to the adsorption of CO_2_ (light red integral). For N_2_, two different integrals are apparent (light blue integral): The first integral from 0.3 to 1.0 min corresponds to the initial adsorption of N_2_, whereas the second integral from 1.0 to 7.5 min is attributed to a roll‐up effect, where the N_2_ concentration rises over the inlet N_2_ concentration. This roll‐up of N_2_ is attributed to the absence of CO_2_ at the outlet as well as the desorption of N_2_ caused by the displacement by CO_2_.^[31]^

A qualitative comparison of the integrals corresponding to the N_2_ and CO_2_ sorption in Figure 5a reveals that CO_2_ is adsorbed in significantly higher quantities than N_2_, although its partial pressure is nine times lower than the partial pressure of N_2_. This already indicates a high selectivity of the CNFs towards CO_2_. However, the high selectivity towards CO_2_ and the corresponding low N_2_ uptake can induce some challenges in order to precisely quantify the amount of the light component, here N_2_, as reported by Wilkins et al.[^31^, ^43^, ^44^] Due to the high difference in breakthrough time of CO_2_ (7.5 min) and N_2_ (1.0 min) the displaced amount of N_2_ is eluted over a relatively long period of time (6.5 min) and, therefore, results in a high inaccuracy. A more reliable method to quantify the amount of adsorbed N_2_ as proposed by Wilkins et al.[^31^, ^43^] is to measure a desorption curve with helium as illustrated in Figure 5b and quantify the amount of adsorbed N_2_ by integration of the desorption curve. From the amount of adsorbed CO_2_ obtained from the adsorption and the amount of adsorbed N_2_ quantified from the desorption curve, the selectivity *S* can be calculated according to Equation 1:

(1) $$S=\frac{q_{{CO}_{2}}\;y_{N_{2}}}{q_{N_{2}}\;y_{{CO}_{2}}}$$

**Figure 6:**
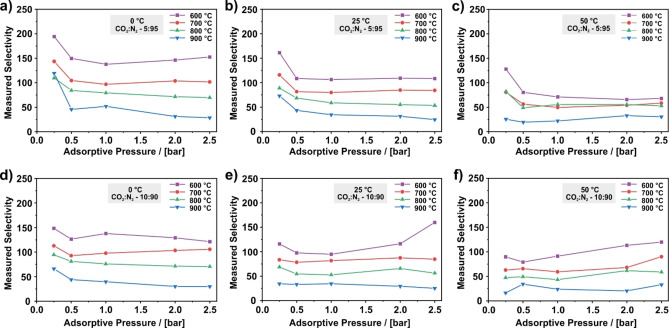
Measured CO_2_/N_2_ selectivity of PAN‐based CNFs carbonized at various temperatures ranging from 600 to 1000 °C, determined for a CO_2_/N_2_ ratio in the gas phase of (a–c) 5 : 95 and (d–f) 10 : 90. The corresponding measurements were performed at (a,d) 0, (b,e) 25, and (c,f) 50 °C. The adsorptive pressure is the sum of the CO_2_ and N_2_ partial pressure. For the measurement the overall pressure was kept constant at 5 bar and the content of N_2_ and CO_2_ was varied. The adsorbed amount of CO_2_ and N_2_ for each selectivity value is shown in Figures S5 and S6, respectively.

Here, *q_i_* denotes the adsorbed amount of component *i*, and *y_i_* denotes the volume fraction of component *i* in the gas phase. The results for the corresponding analysis are shown in Figure 6, where the selectivity is plotted against the adsorptive pressure that is equal to the sum of the CO_2_ and N_2_ partial pressure. In addition, Figure 6 shows the influence of the temperature and CO_2_/N_2_ ratio on the selectivity. The two rows in Figure 6 represent the results for different CO_2_/N_2_ ratios in the gas phase, 5 : 95 on the top (a,c,e) and 10 : 90 on the bottom (b,d,f), while each column was obtained at different temperatures, 0 °C on the left (a,b), 25 °C in the middle (c,d), and 50 °C on the right (e,f) (values for the sample carbonized at 1000 °C are not shown due to the insignificant amount of adsorbed CO_2_ and N_2_ on this sample).

A look at the results in Figure 6 reveals that independent of the conditions applied for the individual measurements, each plot exhibits the same pressure dependence: The selectivity is highest at low pressure and decreases on pressure increase. For example, in Figure 6c the selectivity of the CNFs carbonized at 700 °C changes from 116 at 0.25 bar to 82 at 0.5 bar. However, the changes from 0.5 to 2.5 bar are only minor, indicated by the curve approaching a constant value or a slightly declining slope. The same is true for the majority of the other curves in Figure 6.

At a first glance, the comparison of the feed gas composition of 5 : 95 and 10 : 90 CO_2_/N_2_ shows only insignificant differences. Curve shapes and the selectivity values resemble each other at the same conditions. However, a closer look reveals that a smaller CO_2_ fraction slightly enhances the selectivity at an adsorptive pressure of 0.25 bar as depicted in Figure S8. For example, the selectivity at 25 °C and 0.25 bar increases from around 115 at 10 % CO_2_ (Figure 6d) to 161 at 5 % CO_2_ (Figure 6c) for the sample carbonized at 600 °C. However, at an adsorptive pressure of 1 bar or higher, significant differences are not apparent.

In addition, in Figure 6 the influence of the experimental temperature is clearly apparent as the selectivity increases for a decreasing measurement temperature. For example, a reduction from 50 to 0 °C almost doubles the selectivity of the CNFs carbonized at 600 °C from 71.1 (Figure 6c) to 138 (Figure 6a) at 1.0 bar (5 : 95). This is caused by a higher dependence on the temperature for the stronger adsorbed CO_2_ than for the weaker adsorbed N_2_ (Figures S5 and S6).

**Figure 7:**
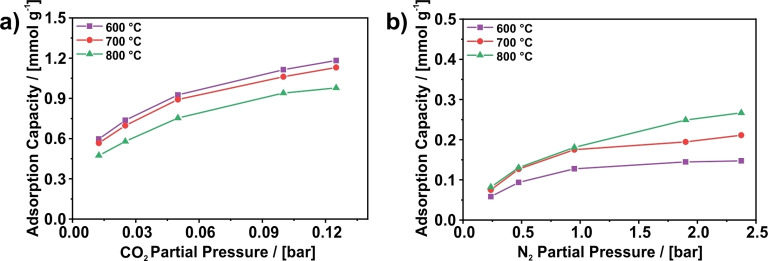
Loading of (a) CO_2_ and (b) N_2_ from a mixture of both with a CO_2_/N_2_ ratio of 5 : 95 measured with dynamic gas adsorption. The measurements were performed with helium as carrier gas at 5 bar and 0 °C. The values of CO_2_ are obtained from the breakthrough curves, whereas the values of N_2_ are obtained from the desorption curves.

The trend along the samples carbonized at various temperatures and investigated under the same conditions shows the highest selectivity values for the CNFs carbonized at 600 °C and a decline in selectivity with increasing carbonization temperature. This decline is rather continuous along the samples, which is somewhat surprising considering the similar CO_2_ and N_2_ isotherms of the samples carbonized at 600, 700, and 800 °C. A comparison of the amount of CO_2_ and N_2_ adsorbed on these samples shown in Figures 7, S5, and S6 reveals that the difference in selectivity mostly results from the different amount of adsorbed N_2_. Among these three samples, the sample prepared at 600 °C adsorbs the least N_2,_ whereas the sample prepared at 800 °C adsorbs the most N_2_. In consideration of the almost identical pure component N_2_ isotherms of these samples (Figure 1), this finding indicates that CO_2_ displaces N_2_ more efficiently, the lower the carbonization temperature is.

The high CO_2_/N_2_ selectivity of the CNFs can be attributed to the amount of ultramicropores present on the samples as described in the isotherms section. Although the isotherms indicate that N_2_ is also able to enter these ultramicropores on samples carbonized at temperatures of up to 900 °C, it was found that compared to CO_2_ significantly less N_2_ is adsorbed in these pores. However, the reason for the linear decline of the selectivity with increasing carbonization temperature is not entirely clear. The more efficient displacement of N_2_ at lower carbonization temperature might either be an effect of the different pore sizes or a result of the different surface chemistry. Previously, a continuous decrease of the amount of heteroatoms like nitrogen with increasing carbonization temperature was found.^[23]^ As nitrogen species are reported to result in a stronger CO_2_ binding due to Lewis acid‐base interactions,^[45]^ the higher nitrogen content in the CNFs prepared at 600 °C than in the samples prepared at higher temperatures might cause a stronger binding of CO_2_ and, thus, a more efficient displacement of N_2_ by CO_2_ on this sample.

The pressure dependence of the selectivity curve can be explained by the adsorption in the ultramicropores. Due to their high adsorption potential, the ultramicropores are already filled at very low pressures.^[46]^ This results in a high uptake of CO_2_ and thus, in a high selectivity at low pressures. With increasing adsorptive pressure, the amount of additionally adsorbed CO_2_ decreases as the ultramicropores are already filled and adsorption takes place on the outer fiber surface only, as indicated by a flattening of the isotherms (Figure 1a/b). Therefore, the selectivity reaches lower values in this pressure regime as well. However, as the additionally adsorbed amount of CO_2_ and N_2_ at elevated pressures is comparatively low (Figure 2), the selectivity appears to attain a constant value at higher pressures.

In consideration of a constant adsorptive pressure, the same argumentation can be applied to the explanation for the influence of the feed gas composition observed at low pressures as the CO_2_ partial pressure is proportional to its volume fraction. Following the reasoning above, small CO_2_ fractions in the feed gas are favorable for the investigated material, since smaller CO_2_ fractions yield higher selectivity values due to the low partial pressure of CO_2_. This finding and the high CO_2_ capacity at low pressures suggests that PAN‐based carbon nanofibers are most suitable for the capture of CO_2_ from flue gases with low CO_2_ content.

A common method to predict selectivity values from pure component isotherms are IAST calculations. The result of IAST calculations based on the isotherms shown in Figure 1a,c are given in Table 2 and Figure S9 in comparison to some of the measured selectivity values depicted in Figure 6. The comparison of calculated and measured values shows the similar asymptotic curve progression of the selectivity due to an increase in pressure, yielding a qualitative conformity. Moreover, the IAST calculations predict the same gradual decline in selectivity for the sample carbonized at 600 °C to the sample carbonized at 900 °C that was observed in the measurement. The fact that the IAST calculations predict this decline correctly, although the CO_2_ and N_2_ isotherms of the samples prepared at 600, 700, and 800 °C are similar, can be attributed to the low‐pressure uptake of the CO_2_ isotherms. At the chosen conditions, only the low‐pressure section of the CO_2_ isotherm is relevant for the IAST calculations. As shown in Figure S10, the initial uptake of the CO_2_ isotherms declines for an increasing carbonization temperature, which is also reflected in the continuous change of the affinity constant *K* of the Tóth model fits (Table S2). Thus, the predicted selectivity gradually decreases with increasing carbonization temperature as well. As discussed above, the higher initial slope is likely to be a result of the higher nitrogen content in the CNFs.^[23]^

**Table 2:** Comparison of measured and IAST‐predicted CO_2_/N_2_ selectivity, both at 0 °C and for a CO_2_/N_2_ ratio of 5 : 95 and 10 : 90. The experimental runs were performed at an overall pressure of 5 bar. The given pressure of the experimental run refers to the adsorptive pressure (sum of CO_2_ and N_2_ partial pressure).

CO_2_/N_2_ ratio	*T*_C_ [ °C]	0.25 bar		0.5 bar		1.0 bar	
		Exp.	IAST	Exp.	IAST	Exp.	IAST
5 : 95	600	194	275	150	259	138	249
5 : 95	700	144	217	105	206	97	206
5 : 95	800	110	163	84	157	79	158
5 : 95	900	119	59	46	53	52	48
10 : 90	600	148	258	126	248	138	247
10 : 90	700	113	207	93	203	98	214
10 : 90	800	95	156	81	157	76	170
10 : 90	900	66	55	44	49	40	44

*T*_C_: carbonization temperature. Exp.: experiment.

A quantitative analysis reveals that, although the values obtained from the measurements and the IAST calculations are in the same order of magnitude, the prediction by IAST overestimates the selectivity values by a factor of 1.5 to 2.2 for the CNFs carbonized at 600, 700, and 800 °C. As illustrated in Figures S11 and S12, this can be attributed to a higher CO_2_ uptake as well as a lower N_2_ uptake predicted by the IAST compared to the dynamic gas adsorption measurement. This is a known issue of IAST in literature.[^19^, ^48^] In fact, it was reported that IAST calculations might not predict multicomponent adsorption well, if one of the components is much stronger adsorbed than the other, since this results in a clear deviation from the prerequisite ideal solution.[^25^, ^26^] Our data further supports these findings. Moreover, the calculation of the spreading pressure might require an extrapolation beyond the maximum pressure of the statically measured N_2_ isotherms.^[33]^ This effect becomes more predominant for high selectivity values^[26]^ as also presented in this work and could, therefore, account for deviations between the IAST prediction and the measurement, too.

Table 3 provides measured CO_2_/N_2_ selectivity values of several carbonaceous materials reported in literature for comparison. Usually measurements at 1.0 bar and 25 °C with CO_2_ fractions from 10 to 16.7 %, which are close to the CO_2_ fraction in flue gas, are reported,[^47^, ^48^, ^49^, ^50^] although measurements at higher temperatures can be found as well.[^9^, ^51^] As a general observation it can be said that untreated activated carbon materials exhibit a CO_2_/N_2_ selectivity of 4 to 10.[^9^, ^49^, ^51^] In comparison to this, the investigated CNFs show significantly higher values of 50 or more at 25 °C and 1.0 bar, at least for the samples carbonized at 800 °C or below.

**Table 3:** Literature survey regarding the CO_2_/N_2_ selectivity determined experimentally from a binary mixture of CO_2_ and N_2_ for various, CO_2_‐adsorbing, mostly carbonaceous materials.

*T*_c_ or sample	Material	Selectivity	*P* [bar]	*T* [ °C]	CO_2_ frac. [%]	Ref.
600 °C	CNF	106	1.0	25	5	this work
700 °C	CNF	80	1.0	25	5	this work
800 °C	CNF	59	1.0	25	5	this work
900 °C	CNF	34	1.0	25	5	this work
600 °C	CNF	95	1.0	25	10	this work
700 °C	CNF	69	1.0	25	10	this work
800 °C	CNF	53	1.0	25	10	this work
900 °C	CNF	35	1.0	25	10	this work
50CPDA@A‐C	carbon‐asphalt composite	22.1	1.0	25	15	[47]
C141	activated carbon	10.7	1.0	25	15	[51]
Cu‐BTC	MOF	15.0	1.0	25	15	[51]
WV1050	activated carbon	9.0	1.0	25	15	[51]
AP 3–60	activated carbon	10–12^[a]^	20–60	25	10–67	[50]
SNMC‐1–600	activated carbon	4.97	1.0	25	15	[48]
KNC−A‐K	N‐doped carbon	44	1.0	25	10	[49]
SBA−C‐A‐HCl	activated carbon	4.5	1.0	25	10	[49]
RF‐16	resin‐based carbon	1.5	1.1	70	16.7	[9]
RLF‐16	carbon‐Laponite® hybrid	25	1.1	70	16.7	[9]
RLF‐16 act	activated carbon Laponite® hybrid	114.3	1.1	70	16.7	[9]
C141	activated carbon	6.2	1.0	75	15	[51]
Cu‐BTC	MOF	8.0	1.0	75	15	[51]
WV1050	activated carbon	5.4	1.0	75	15	[51]

[a] This result is estimated based on binary adsorption data.

The sample prepared at 600 °C even reaches a selectivity of 106 under these conditions (5 % CO_2_) increasing the selectivity by one order of magnitude compared to the untreated carbon materials. At lower overall pressures, the selectivity for this sample is determined to be even higher and can be as high as 194, outperforming the reported carbon materials. Only carbon materials that have been intensely modified, such as the activated carbon Laponite® composite (114.3),^[9]^ exhibit similar values. However, the CNFs investigated in this paper feature an easier preparation procedure and can readily be produced at industrial scale, due to the availability of large‐scale electrospinning facilities.

## Conclusion

Polyacrylonitrile‐based carbon nanofibers (CNFs) were prepared at different carbonization temperatures ranging from 600 to 1000 °C, and the resulting fibers were analyzed with respect to CO_2_ sorption using dynamic sorption techniques. The fibers carbonized in a temperature range from 600 to 800 °C exhibit an excellent CO_2_/N_2_ selectivity. The best performance was found for the CNFs prepared at 600 °C with measured CO_2_/N_2_ selectivity values of up to 194, while an increase of the carbonization temperature had a negative effect. In this study, the influence of pressure, temperature, and feed gas composition on the CO_2_/N_2_ selectivity was thoroughly investigated. It was found that the CO_2_/N_2_ selectivity is highest for low measurement temperatures and pressures as well as for small CO_2_ fractions in the feed gas. The high selectivity was attributed to a particularly high amount of ultramicropores present on the fibers. Additionally, a lower carbonization temperature resulting in a higher heteroatom content appears to be beneficial for the CO_2_/N_2_ selectivity as it results in a more efficient displacement of N_2_ by CO_2_.

For the samples carbonized at 900 °C a flattening of the CO_2_ breakthrough curves was observed, indicating a slow adsorption rate due to kinetic limitations, which was confirmed by modeling of the breakthrough curves. In contrast, the samples carbonized at lower temperatures do not exhibit kinetic limitations due to a better accessibility of the ultramicropores. Thus, the samples carbonized at 600 and 700 °C are preferable for practical applications such as CO_2_ removal from flue gas due to their fast adsorption and excellent selectivity. Moreover, the CNFs carbonized at 700 °C were tested in a long‐term experiment of more than 300 adsorption and desorption cycles and did not show any degradation, practically indicating the excellent stability of the material.

## Experimental Section

### Synthesis

All chemicals were used as received without further purification. To obtain PAN‐based CNFs, 10 wt% of PAN (150000 g mol^−1^, BOC Science, USA) was dissolved in *N*,*N*‐dimethylformamide (VWR Chemicals, Germany) by stirring for two days at room temperature. Afterwards, the solution was electrospun using an electrospinning device equipped with a rotating drum collector and a climate chamber (IME Technologies, The Netherlands). The tip‐collector distance was 15 cm, the applied voltage was 25 kV, and the flowrate of the polymer solution was set to 40 μL min^−1^. The drum collector had a diameter of 6 cm and rotated at a speed of 1500 rpm. The nozzle with a diameter of 0.8 mm moved laterally with respect to the drum collector in a range of ±60 mm from the central position at a speed of 20 mm s^−1^ and a turn delay of 500 ms. The atmosphere was kept constant at 25 °C and 30 % relative humidity. After 6 h of continuous electrospinning, a PAN‐nanofiber mat was obtained and the polymer chains within the PAN‐nanofibers were crosslinked at 250 °C in air for 12 h in a drying furnace. Subsequently, carbonization was carried out in a tube furnace at various temperatures ranging from 600 to 1000 °C under argon atmosphere. Each sample was heated with 300 K h^−1^ to the desired temperature, which was held for 3 h. To densify the material, about 2 g of the fiber mats was pressed to obtain a pellet of carbon powder using a hydraulic press (20 mm diameter matrix, 50 kN, 5 min). Afterwards, the pellet was chopped into 1–2 mm large pieces that were used for the gas adsorption experiments.

### Sorption

Static isotherms of CO_2_ (4.5, Air Liquide, France) and N_2_ (4.5 Air Liquide, France) were measured on a 3P micro 300 (3P Instruments GmbH, Germany). 0.3–0.6 g of the pressed CNFs was filled into the measurement cell and degassed for 24 h under vacuum at 150 °C. After degassing the sample mass was quantified again. The isotherms were measured at 273 K.

IAST calculations for the gas adsorption of a mixture of CO_2_ and N_2_ with the ratios of 5 : 95 and 10 : 90 were performed with the commercial software 3PSim (3P Instruments GmbH, Germany). As input for the calculations Tóth fits of the static CO_2_ and N_2_ isotherms (Figure 1a,c) were used. The parameters of the Tóth fits are given in Table S2 in the Supporting Information.

All breakthrough experiments were performed on a mixSorb SHP (3P Instruments, Germany). Details on the setup can be found in Figure S13. Binary gas mixtures were analyzed with a thermal conductivity detector. Ternary gas mixtures were analyzed using a mass spectrometer (GSD 350, Pfeiffer Vacuum, Germany). About 1.3–1.5 g of CNFs was filled into the measurement column (stainless steel, 15 cm long, 8 mm wide) until a fill level of 8–10 cm was reached. Afterwards, the sample was pre‐treated at 300 °C under 20 mL min^−1^ He and vacuum for at least 1 h to remove adsorbed gases and moisture.

The analysis of the breakthrough was done using the mixSorb Manager (Version 1325, 3P Instruments, Germany). Further information on the analysis is provided in the Supporting Information. Each breakthrough curve was integrated from the start of the experiment until no further change in the gas composition (*c*_in_=*c*_out_) was observed. For the evaluation of all measurements, the variation of the outlet gas stream due to adsorption was accounted for using helium as an internal standard. More details on this procedure are given in the Supporting Information. The adsorption capacity for all measurements was obtained subtracting the integrated value of a blank run from the integrated value of the sample run. A visualization of the evaluation procedure is shown in Figure S14. The blank run was performed using glass beads (VWR Chemicals, Germany) with a diameter of 0.4 to 0.6 mm as a filling material to match the void volume of the blank run with the void volume of the sample run. The result of the blank run was averaged over three measurements.

Dynamic isotherms of CO_2_ (4.5, Air Liquide, France) were measured with helium (6.0, Air Liquide, France) as a carrier gas at 273 K and 10.0 bar. The total flow rate was set to 50 mL min^−1^. For the first breakthrough curve the adsorptive fraction (*v*/*v*) equaled 1 %. After saturation was reached, an additional isotherm point was recorded by increasing the CO_2_ fraction by 1 %. This procedure was performed for subsequent isotherm points until a fraction of 10 % was reached. High‐pressure isotherms of CO_2_ and N_2_ (4.5, Air Liquide, France) were recorded in a similar manner. Unlike before, these measurements were performed at a total pressure of 45 bar and the adsorptive fraction was increased in 5 % steps up to 40 % resulting in a CO_2_ partial pressure ranging from 2.25 to 18 bar. Dynamic isotherms of N_2_ up to 1 bar were acquired measuring five separate breakthrough curves from 0 % to 4, 8, 12, 16, and 20 % N_2_ in He, respectively. Between each breakthrough experiment, the sample was regenerated by 20 mL min^−1^ He and vacuum at 273 K for 60 min. The breakthrough curves of these measurements are shown in Figures S15 to S18.

Modeling of the breakthrough curves was carried out using the commercial software 3P Sim (3P Instruments GmbH). Details on the applied formulas can be found in the Supporting Information.

The selectivity of the CO_2_/N_2_ adsorption was measured at 5 bar and a flow rate of 100 mL min^−1^ with helium as the carrier gas. The investigated gas compositions are given in Table S3. After each breakthrough experiment, the desorption curve was recorded under the same conditions but with CO_2_ and N_2_ turned off. The desorption curves were measured and integrated over 120 min. Afterwards, the sample was further regenerated applying vacuum and 20 mL min^−1^ He for 30 min. The breakthrough curves are shown in the Supporting Information.

The long‐term sorption performance was tested for CNFs carbonized at 700 °C (0.3 g) by consecutive ad‐ and desorption steps. For this, over 300 cycles of adsorption and desorption were performed. 5 % CO_2_ in N_2_ at 25 °C and a total pressure of 10 bar were used for adsorption, whereas desorption was carried out at a pressure of 1 bar with pure N_2_. In both cases, the flow rate was 50 mL min^−1^. A complete list of the conditions of the dynamic measurements is given in the Supporting Information in Tables S3 and S4.

## Conflict of interest

The authors have patent #WO2020249441A1 issued to Forschungszentrum Jülich.

1

## Supporting information

As a service to our authors and readers, this journal provides supporting information supplied by the authors. Such materials are peer reviewed and may be re‐organized for online delivery, but are not copy‐edited or typeset. Technical support issues arising from supporting information (other than missing files) should be addressed to the authors.

Supporting Information

## Data Availability

The data that support the findings of this study are available from the corresponding author upon reasonable request.
