# Supplementary material for: CO2/N2 Separation on Highly Selective Carbon Nanofibers Investigated by Dynamic Gas Adsorption
Source: ChemSusChem. 2022 May 24;15(14):e202200761. doi: 10.1002/cssc.202200761 (PMC9401035; doi:10.1002/cssc.202200761)
Supplement: Supplementary file 1 — Supporting Information [file CSSC-15-0-s001.pdf]

ChemSusChem

Supporting Information

CO_2/N_2 Separation on Highly Selective Carbon Nanofibers Investigated by Dynamic Gas Adsorption

Victor Selmert,* Ansgar Kretzschmar, Henning Weinrich, Hermann Tempel, Hans Kungl, and Rüdiger-A. EichelThis publication is part of a collection of invited contributions focusing on "The Fuel Science Center-Adaptive Conversion Systems for Renewable Energy and Carbon Sources". Please visit to view all contributions.© 2022 The Authors. ChemSusChem published by Wiley-VCH GmbH. This is an open access article under the terms of the Creative Commons Attribution License, which permits use, distribution and reproduction in any medium, provided the original work is properly cited.

Results – Dynamic Isotherms

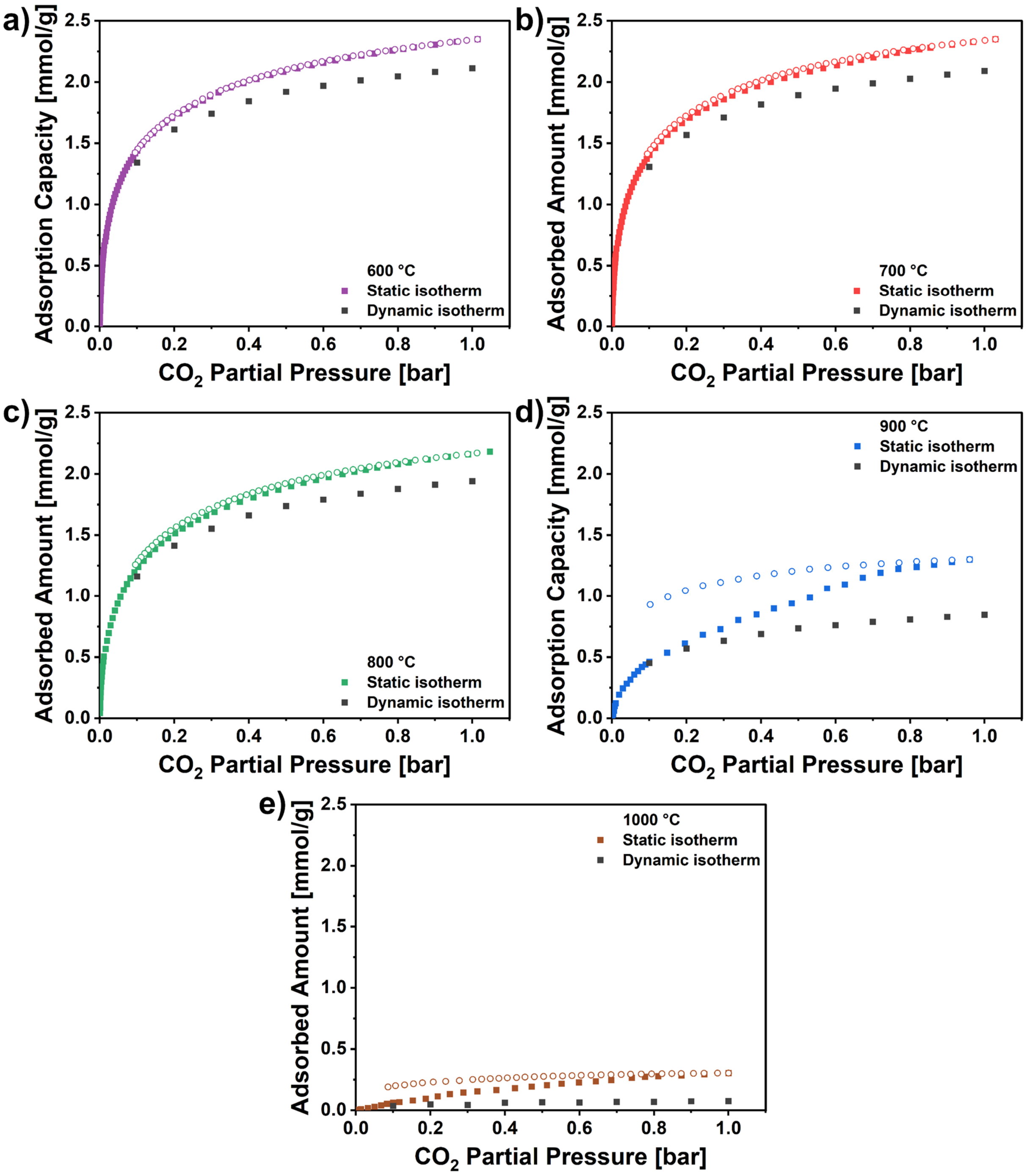

Figure S1: Adsorption isotherms of CO_2 measured with the static and dynamic method at 273 K on PAN-based carbon nanofibers carbonized at various temperatures ranging from 600 °C to 1000 °C.

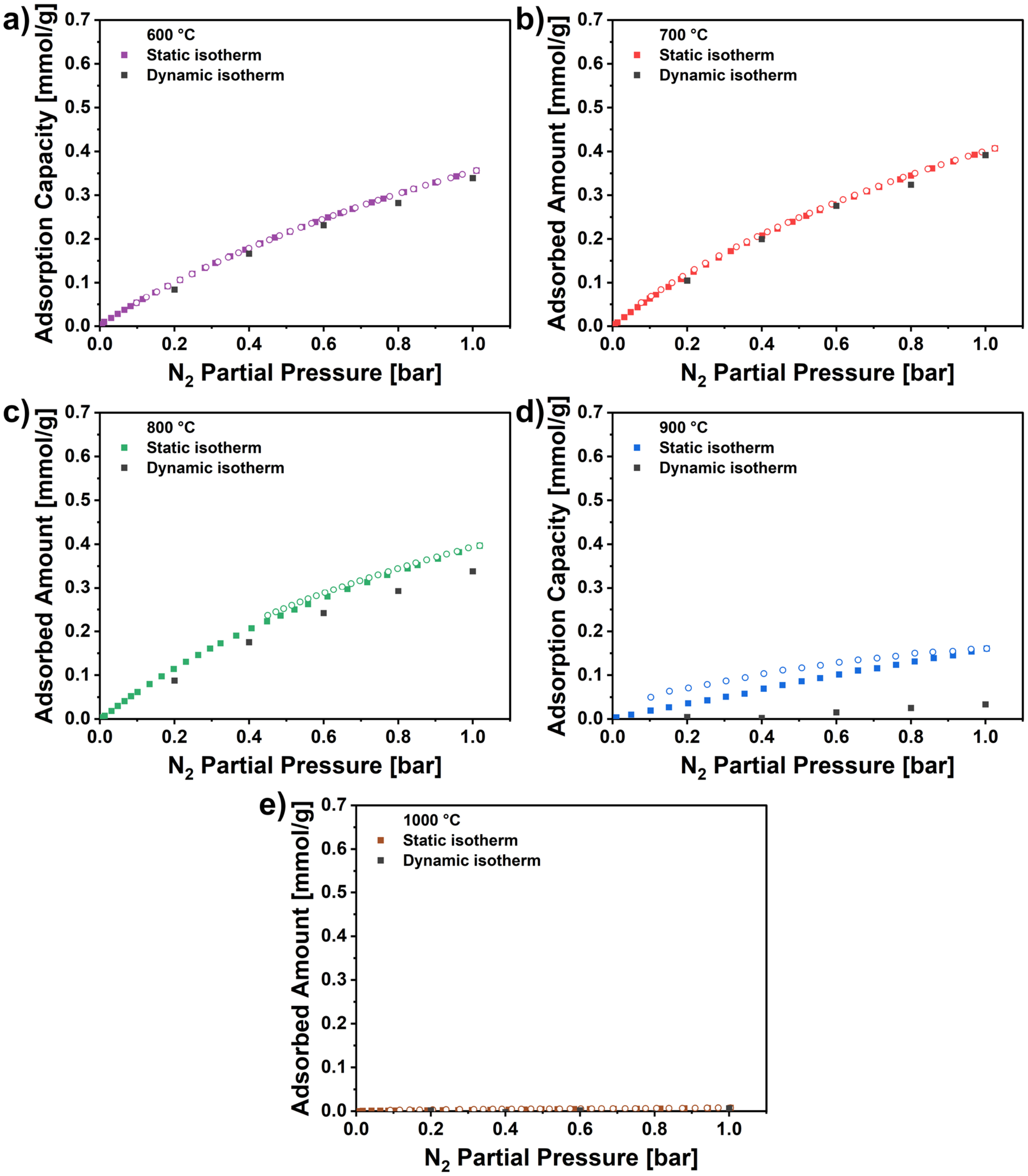

Figure S2: Adsorption isotherms of N_2 measured with the static and dynamic method at 273 K on PAN-based carbon nanofibers carbonized at various temperatures ranging from 600 °C to 1000 °C.

Results - Adsorption Kinetics

Simulation of the Breakthrough Curves

For the modelling of the breakthrough curves the following formulas were used by the 3P Sim software according to the manual.[1]
Mass transfer:

$$\frac{\partial c}{\partial t} = D\frac{\partial^2 c}{\partial z^2} - u\frac{\partial c}{\partial z} - c\frac{\partial u}{\partial z} - \frac{1-\epsilon}{\epsilon}\rho_p\frac{\partial q}{\partial t}$$

Linear driving force assumption:

$$\frac{\partial q}{\partial t} = k_{\mathrm{LDF}}\left(q_{\mathrm{eq}} - \overline{q}\right)$$

Calculation of the gas velocity by ideal gas law:

$$\frac{\partial u}{\partial z} - \frac{RT}{p}\frac{1-\epsilon}{\epsilon}\rho_p\sum_{i=1}^{\mathrm{component}}\frac{1}{M_i}\frac{\partial q_i}{\partial t} = 0$$

Boundary and initial conditions:

$$c(0,t) = c_{\mathrm{in}}$$

$$c(x,0) = 0$$

$$\frac{\partial c}{\partial z_{z=L}} = 0$$

$$u(0,t) = u_{\mathrm{in}}$$

$$u(x,0) = 0$$

$$\frac{\partial u}{\partial z_{z=L}} = 0$$

Tóth Isotherm:

$$q_{\mathrm{eq}} = q_{\mathrm{max}}\frac{K\,p}{(1+(K\,p)^t)^{\frac{1}{t}}}$$

Calculation of the bulk density (density of the fixed bed):

$$\rho_{\mathrm{b}} = \frac{m_{\mathrm{sample}}}{V_{\mathrm{bulk}}}$$

Calculation of the apparent density (particle density):

$$\rho_{\mathrm{p}} = \rho_{\mathrm{b}}\frac{1}{1-\epsilon_{\mathrm{bulk}}}$$

Calculation of the skeleton density (density of the solid):

$$\rho_{\mathrm{solid}} = \rho_{\mathrm{p}}\frac{1}{1-\epsilon_{\mathrm{p}}}$$

Approximation of axial dispersion by W. Kast for particle sizes smaller 3 mm:

$$D = u\,\frac{0.3}{2}$$

Symbol	Unit	Parameter
c	Vol. %	Volume fraction of the adsorptive
t	min	Time
z	cm	Coordinate along axis of the column
u	cm min^{-1}	Gas velocity
ϵ		Porosity
ρ_p	g cm^{-3}	Particle density
q	g g^{-1}	Solid phase concentration
k_{LDF}	min^{-1}	Mass transfer coefficient
q_{eq}	g g^{-1}	Equilibrium loading
$\overline{q}$	g g^{-1}	Average solid phase concentration of a particle
R	J mol^{-1} K^{-1}	Universal gas constant
T	K	Temperature
p	bar	Pressure
M_i	g mol^{-1}	Molar mass of *i*
L	cm	Length of the adsorber column
q_{max}	g g^{-1}	Maximum loading of the Tóth isotherm model
K	bar^{-1}	Affinity constant of the Tóth isotherm model
t		Tóth Exponent of the Tóth isotherm model
m_{sample}	g	Mass of the adsorbent in the column
V_{bulk}	cm^3	Volume of the column
ρ_b	g cm^{-3}	Bulk density
ϵ_{bulk}		Bulk/Bed porosity
ρ_{solid}	g cm^{-3}	Skeletal density
ϵ_p		Particle porosity
D	cm^2 min^{-1}	Axial dispersion coefficient

Table S1: List of the Tóth parameters obtain from a fit with the Tóth isotherm model of the dynamic isotherms in Figure 1b.

Carbonization Temperature [°C]	600	700	800	900
K [bar^{-1}]	103.63	64.46	57.64	41.80
q_{max} [mmol g^{-1}]	2.903	2.820	2.786	1.484
t	0.4192	0.4594	0.4362	0.3825

Influence of the Flow Rate on the estimated CO_2 Uptake

In another set of experiments, breakthrough curves with 3% CO_2 in He at 5bar and 0°C were recorded with various flow rates (25. 50, 100, 175 mL/min) for adsorption and desorption were recorded (with 0.3 g sample mass). As a result of the different flowrates, the period of time within which the CO_2 adsorption or desorption takes place was varied. At high flow rates and slow adsorption kinetics, the admitted CO_2 cannot completely be adsorbed during the residence time in the column and CO_2 breaks through before the sample is fully saturated. Thus, in case of a slow adsorption rate, a decline of the amount of adsorbed gas with increasing flow rate should be observed. The results of the corresponding experiments are shown in Figure S3. In Figure S3a, for the CNFs carbonized at 600 °C to 800 °C almost constant adsorption results with minor fluctuations of less than 0.1 mmol/g adsorbed CO_2 are found, but no clear decrease of the CO_2 adsorption capacity for an increasing flow rate is observed. In this experiment, again, only the sample carbonized at 900 °C exhibits significant kinetic limitations as a

clear decline for the adsorbed amount of CO_2 from 0.87 mmol/g at 25 mL/min to 0.59 mmol/g at 175 mL/min is observed.

In contrast, during the desorption experiments shown in Figure S3b, no changes in the sorption capacity depending on the flow rate are visible. Therefore, adsorption and desorption of the samples carbonized in a temperature range from 600 °C to 800 °C match each other as displayed in Figure S4. In contrast, the CNFs carbonized at 900 °C exhibit a growing discrepancy between the desorption and the adsorption for an increasing flow rate as the adsorbed amount of CO_2 decreases, but the desorbed amount remains constant. The apparent contradiction of more gas desorbing than adsorbing can be referred to the period after the complete breakthrough occurred, but before desorption was performed. In this time span, the adsorption conditions were kept constant for 20 to 30 minutes to reach a stable state. CO_2 that is slowly adsorbed in this period is not included in the integration of the breakthrough curve, as the rate is too small to be distinguished from fluctuation. The absence of a visible limitation during desorption might be masked by the longer desorption period compared to the adsorption period.

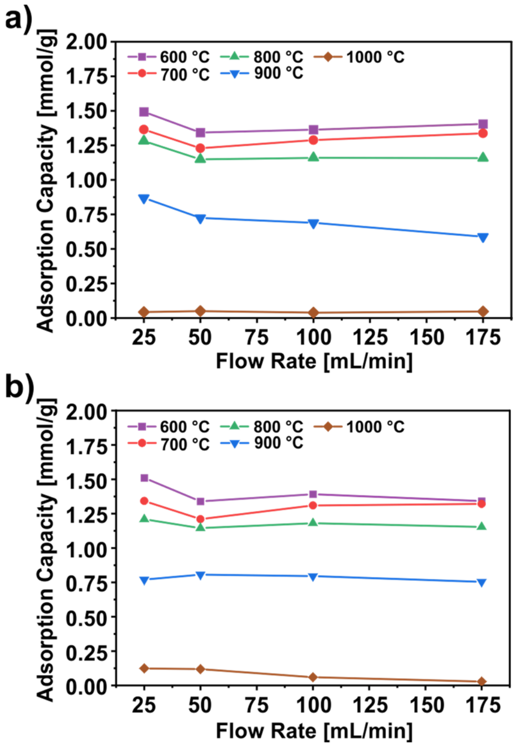

Figure S3: CO_2 adsorption capacity of PAN-based CNFs carbonized at various temperatures ranging from 600 °C to 1000 °C in dependence of the applied flowrate. The measurements were performed applying the dynamic sorption method with 3% CO_2 in Helium at 5 bar and 0 °C. a: Adsorption. b: Desorption

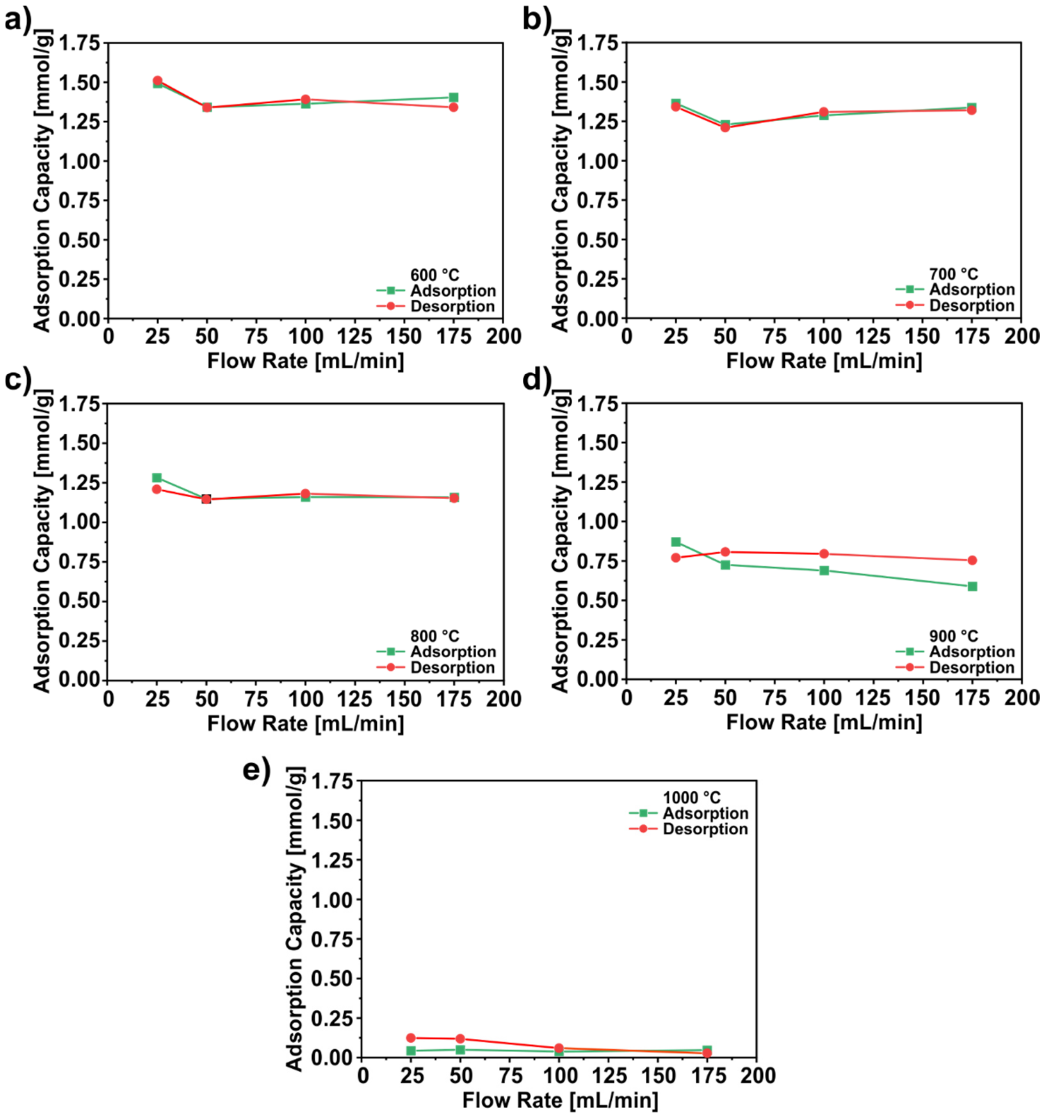

Figure S4: Comparison of the amount of CO_2 adsorbed (green) and desorbed (red) at different flowrates. The adsorption has been performed with 3 % CO_2 in Helium at 5 bar and 0 °C. Afterwards desorption was measured by changing the gas composition to 100 % Helium.

Results – CO_2/N_2 Selectivity

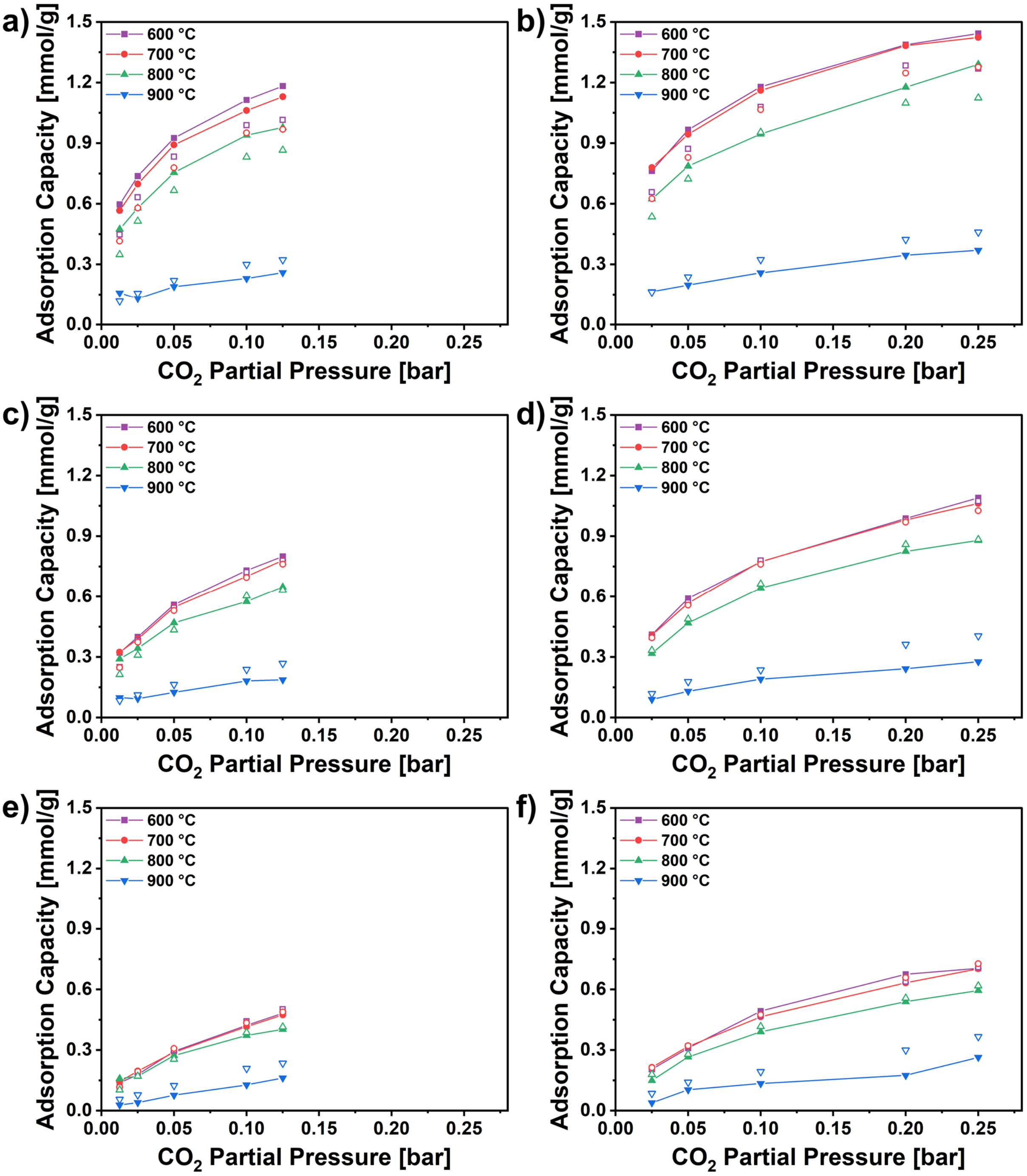

Figure S5: Loading of CO_2 in presence of N_2 measured with the dynamic gas adsorption method and a gas phase composition of 5:95 (left) and 10:90 (right). The measurements were performed with Helium as carrier gas at 5 bar and 0 °C (top), 25 °C (middle) and 50 °C (bottom). Values quantified from the breakthrough curves are filled, whereas values estimated from the desorption curves are open.

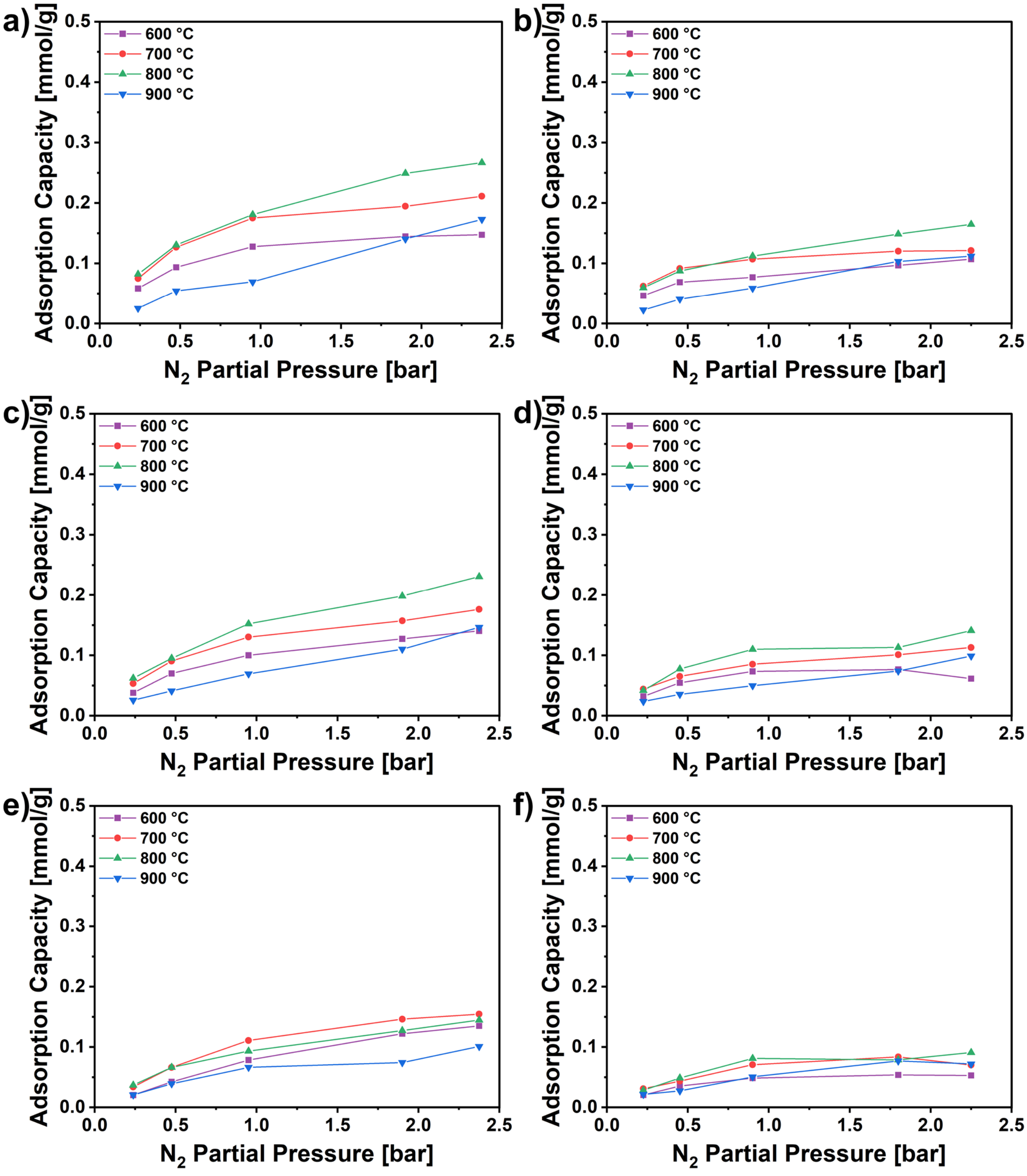

Figure S6: Loading of N_2 in presence of CO_2 measured with the dynamic gas adsorption method and a gas phase composition of 95:5 (A, C, E) and 90:10 (B, D, F). The measurements were performed with Helium as carrier gas at 5 bar and 0 °C (A, B), 25 °C (C, D) and 50 °C (E, F). The values are obtained from the desorption curves. For CNFs carbonized at 900 °C there seems to be a contraction with the high-pressure isotherm, since the amount of N_2 adsorbed in this figure exceeds the adsorption capacity of the high-pressure isotherm in Figure 2. This deviation can be attributed to the different time scales of the experiments. For the high-pressure isotherm of N_2, saturation is seemingly reached after 5 min to 10 min (Figure S17) as the very small adsorption rates of this sample are concealed by fluctuations of the mass flow controller of N_2. However, small amounts of N_2 might still adsorb during the saturated phase of the experiment. In contrast, during desorption the mass flow controller of N_2 is switched off and thus, the desorption curves are integrated over 120 min as very small desorption rates can still be detected as illustrated in Figure S7. As a result, the amount of N_2 estimated by desorption is higher than by adsorption. A similar effect is apparent for CO_2 on these CNFs when the CO_2 capacity quantified by adsorption and desorption in Figure S5 is compared

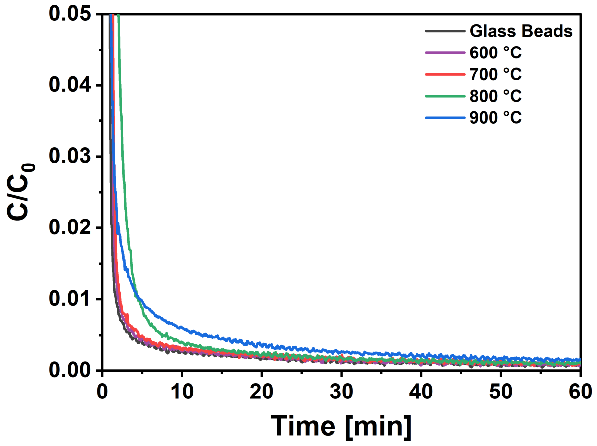

Figure S7: Desorption curves of N_2 measured on carbon nanofibers carbonized at various temperatures ranging from 600 °C to 900 °C with 60 mL/min He after adsorption of 2% CO_2 and 38% N_2 in He at 298 K, 5 bar and a flowrate of 100 mL/min. The sample prepared at 900 °C exhibits a disperse curve with slow desorption rate over a long period of time.

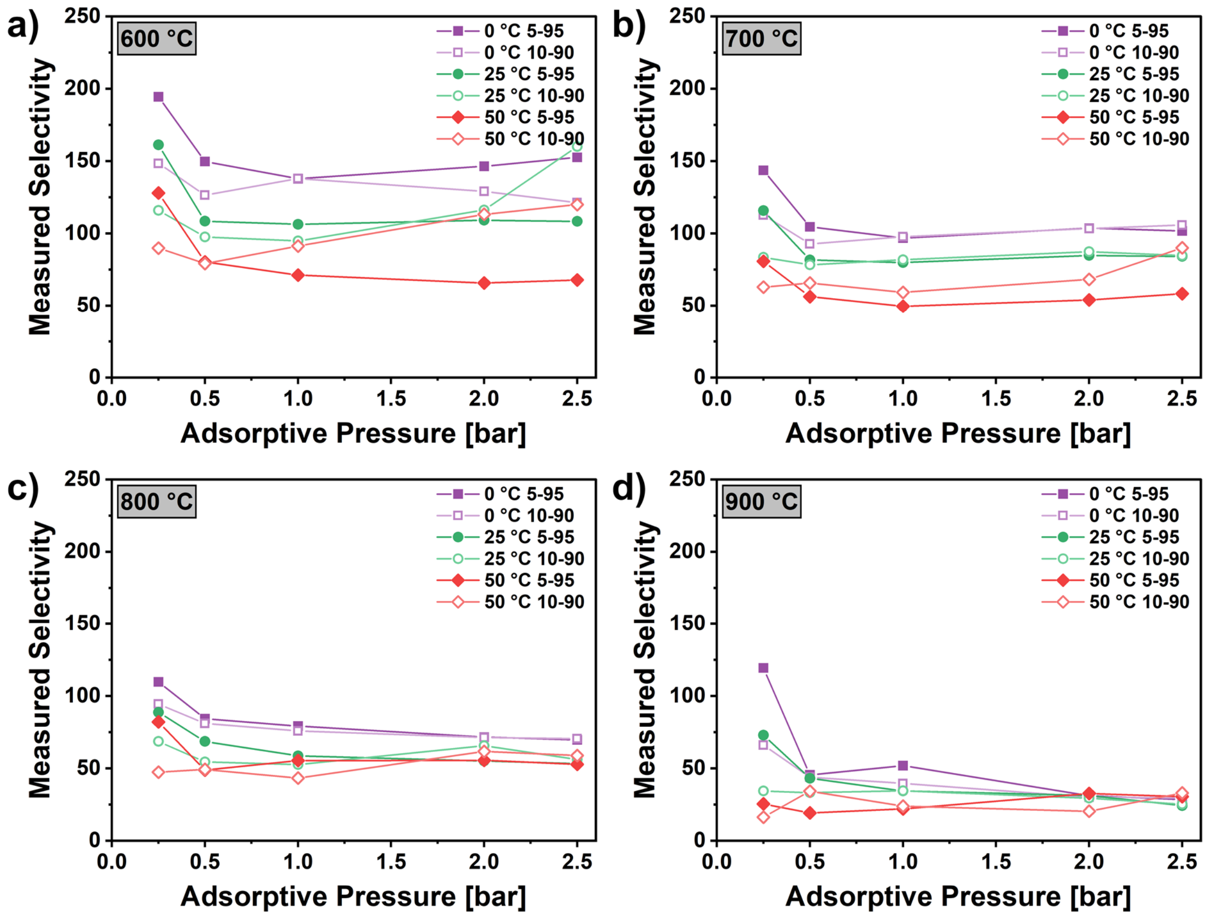

Figure S8: Measured CO_2/N_2 selectivity of PAN-based CNFs carbonized at 600 °C (A), 700 °C (B), 800 °C (C) and 900 °C (D) in dependence of the CO_2 fraction and measurement temperature. The adsorptive pressure is the sum of CO_2 and N_2 partial pressure.

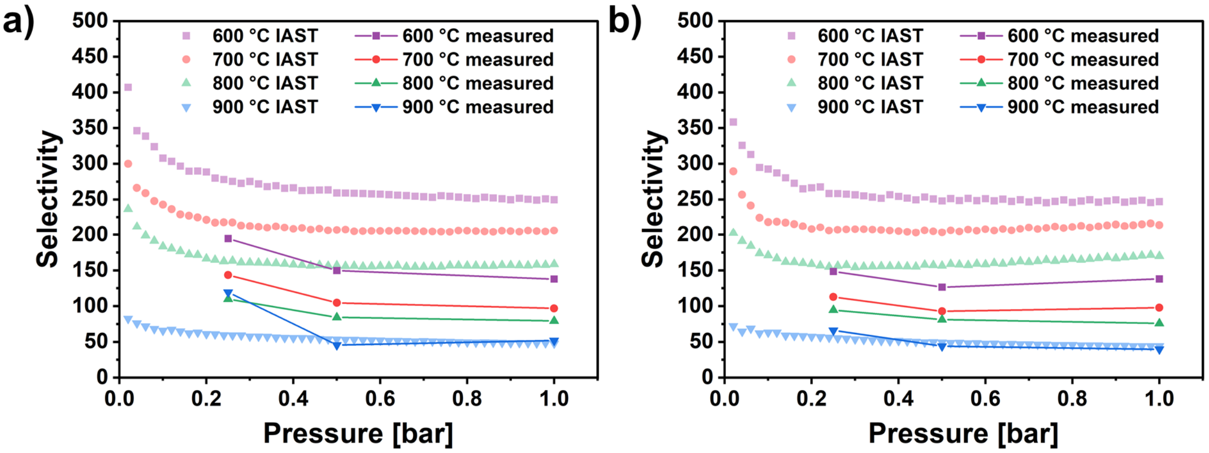

Figure S9: Comparison of the CO_2/N_2 selectivity calculated with the IAST and measured with dynamic gas adsorption at CO_2/N_2 ratios of 5:95 (A) and 10:90 (B) on PAN-based CNFs carbonized at various temperatures. The measurement was performed at 5 bar with He as inert carrier gas. The given pressure refers to the sum of the CO_2 and N_2 partial pressure.

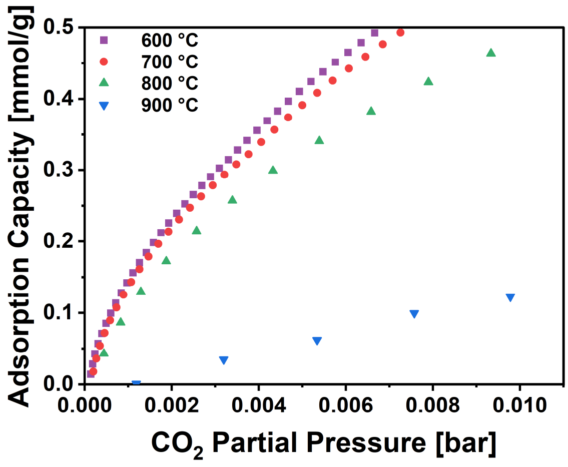

Figure S10: CO_2 isotherms measured with static gas adsorption at 273 K on PAN-based CNFs carbonized at various temperatures ranging from 600 °C to 900 °C. The range up to 0.01 bar is shown to emphasise the higher CO_2 uptake at low pressures for CNFs carbonized at lower temperature.

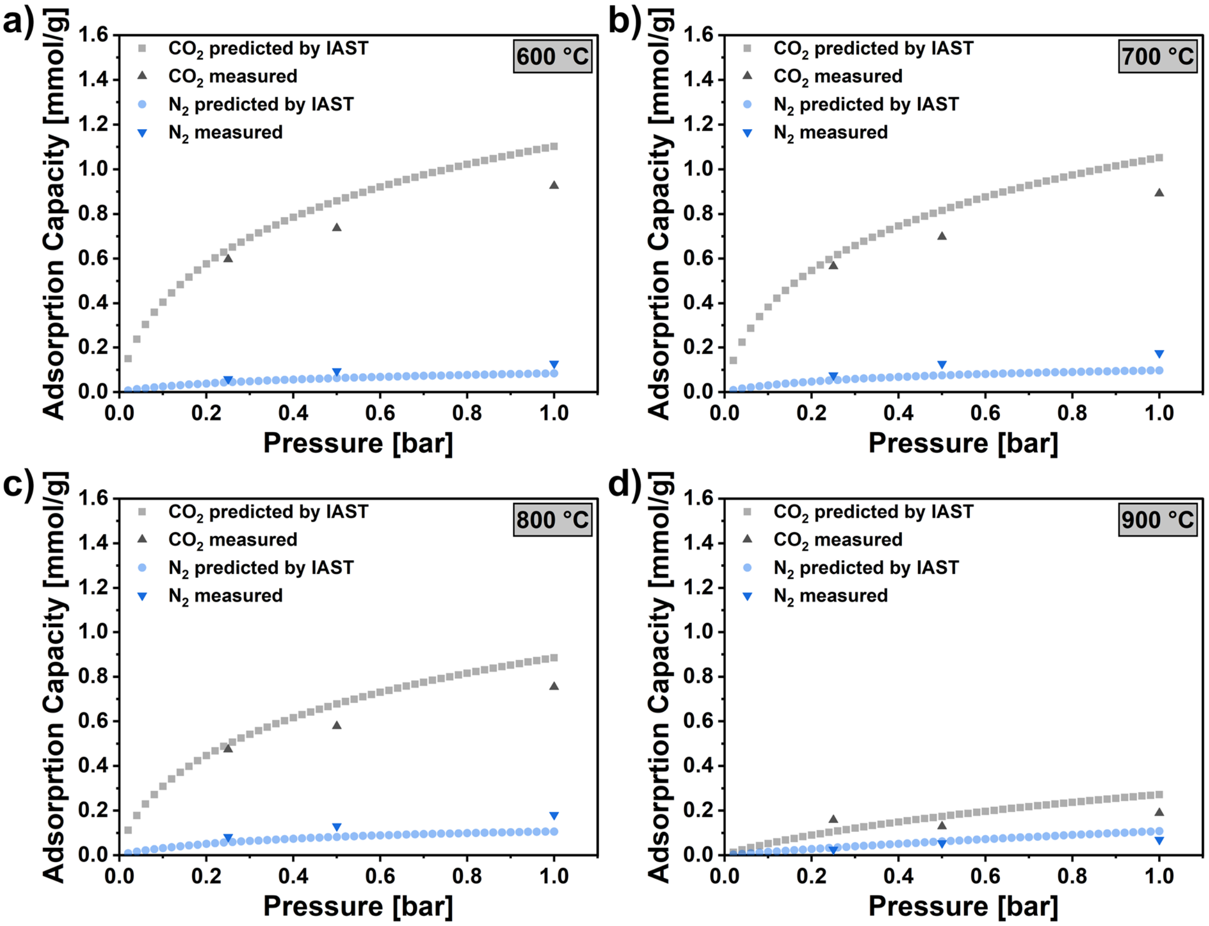

Figure S11: Comparison of the adsorption capacity of CO_2 and N_2 on PAN-based CNFs carbonized at 600 °C (A), 700 °C (B), 800 °C (C) and 900 °C (D) for a gas phase composition of CO_2/N_2 of 5:95 predicted with the IAST and measured with dynamic gas adsorption at 273 K. The measurement was performed at 5 bar with He as inert carrier gas. The given pressure refers to the sum of the CO_2 and N_2 partial pressure.

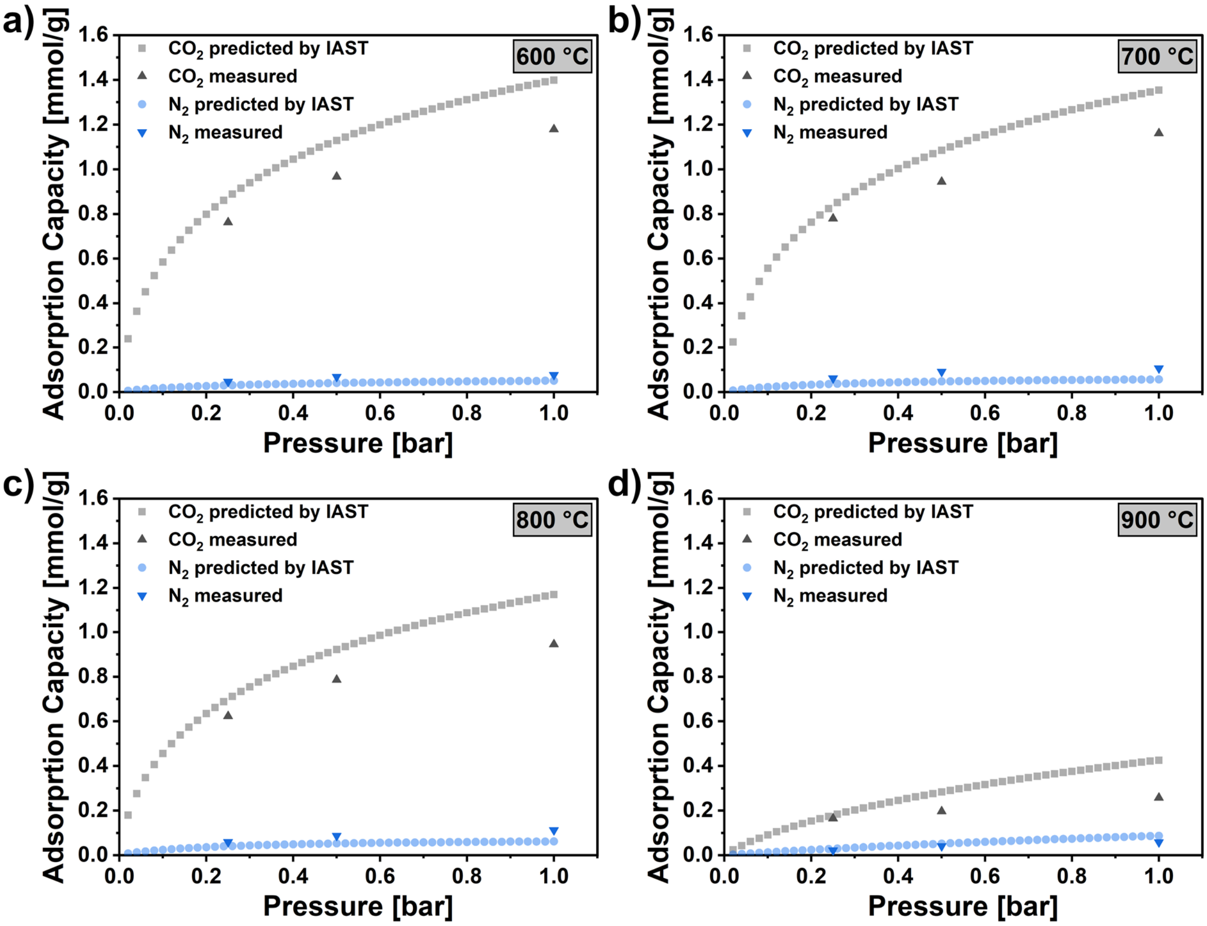

Figure S12: Comparison of the adsorption capacity of CO_2 and N_2 on PAN-based CNFs carbonized at 600 °C (A), 700 °C (B), 800 °C (C) and 900 °C (D) for a gas phase composition of CO_2/N_2 of 10:90 predicted with the IAST and measured with dynamic gas adsorption at 273 K. The measurement was performed at 5 bar with He as inert carrier gas. The given pressure refers to the sum of the CO_2 and N_2 partial pressure.

Experimental Procedure

Table S2: List of the Tóth parameters obtain from a fit with Tóth's isotherm model of the CO_2 isotherms in Figure 1a and the N_2 isotherms in Figure 1c for mixed gas calculations with the IAST.

Carbonization Temperature [°C]	Gas	K [bar^{-1}]	$q_{\max}$ [mmol g^{-1}]	t
600	CO_2	114.6	3.59	0.37078
700	CO_2	105.7	3.72	0.3628
800	CO_2	81.47	3.84	0.3455
900	CO_2	4.678	3.5	0.4223
600	N_2	0.3137	2.00	0.6631
700	N_2	0.4598	1.54	0.7672
800	N_2	0.5594	1.20	0.8681
900	N_2	0.1114	1.59	1

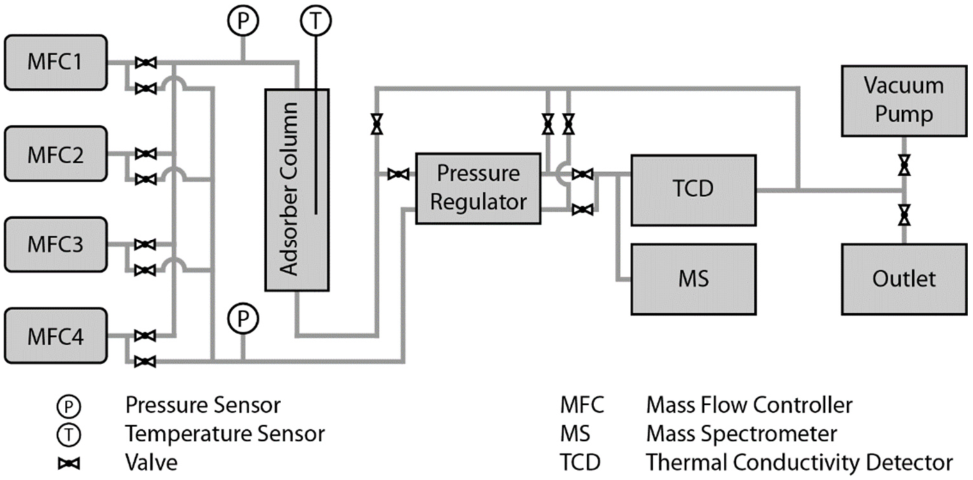

Figure S13: Scheme of the dynamic sorption device (mixSorb SHP, 3P Instruments GmbH). The column has an inner diameter of 8 mm and a length of 15 cm. The temperature sensor extends 9 cm into the column. The back-pressure controller can regulate the pressure between 1.0 and 68 bar with an accuracy of 50 mbar. The mass flow controllers (MFC) are calibrated for N_2 flow and have an accuracy of 0.2 % of the maximum flow. For MFC 1 and 2 the maximum flow equals 10.0 mL/min. For MFC 3 it is 100.0 mL/min and for MFC 4 it is 200.0 mL/min. The flow of gases other than N_2 have been accounted for with a calibration constant (He: 1.5280 (85 bar), CO_2: 0.5786 (50 bar)). The inlet pressure of N_2 and He were 85 bar and the inlet pressure of CO_2 was 50 bar.

Table S3: Gas composition of the selectivity measurements performed at 5.0 bar and temperatures of 0, 25 and 50 °C with a flow rate of 100 mL/min.

	**CO_2/N_2 5:95**			**CO_2/N_2 10:90**		
**Adsorptive Pressure [bar]**	**CO_2 Fraction**	**N_2 Fraction**	**He Fraction**	**CO_2 Fraction**	**N_2 Fraction**	**He Fraction**
0.25	0.25 %	4.75 %	95 %	0.5 %	4.5 %	95 %
0.50	0.5 %	9.5 %	90 %	1.0 %	9.0 %	90 %
1.0	1.0 %	19 %	80 %	2.0 %	18 %	80 %
2.0	2.0 %	38 %	60 %	4.0 %	36 %	60 %
2.5	2.5 %	47.5 %	50 %	5.0 %	45 %	50 %

Table S4: Measurement conditions of all dynamic experiments except the selectivity measurements that are listed in Table S3.

Measurement	$p(CO_2)$ [bar]	$p(N_2)$ [bar]	p(He) [bar]	p_{total} [bar]	Flowrate [mL/min]	Temperature [°C]
Isotherm CO_2 1. point	0.1	0	9.9	10	50	0
Isotherm CO_2 2. point	0.2	0	9.8	10	50	0
Isotherm CO_2 3. point	0.3	0	9.7	10	50	0
Isotherm CO_2 4. point	0.4	0	9.6	10	50	0
Isotherm CO_2 5. point	0.5	0	9.5	10	50	0
Isotherm CO_2 6. point	0.6	0	9.4	10	50	0
Isotherm CO_2 7. point	0.7	0	9.3	10	50	0
Isotherm CO_2 8. point	0.8	0	9.2	10	50	0
Isotherm CO_2 9. point	0.9	0	9.1	10	50	0
Isotherm CO_2 10. point	1.0	0	9.0	10	50	0
Isotherm N_2 1. point	0	0.2	4.8	5.0	100	0
Isotherm N_2 2. point	0	0.4	4.6	5.0	100	0
Isotherm N_2 3. point	0	0.6	4.4	5.0	100	0
Isotherm N_2 4. point	0	0.8	4.2	5.0	100	0
Isotherm N_2 5. point	0	1.0	4.0	5.0	100	0
HP Isotherm CO_2 1. point	2.25	0	42.75	45	100	0
HP Isotherm CO_2 2. point	4.50	0	40.50	45	100	0
HP Isotherm CO_2 3. point	6.75	0	38.25	45	100	0
HP Isotherm CO_2 4. point	9.00	0	36.00	45	100	0
HP Isotherm CO_2 5. point	11.25	0	33.75	45	100	0
HP Isotherm CO_2 6. point	13.50	0	31.50	45	100	0
HP Isotherm CO_2 7. point	15.75	0	29.25	45	100	0
HP Isotherm CO_2 8. point	18	0	27.00	45	100	0
HP Isotherm N_2 1. point	0	2.25	42.75	45	100	0
HP Isotherm N_2 2. point	0	4.50	40.50	45	100	0
HP Isotherm N_2 3. point	0	6.75	38.25	45	100	0
HP Isotherm N_2 4. point	0	9.00	36.00	45	100	0
HP Isotherm N_2 5. point	0	11.25	33.75	45	100	0
HP Isotherm N_2 6. point	0	13.50	31.50	45	100	0
HP Isotherm N_2 7. point	0	15.75	29.25	45	100	0
HP Isotherm N_2 8. point	0	18	27.00	45	100	0
Flowrate variation ads. 1. point	0.15	0	4.85	5.0	25	0
Flowrate variation ads. 2. point	0.15	0	4.85	5.0	50	0
Flowrate variation ads. 3. point	0.15	0	4.85	5.0	100	0
Flowrate variation ads. 4. point	0.15	0	4.85	5.0	175	0
Flowrate variation des. 1. point	0	0	5.0	5.0	24.3	0
Flowrate variation des. 2. point	0	0	5.0	5.0	48.5	0
Flowrate variation des. 3. point	0	0	5.0	5.0	97.0	0
Flowrate variation des. 4. point	0	0	5.0	5.0	169.8	0
Cycle stability: adsorption	0.3	9.7	0	10.0	50	25
Cycle stability: desorption	0	1.0	0	1.0	50	25

Evaluation of the Breakthrough Curves

Integration of the breakthrough curves was performed with the mixSorb Manager (3P Instruments, Version 1325).[1,2] The following equation are used:

$$n_{\mathrm{adsorbed}} = \int \dot{n}_{\mathrm{in}}(t)\,\mathrm{d}t - \int \dot{n}_{\mathrm{out}}(t)\,\mathrm{d}t \tag{1}$$

The equation can be expressed by flow rates and volume fraction as well:

$$n_{\mathrm{adsorbed}} = \int \dot{V}_{\mathrm{in}}(t)\frac{y_{\mathrm{in}}(t)}{V_{\mathrm{m}}}\,\mathrm{d}t - \int \dot{V}_{\mathrm{out}}(t)\frac{y_{\mathrm{out}}(t)}{V_{\mathrm{m}}}\,\mathrm{d}t \tag{2}$$

Usually, the inlet flow and the adsorptive concentration is kept constant during an experiment:

$$n_{\mathrm{adsorbed}} = \frac{\dot{V}_{\mathrm{in}} \cdot y_{\mathrm{in}} \cdot t}{V_{\mathrm{m}}} - \int \dot{V}_{\mathrm{out}}(t)\frac{y_{\mathrm{out}}(t)}{V_{\mathrm{m}}}\,\mathrm{d}t \tag{3}$$

For high diluted experiments with low adsorptive fraction the outgoing flowrate can be considered constant:

$$n_{\mathrm{adsorbed}} = \frac{\dot{V}_{\mathrm{in}}}{V_{\mathrm{m}}}\left(y_{\mathrm{in}} \cdot t - \int y_{\mathrm{out}}(t)\,\mathrm{d}t\right) \tag{4}$$

For high adsorptive fractions the outgoing flowrate can be calculated from an internal standard like Helium that does not adsorb on the adsorbent. Therefore, its flowrate is constant throughout the experiment:

$$\dot{V}_{\mathrm{out}}(t) = \frac{\dot{V}_{\mathrm{internal\ standard}}}{y_{\mathrm{internal\ standard}}\ (t)} = \frac{\dot{V}_{\mathrm{internal\ standard}}}{1 - y_{\mathrm{adsorptive}}\ (t)} \tag{5}$$

n_{adsorbed}	adsorbed amount of gas (mmol/g)
$\dot{n}_i$	molar flux of *i* (mmol/s)
t	time (s)
$\dot{V}_i$	volumetric flowrate (in- or outgoing) (mL/s)
V_{m}	molar Volume (mL/mmol)
y_i	fraction of *i* in the gas phase ()

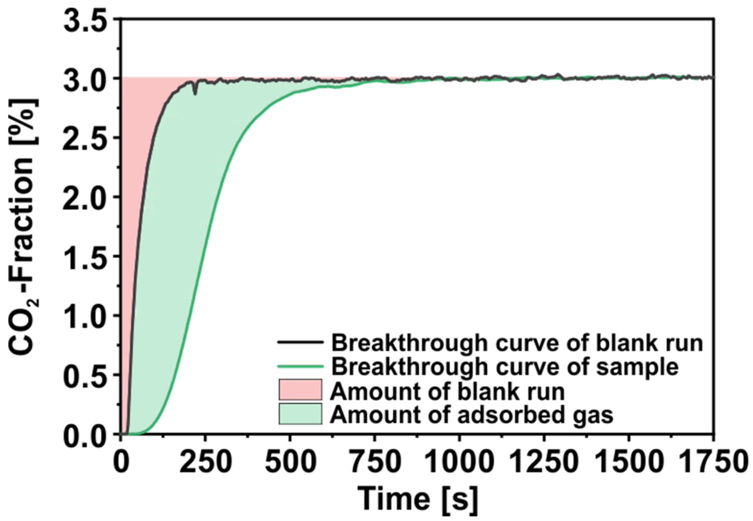

Figure S14: Visualization of the analysis procedure of the breakthrough curves. The adsorbed amount of gas is proportional to the area between the breakthrough curve of the sample and the blank run (green). It is obtained by subtracting the integrated value of the blank run (red area) from the integrated value of the sample run (red plus green area).

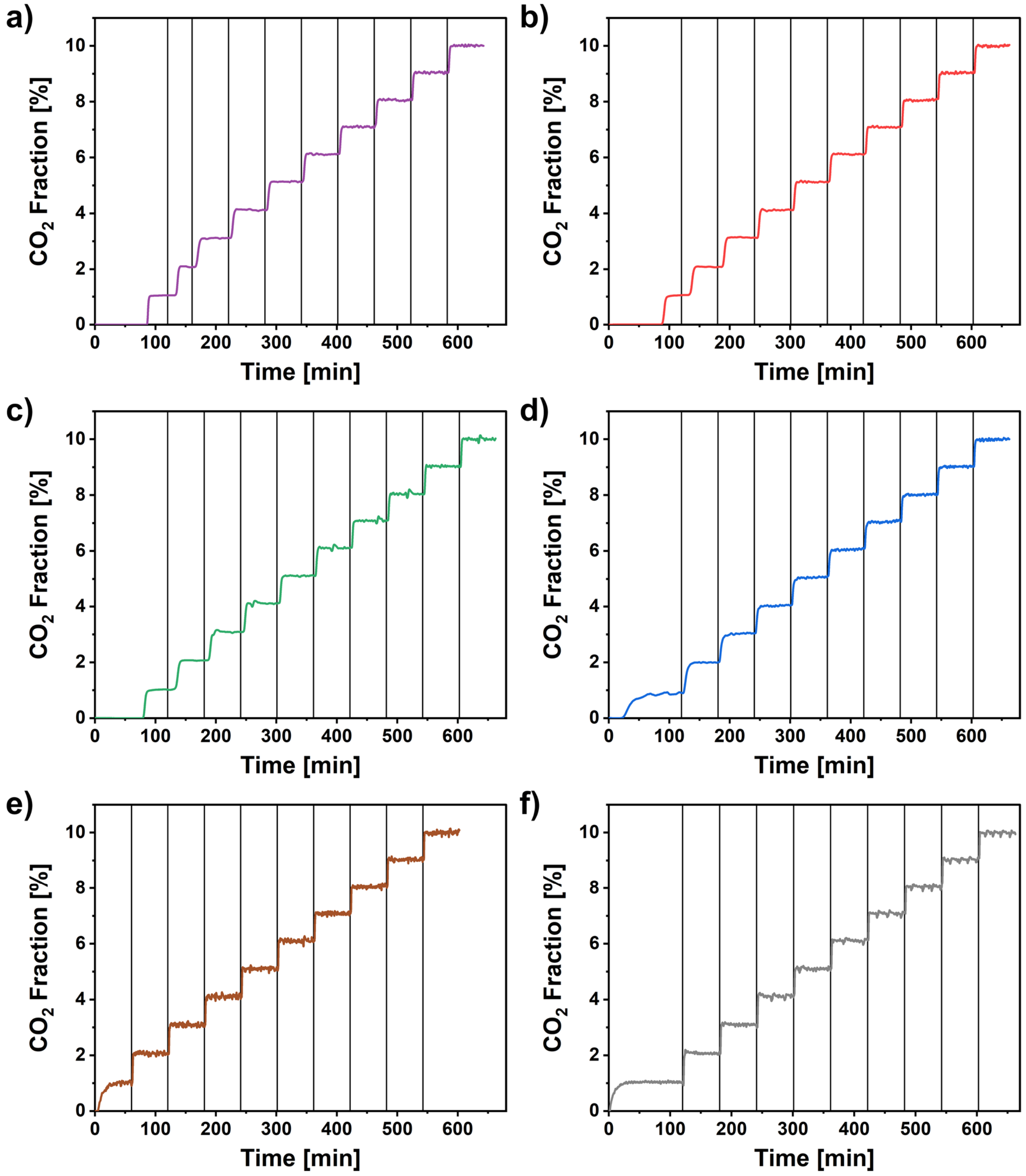

Figure S15: Consecutive breakthrough curves for the measurement of CO_2 isotherms performed with 50 mL/min CO_2 in He at 10 bar and 273 K on PAN-based carbon nanofibers prepared at 600 °C (A), 700 °C (B), 800 °C (C), 900 °C (D) and 1000 °C (E) as well as on glass beads (F). Each line indicates the onset of a new breakthrough experiment. The first two breakthrough curves (0% - 2%) in a) were measured again and origin from a different measurement than the other breakthrough curves (2% - 10%).

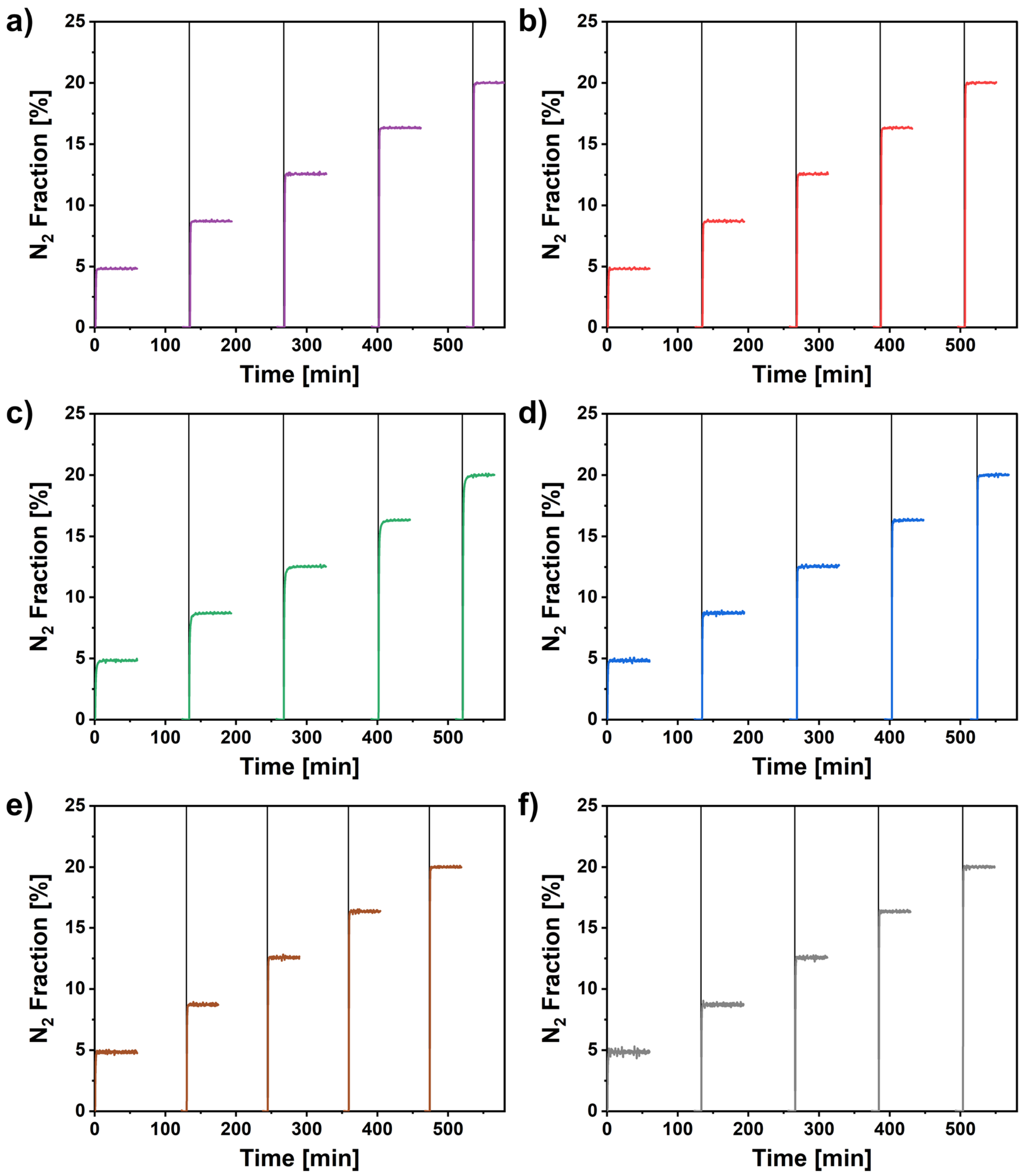

Figure S16: Consecutive breakthrough curves for the measurement of N_2 isotherms performed with 100 mL/min N_2 in He at 5 bar and 273 K on PAN-based carbon nanofibers prepared at 600 °C (A), 700 °C (B), 800 °C (C), 900 °C (D) and 1000 °C (E) as well as on glass beads (F). Each line indicates the onset of a new breakthrough experiment. Between each experiment the sample was regenerated with a flow of 20 mL/min He and vacuum over 60 min.

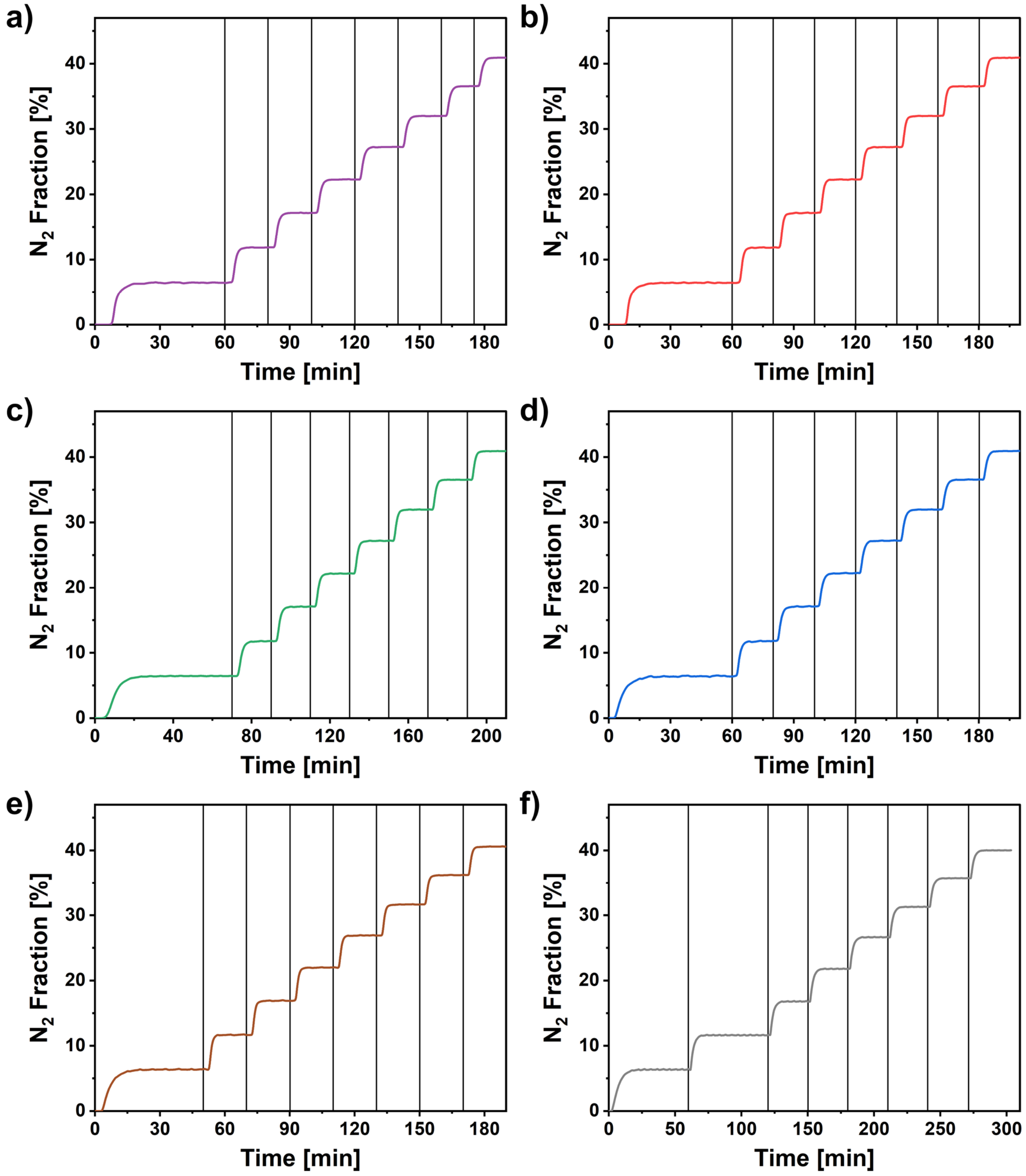

Figure S17: Consecutive breakthrough curves for the measurement of high-pressure N_2 isotherms performed with 100 mL/min N_2 in He at 45 bar and 273 K on PAN-based carbon nanofibers prepared at 600 °C (A), 700 °C (B), 800 °C (C), 900 °C (D) and 1000 °C (E) as well as on glass beads (F). Each line indicates the onset of a new breakthrough experiment.

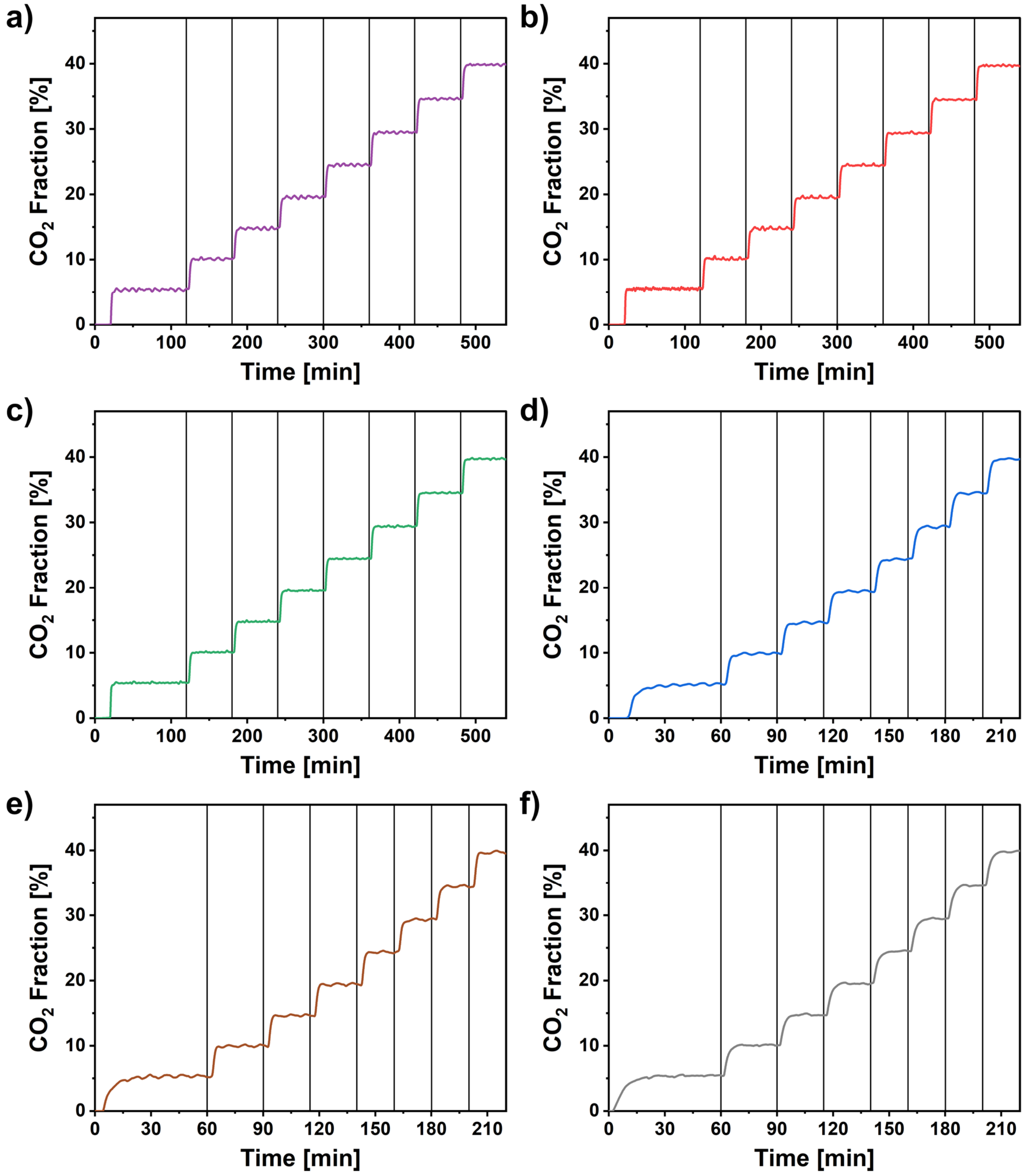

Figure S18: Consecutive breakthrough curves for the measurement of high-pressure CO_2 isotherms performed with 100 mL/min CO_2 in He at 45 bar and 273 K on PAN-based carbon nanofibers prepared at 600 °C (A), 700 °C (B), 800 °C (C), 900 °C (D) and 1000 °C (E) as well as on glass beads (F). Each line indicates the onset of a new breakthrough experiment.

[1] 3P Instruments GmbH, *mixSorb L, mixSorb S, mixSorb SHP, mixSorb Manager*. *Dynamic Sorption Analyzer Operating Manual*, Odelzhausen, **2020**.
[2] 3P Instruments GmbH, "Determination of Mixture Adsorption Equilibrium Data from Breakthrough Curves", to be found under https://www.dynamicsorption.com/dynamic-sorption-method/equilibria/, **2021**.

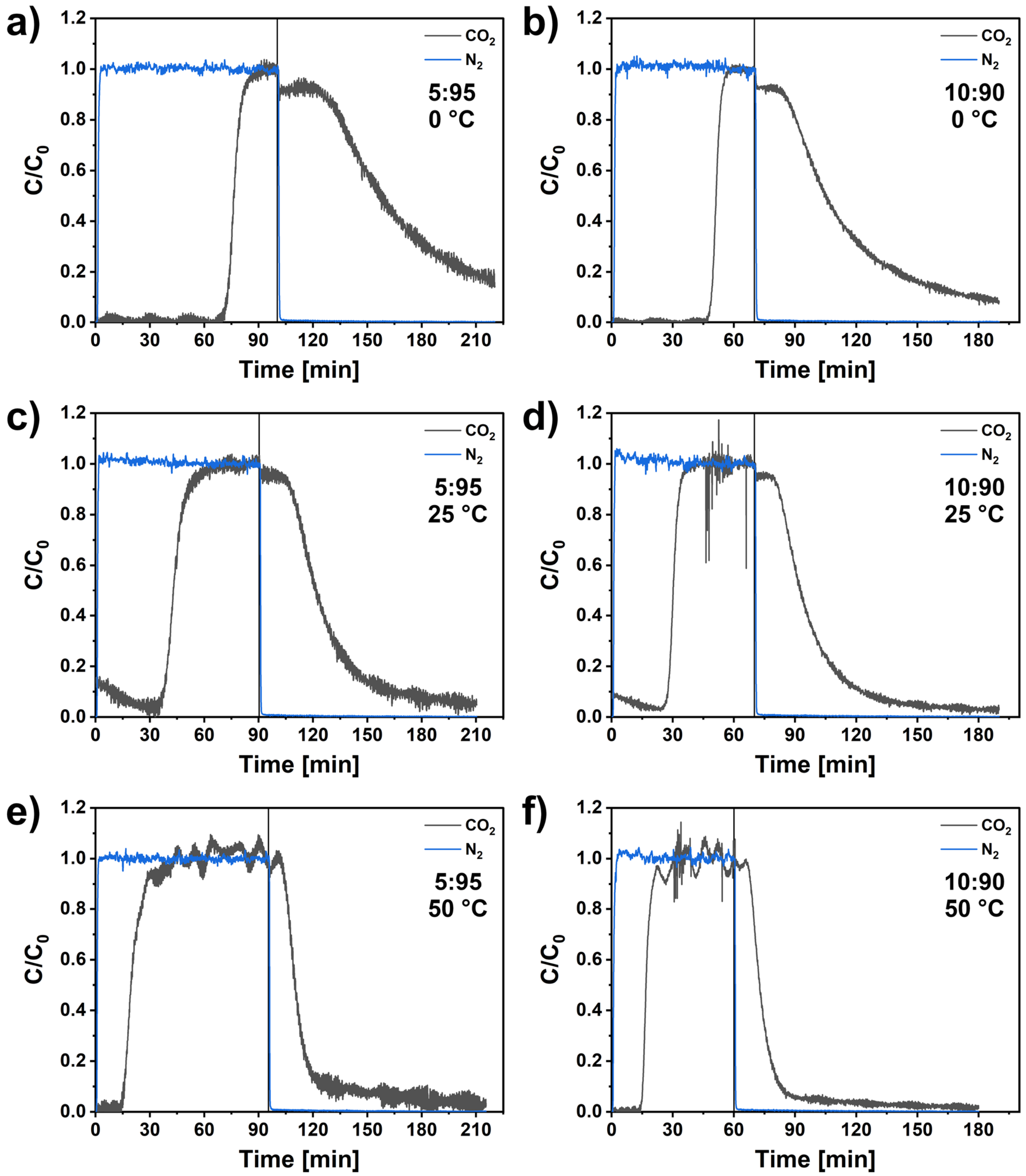

Figure S2-1: CO_2 and N_2 breakthrough curves of a gas mixture with 5:95 (a, c, e) and 10:90 (b, d, f) CO_2:N_2 at 0 °C (a, b), 25 °C (c, d) and 50 °C (e, f) measured on PAN-based CNFs carbonized at 600 °C. The measurements were performed at 5 bar overall pressure and with a flowrate of 100 mL/min. For a gas composition CO_2:N_2 of 5:95, 0.25% CO_2 and 4.75% N_2 (0.25 bar adsorptive pressure) in He were used and for a gas composition CO_2:N_2 of 10:90, 0.5% CO_2 and 4.5% N_2 (0.25 bar adsorptive pressure) in He were used.

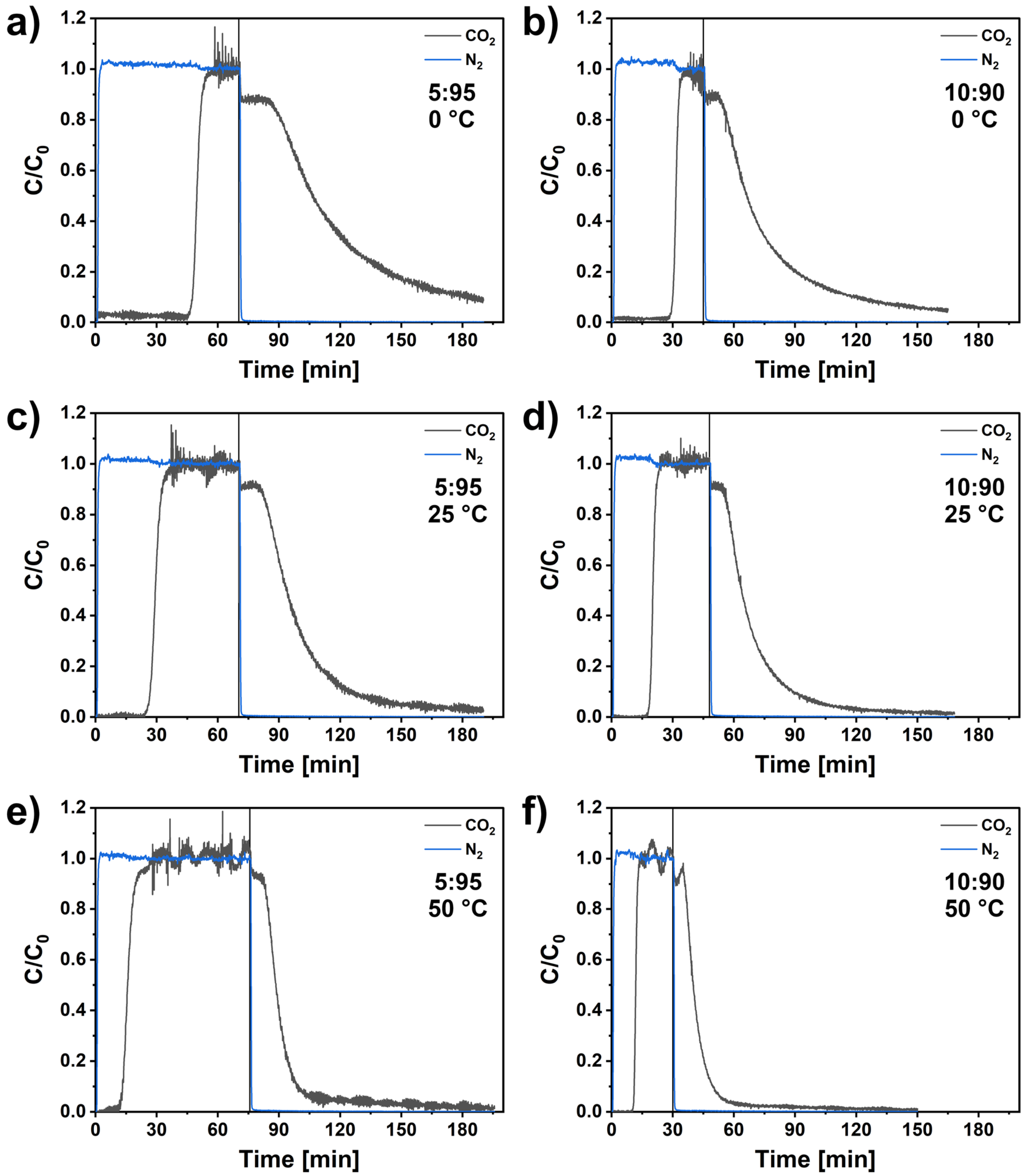

Figure S2-2: CO_2 and N_2 breakthrough curves of a gas mixture with 5:95 (a, c, e) and 10:90 (b, d, f) CO_2:N_2 at 0 °C (a, b), 25 °C (c, d) and 50 °C (e, f) measured on PAN-based CNFs carbonized at 600 °C. The measurements were performed at 5 bar overall pressure and with a flowrate of 100 mL/min. For a gas composition CO_2:N_2 of 5:95, 0.5% CO_2 and 9.5% N_2 (0.5 bar adsorptive pressure) in He were used and for a gas composition CO_2:N_2 of 10:90, 1% CO_2 and 9% N_2 (0.5 bar adsorptive pressure) in He were used.

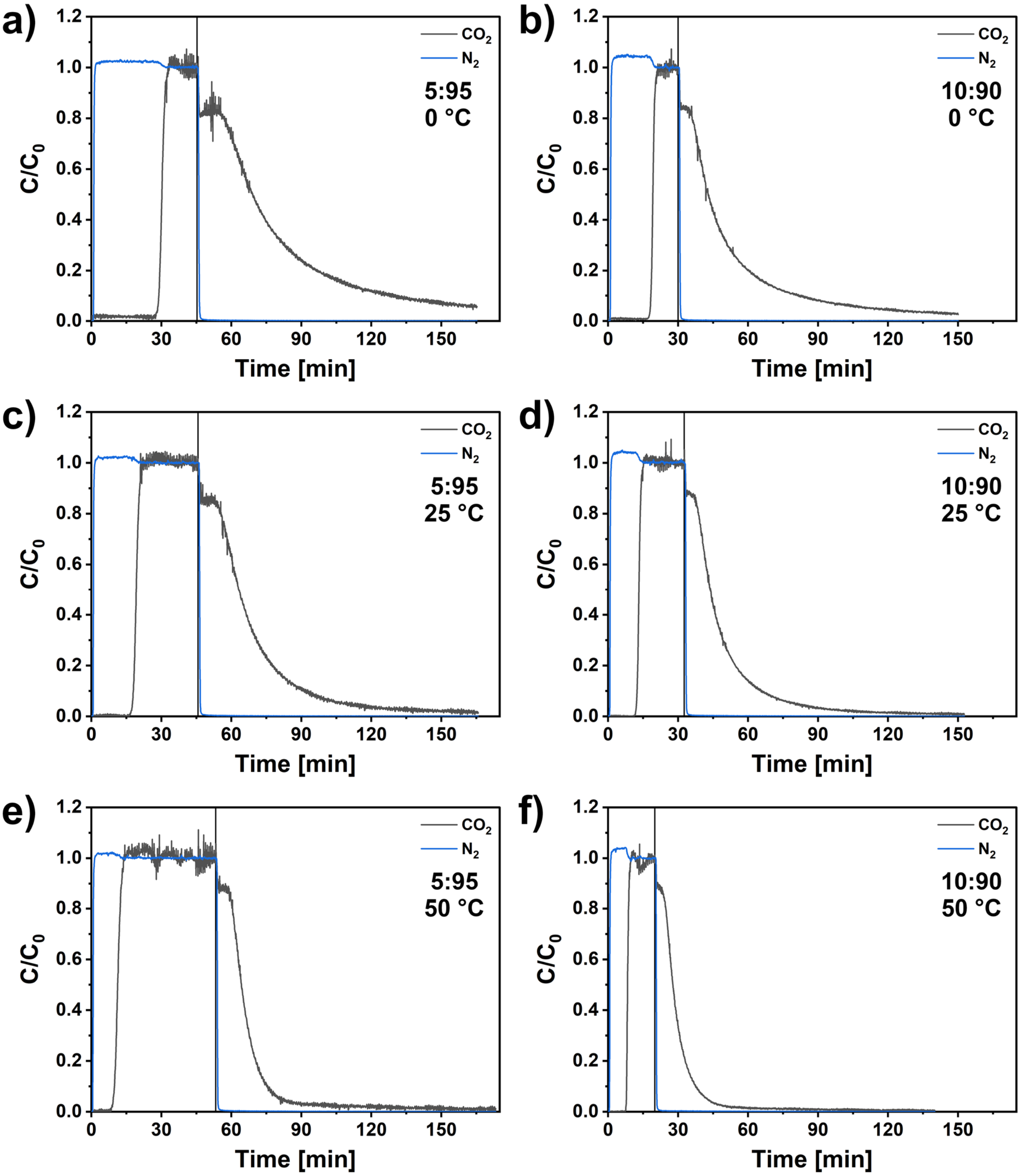

Figure S2-3: CO_2 and N_2 breakthrough curves of a gas mixture with 5:95 (a, c, e) and 10:90 (b, d, f) CO_2:N_2 at 0 °C (a, b), 25 °C (c, d) and 50 °C (e, f) measured on PAN-based CNFs carbonized at 600 °C. The measurements were performed at 5 bar overall pressure and with a flowrate of 100 mL/min. For a gas composition CO_2:N_2 of 5:95, 1% CO_2 and 19% N_2 (1.0 bar adsorptive pressure) in He were used and for a gas composition CO_2:N_2 of 10:90, 2% CO_2 and 18% N_2 (1.0 bar adsorptive pressure) in He were used.

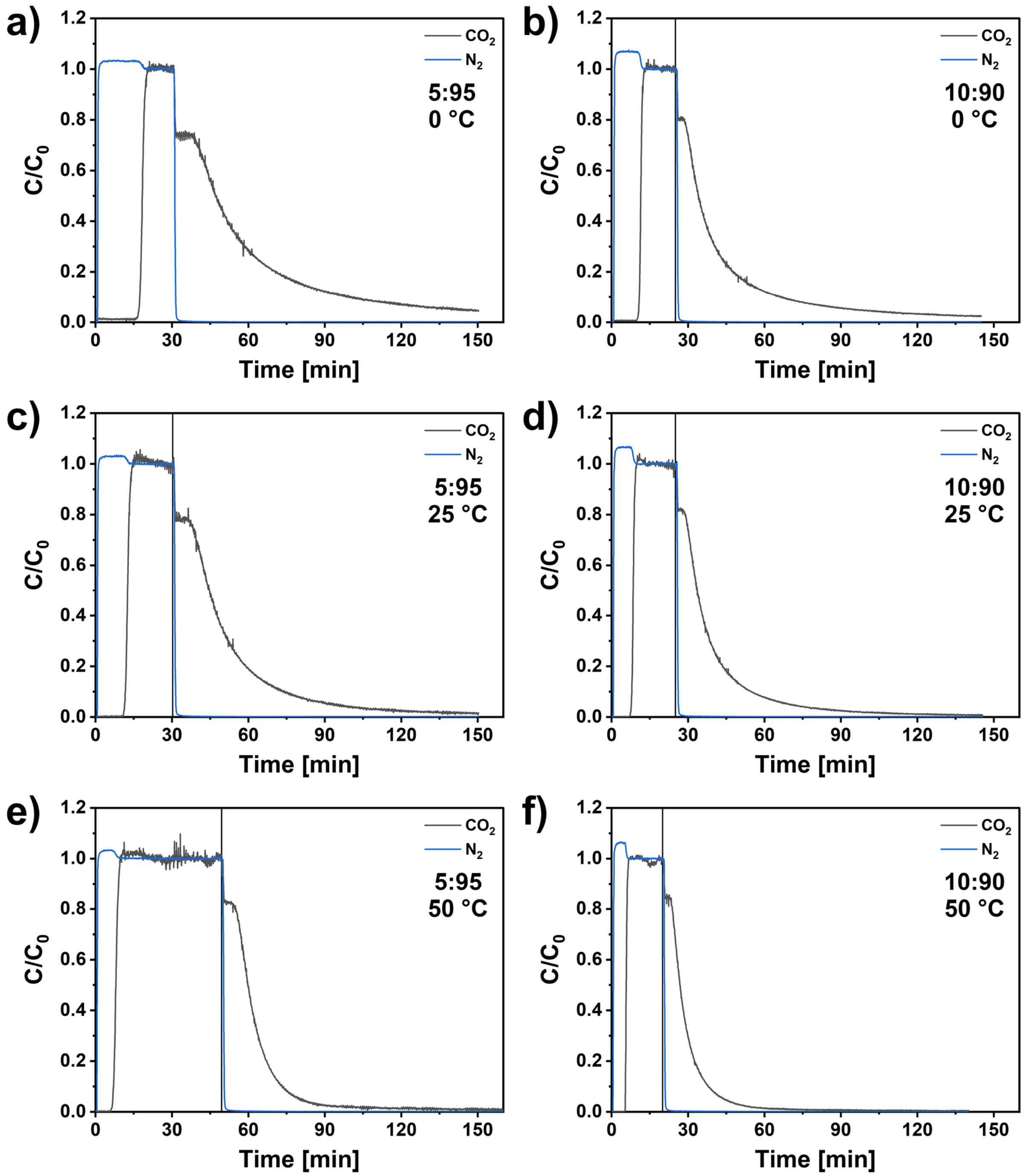

Figure S2-4: CO_2 and N_2 breakthrough curves of a gas mixture with 5:95 (a, c, e) and 10:90 (b, d, f) CO_2:N_2 at 0 °C (a, b), 25 °C (c, d) and 50 °C (e, f) measured on PAN-based CNFs carbonized at 600 °C. The measurements were performed at 5 bar overall pressure and with a flowrate of 100 mL/min. For a gas composition CO_2:N_2 of 5:95, 2% CO_2 and 38% N_2 (2.0 bar adsorptive pressure) in He were used and for a gas composition CO_2:N_2 of 10:90, 4% CO_2 and 36% N_2 (2.0 bar adsorptive pressure) in He were used.

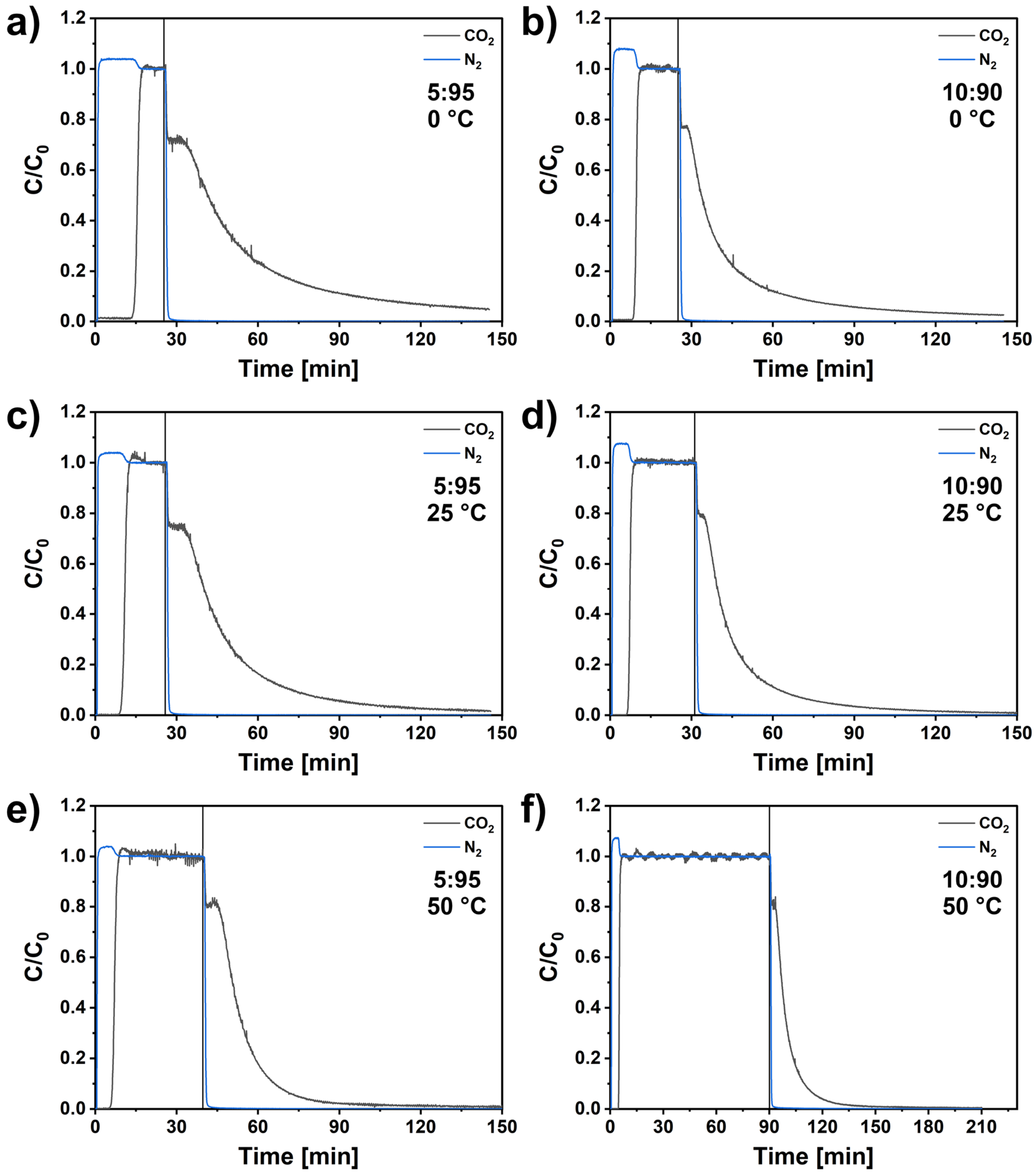

Figure S2-5: CO_2 and N_2 breakthrough curves of a gas mixture with 5:95 (a, c, e) and 10:90 (b, d, f) CO_2:N_2 at 0 °C (a, b), 25 °C (c, d) and 50 °C (e, f) measured on PAN-based CNFs carbonized at 600 °C. The measurements were performed at 5 bar overall pressure and with a flowrate of 100 mL/min. For a gas composition CO_2:N_2 of 5:95, 2.5% CO_2 and 47.5% N_2 (2.5 bar adsorptive pressure) in He were used and for a gas composition CO_2:N_2 of 10:90, 5% CO_2 and 45% N_2 (2.5 bar adsorptive pressure) in He were used.

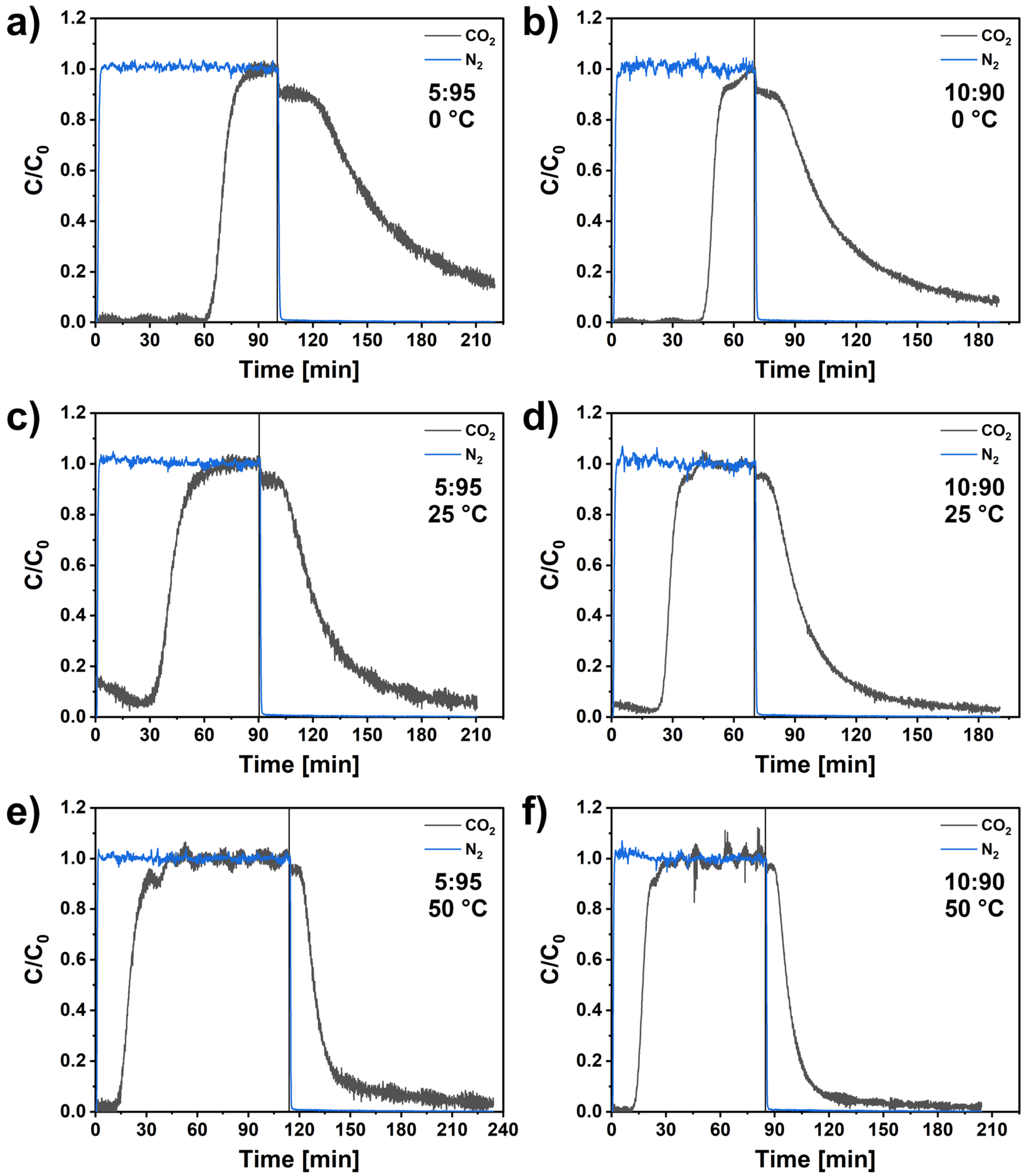

Figure S2-6: CO_2 and N_2 breakthrough curves of a gas mixture with 5:95 (a, c, e) and 10:90 (b, d, f) CO_2:N_2 at 0 °C (a, b), 25 °C (c, d) and 50 °C (e, f) measured on PAN-based CNFs carbonized at 700 °C. The measurements were performed at 5 bar overall pressure and with a flowrate of 100 mL/min. For a gas composition CO_2:N_2 of 5:95, 0.25% CO_2 and 4.75% N_2 (0.25 bar adsorptive pressure) in He were used and for a gas composition CO_2:N_2 of 10:90, 0.5% CO_2 and 4.5% N_2 (0.25 bar adsorptive pressure) in He were used.

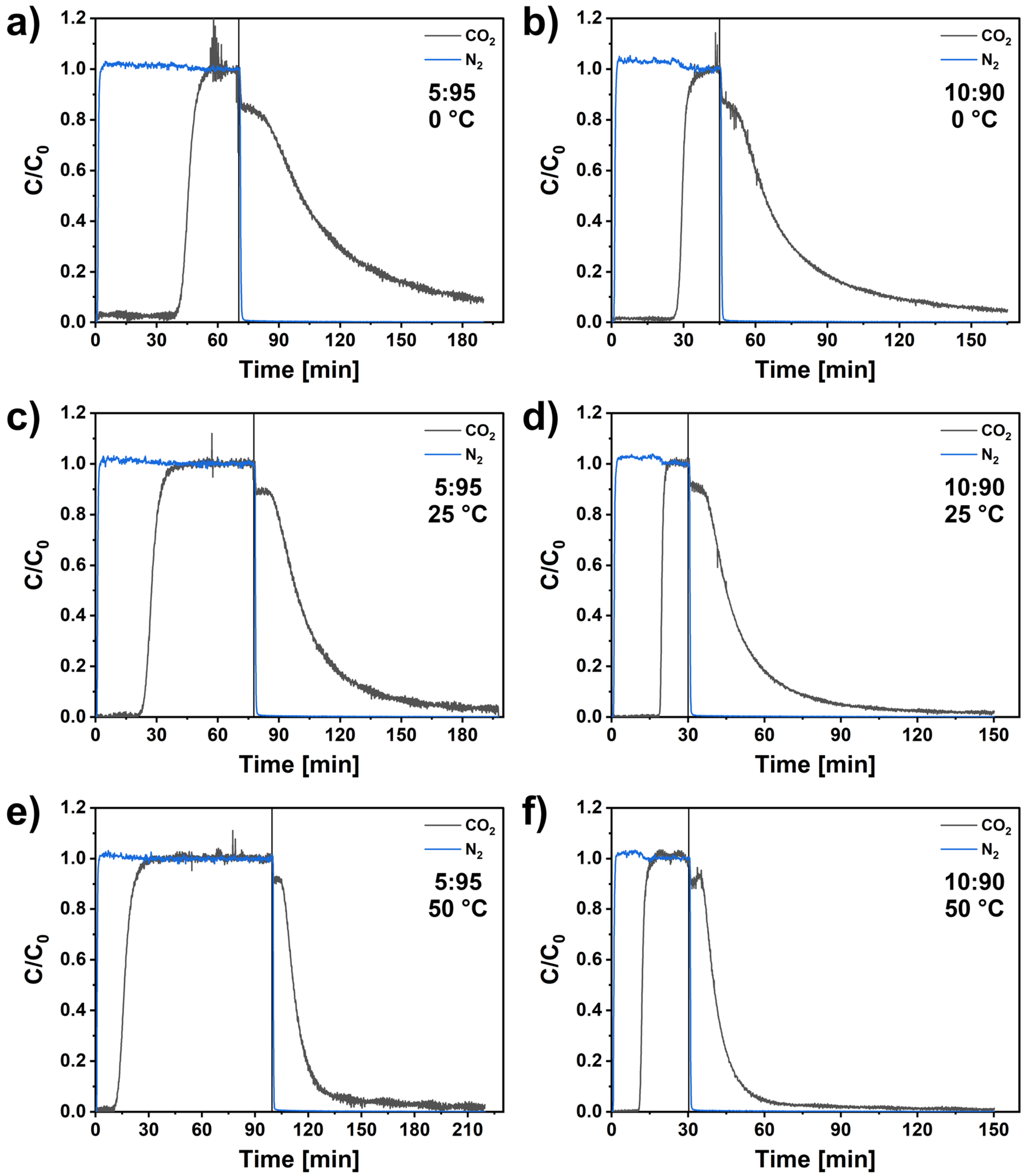

Figure S2-7: CO_2 and N_2 breakthrough curves of a gas mixture with 5:95 (a, c, e) and 10:90 (b, d, f) CO_2:N_2 at 0 °C (a, b), 25 °C (c, d) and 50 °C (e, f) measured on PAN-based CNFs carbonized at 700 °C. The measurements were performed at 5 bar overall pressure and with a flowrate of 100 mL/min. For a gas composition CO_2:N_2 of 5:95, 0.5% CO_2 and 9.5% N_2 (0.5 bar adsorptive pressure) in He were used and for a gas composition CO_2:N_2 of 10:90, 1% CO_2 and 9% N_2 (0.5 bar adsorptive pressure) in He were used.

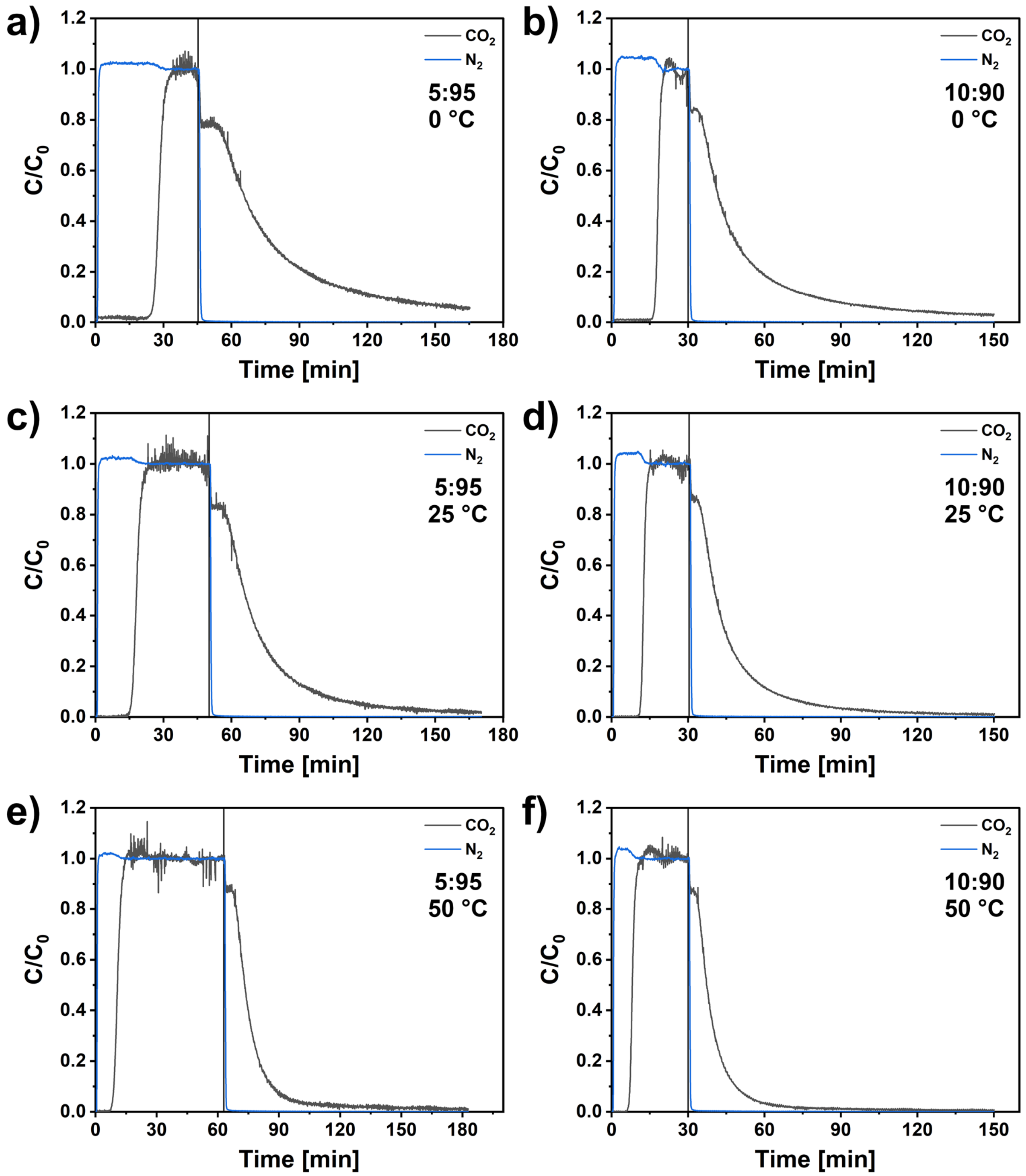

Figure S2-8: CO_2 and N_2 breakthrough curves of a gas mixture with 5:95 (a, c, e) and 10:90 (b, d, f) CO_2:N_2 at 0 °C (a, b), 25 °C (c, d) and 50 °C (e, f) measured on PAN-based CNFs carbonized at 700 °C. The measurements were performed at 5 bar overall pressure and with a flowrate of 100 mL/min. For a gas composition CO_2:N_2 of 5:95, 1% CO_2 and 19% N_2 (1.0 bar adsorptive pressure) in He were used and for a gas composition CO_2:N_2 of 10:90, 2% CO_2 and 18% N_2 (1.0 bar adsorptive pressure) in He were used.

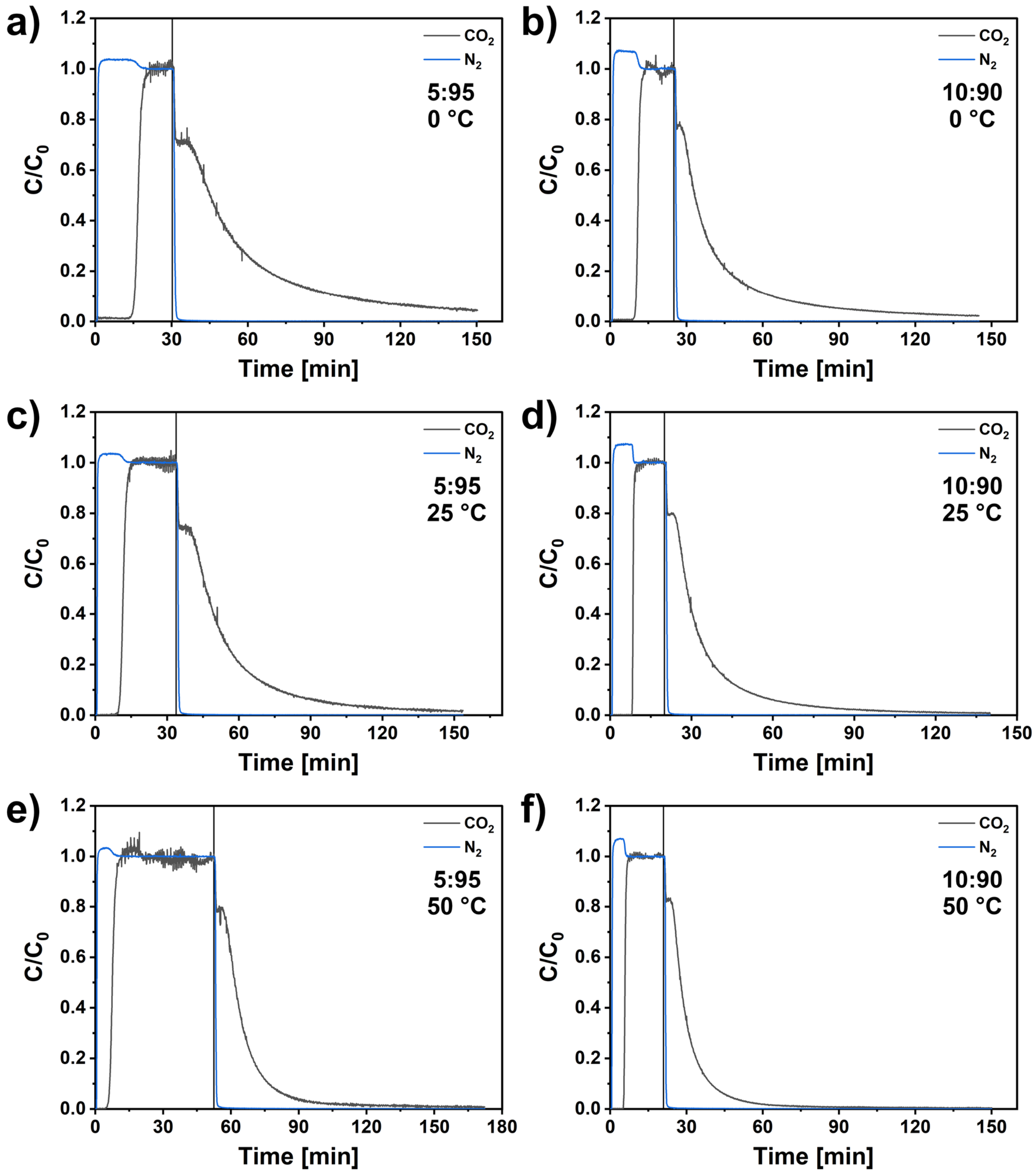

Figure S2-9: CO_2 and N_2 breakthrough curves of a gas mixture with 5:95 (a, c, e) and 10:90 (b, d, f) CO_2:N_2 at 0 °C (a, b), 25 °C (c, d) and 50 °C (e, f) measured on PAN-based CNFs carbonized at 700 °C. The measurements were performed at 5 bar overall pressure and with a flowrate of 100 mL/min. For a gas composition CO_2:N_2 of 5:95, 2% CO_2 and 38% N_2 (2.0 bar adsorptive pressure) in He were used and for a gas composition CO_2:N_2 of 10:90, 4% CO_2 and 36% N_2 (2.0 bar adsorptive pressure) in He were used.

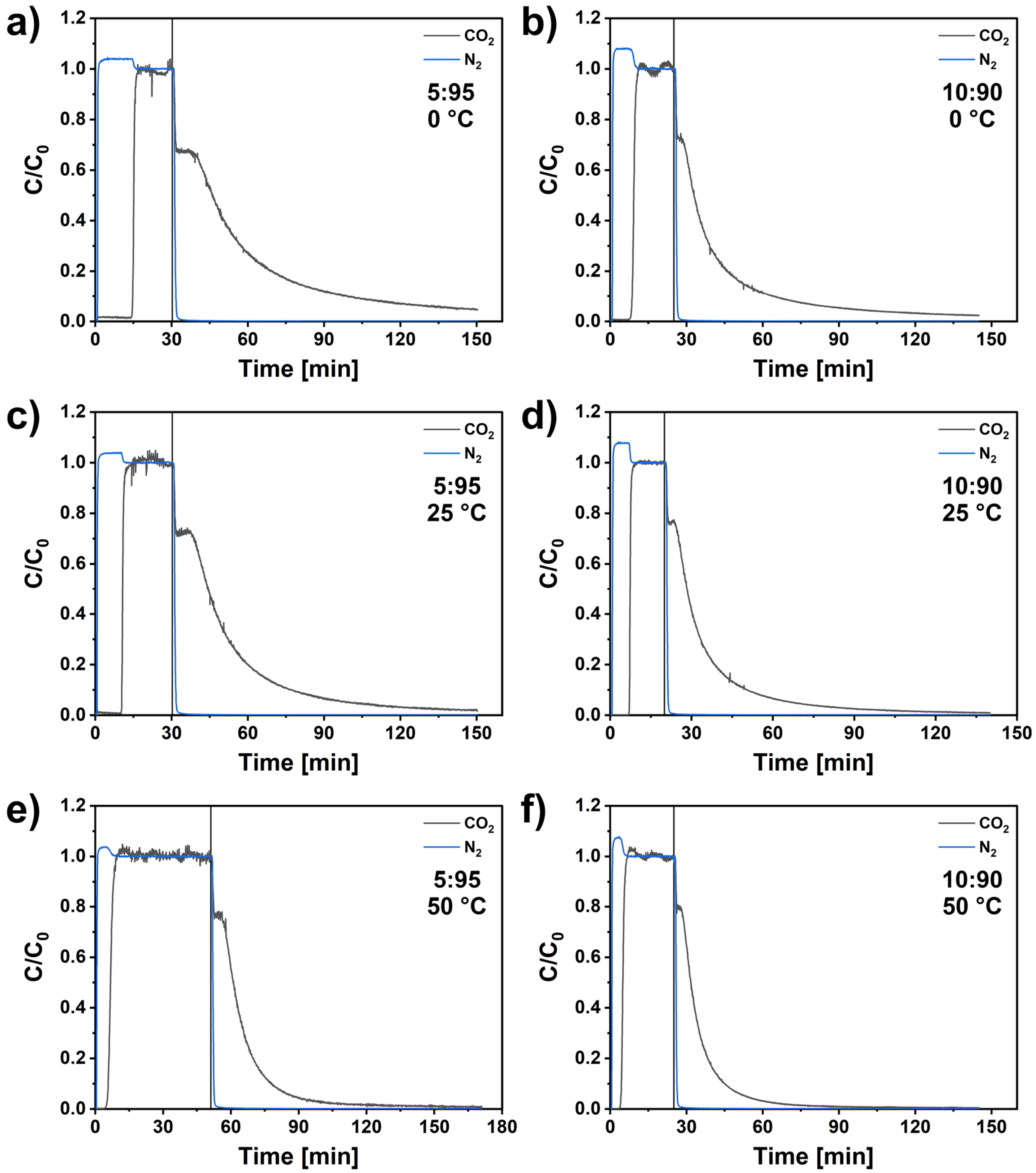

Figure S2-10: CO_2 and N_2 breakthrough curves of a gas mixture with 5:95 (a, c, e) and 10:90 (b, d, f) CO_2:N_2 at 0 °C (a, b), 25 °C (c, d) and 50 °C (e, f) measured on PAN-based CNFs carbonized at 700 °C. The measurements were performed at 5 bar overall pressure and with a flowrate of 100 mL/min. For a gas composition CO_2:N_2 of 5:95, 2.5% CO_2 and 47.5% N_2 (2.5 bar adsorptive pressure) in He were used and for a gas composition CO_2:N_2 of 10:90, 5% CO_2 and 45% N_2 (2.5 bar adsorptive pressure) in He were used.

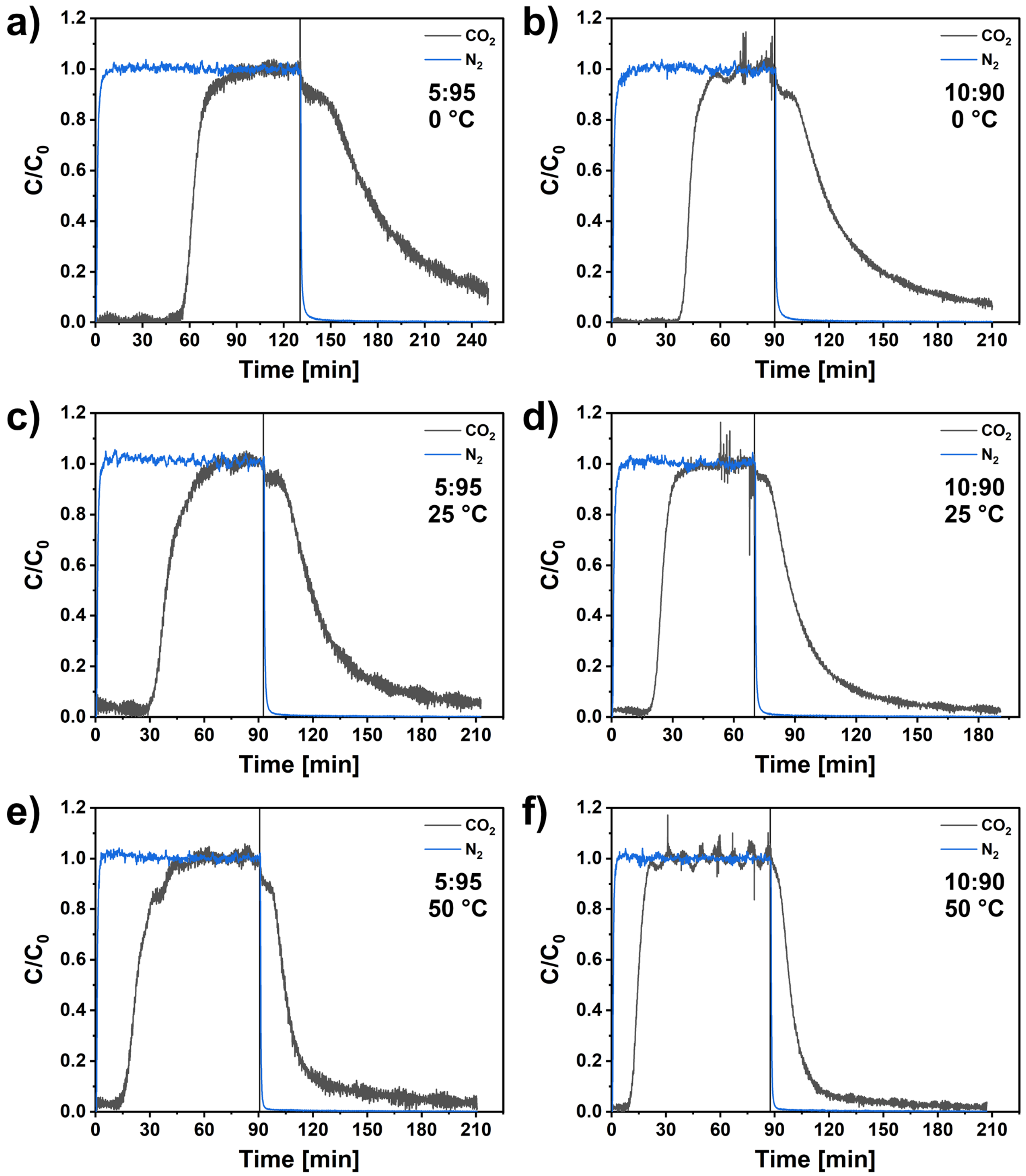

Figure S2-11: CO_2 and N_2 breakthrough curves of a gas mixture with 5:95 (a, c, e) and 10:90 (b, d, f) CO_2:N_2 at 0 °C (a, b), 25 °C (c, d) and 50 °C (e, f) measured on PAN-based CNFs carbonized at 800 °C. The measurements were performed at 5 bar overall pressure and with a flowrate of 100 mL/min. For a gas composition CO_2:N_2 of 5:95, 0.25% CO_2 and 4.75% N_2 (0.25 bar adsorptive pressure) in He were used and for a gas composition CO_2:N_2 of 10:90, 0.5% CO_2 and 4.5% N_2 (0.25 bar adsorptive pressure) in He were used.

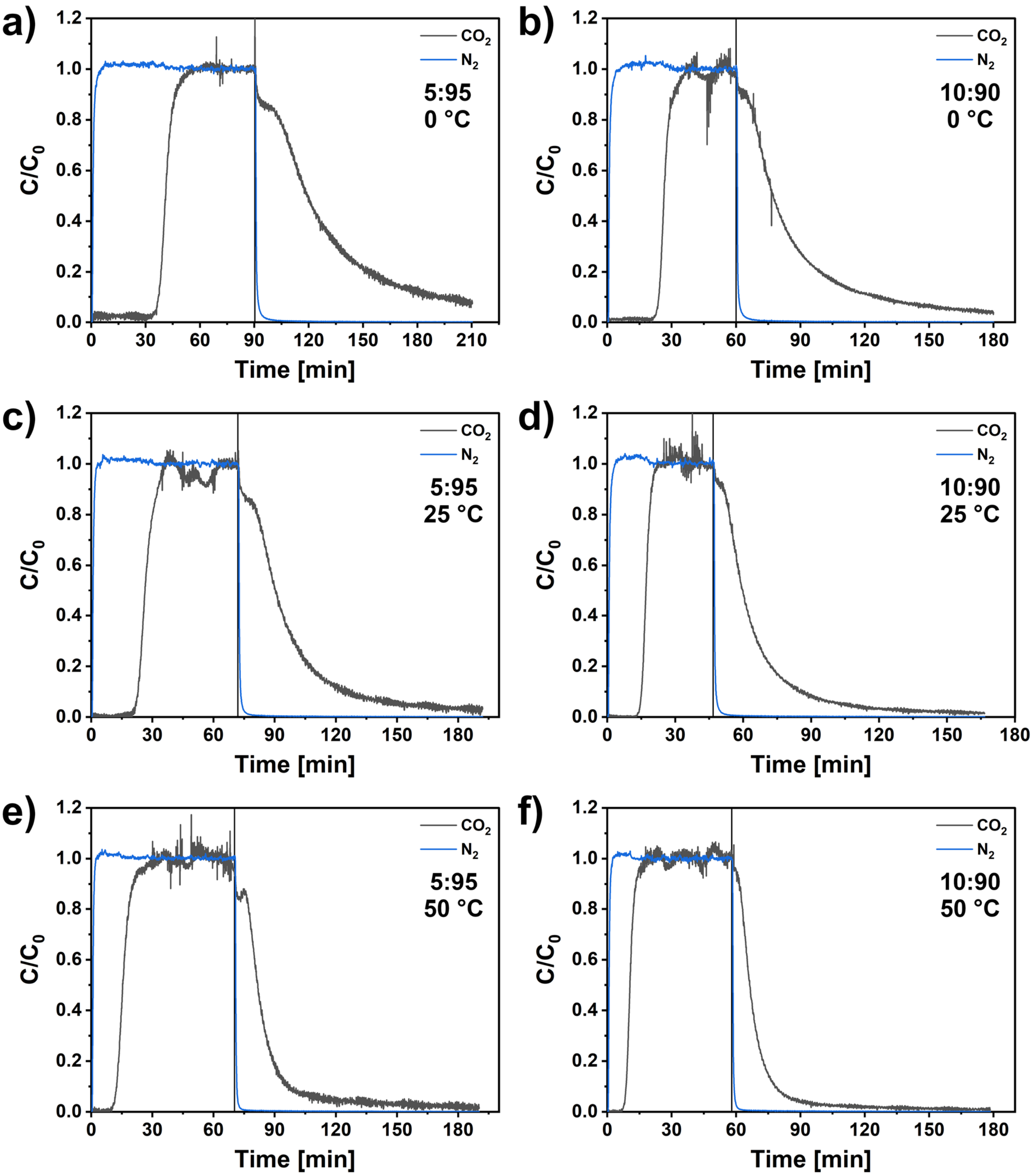

Figure S2-12: CO_2 and N_2 breakthrough curves of a gas mixture with 5:95 (a, c, e) and 10:90 (b, d, f) CO_2:N_2 at 0 °C (a, b), 25 °C (c, d) and 50 °C (e, f) measured on PAN-based CNFs carbonized at 800 °C. The measurements were performed at 5 bar overall pressure and with a flowrate of 100 mL/min. For a gas composition CO_2:N_2 of 5:95, 0.5% CO_2 and 9.5% N_2 (0.5 bar adsorptive pressure) in He were used and for a gas composition CO_2:N_2 of 10:90, 1% CO_2 and 9% N_2 (0.5 bar adsorptive pressure) in He were used.

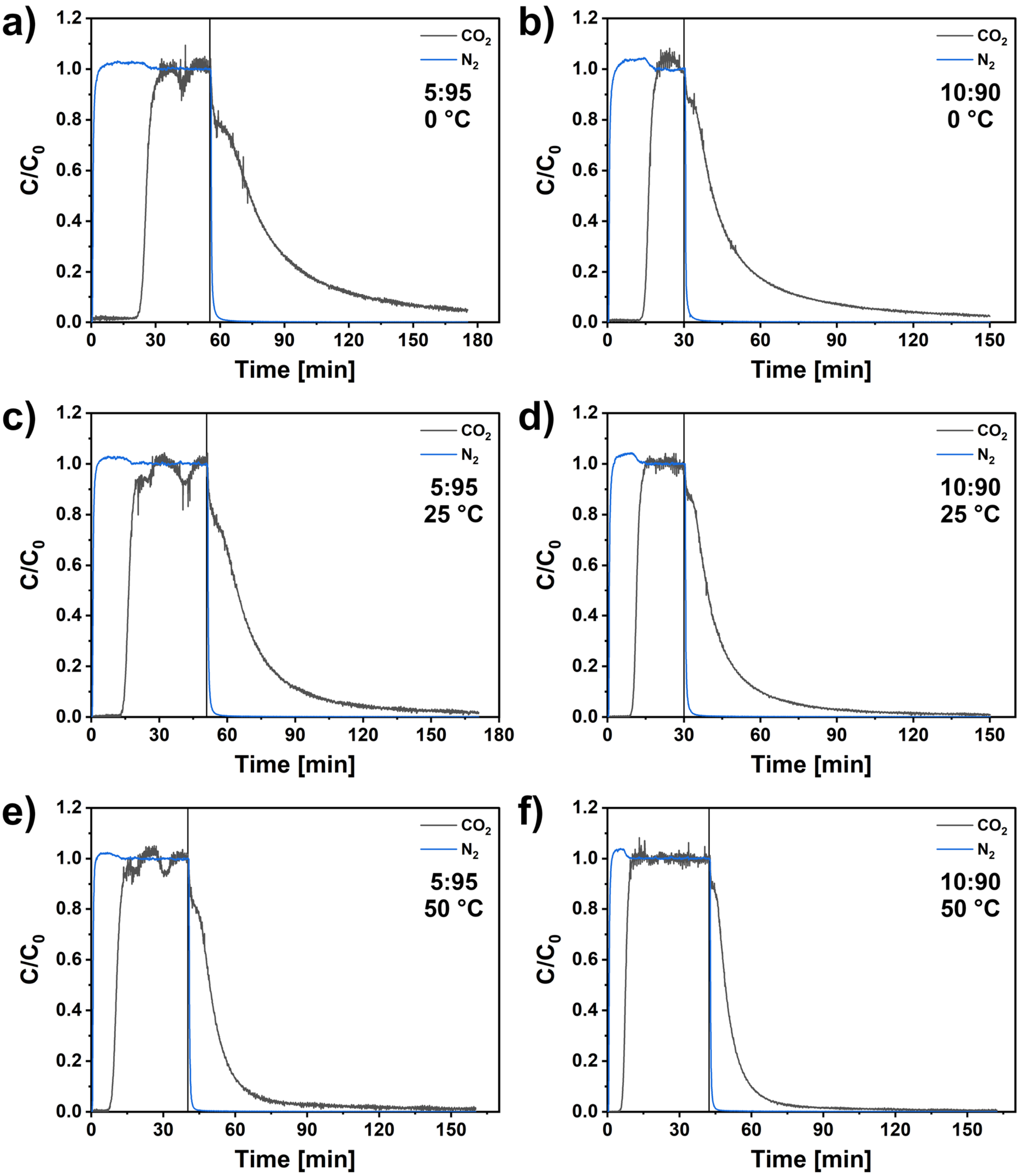

Figure S2-13: CO_2 and N_2 breakthrough curves of a gas mixture with 5:95 (a, c, e) and 10:90 (b, d, f) CO_2:N_2 at 0 °C (a, b), 25 °C (c, d) and 50 °C (e, f) measured on PAN-based CNFs carbonized at 800 °C. The measurements were performed at 5 bar overall pressure and with a flowrate of 100 mL/min. For a gas composition CO_2:N_2 of 5:95, 1% CO_2 and 19% N_2 (1.0 bar adsorptive pressure) in He were used and for a gas composition CO_2:N_2 of 10:90, 2% CO_2 and 18% N_2 (1.0 bar adsorptive pressure) in He were used.

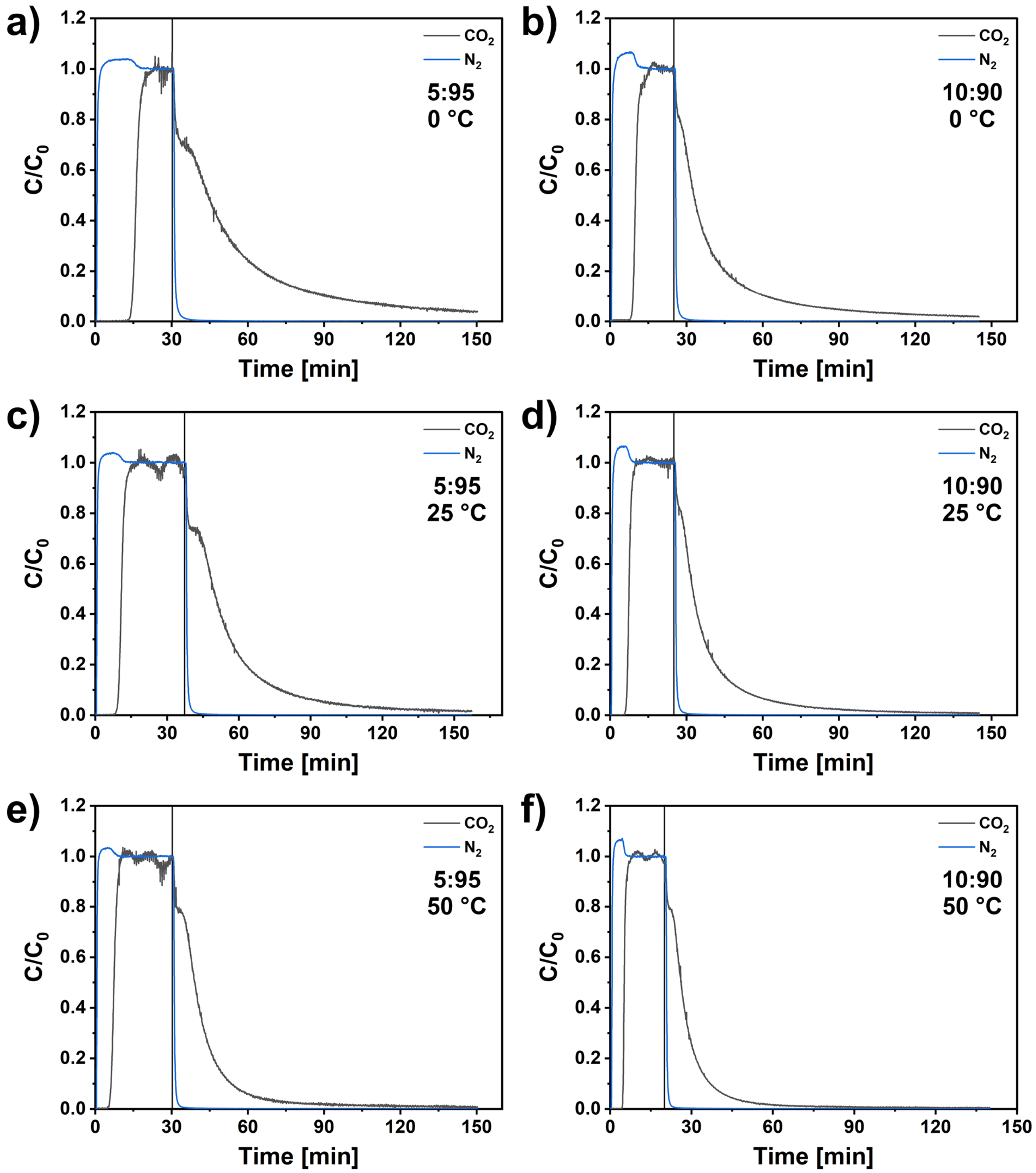

Figure S2-14: CO_2 and N_2 breakthrough curves of a gas mixture with 5:95 (a, c, e) and 10:90 (b, d, f) CO_2:N_2 at 0 °C (a, b), 25 °C (c, d) and 50 °C (e, f) measured on PAN-based CNFs carbonized at 800 °C. The measurements were performed at 5 bar overall pressure and with a flowrate of 100 mL/min. For a gas composition CO_2:N_2 of 5:95, 2% CO_2 and 38% N_2 (2.0 bar adsorptive pressure) in He were used and for a gas composition CO_2:N_2 of 10:90, 4% CO_2 and 36% N_2 (2.0 bar adsorptive pressure) in He were used.

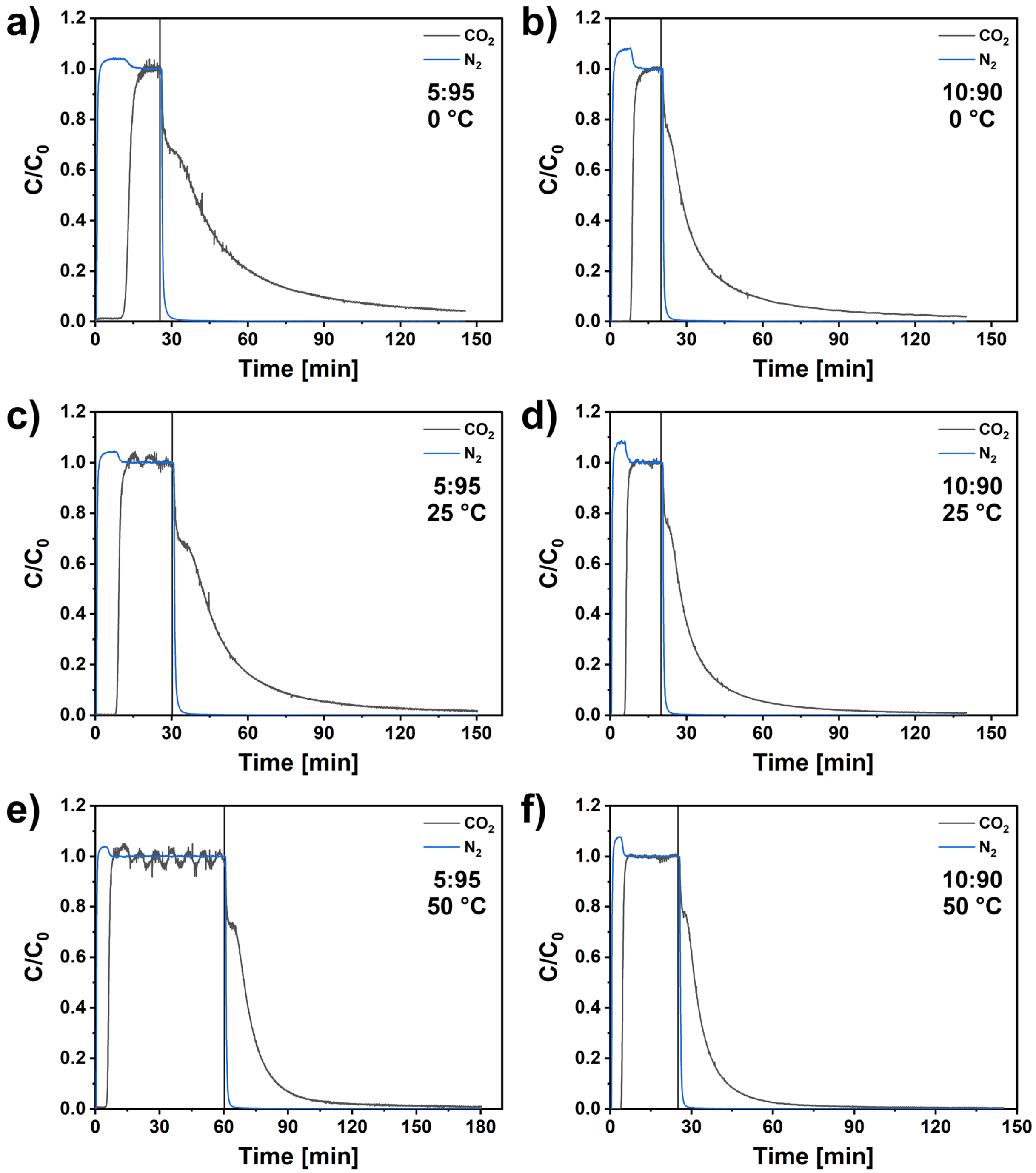

Figure S2-15: CO_2 and N_2 breakthrough curves of a gas mixture with 5:95 (a, c, e) and 10:90 (b, d, f) CO_2:N_2 at 0 °C (a, b), 25 °C (c, d) and 50 °C (e, f) measured on PAN-based CNFs carbonized at 800 °C. The measurements were performed at 5 bar overall pressure and with a flowrate of 100 mL/min. For a gas composition CO_2:N_2 of 5:95, 2.5% CO_2 and 47.5% N_2 (2.5 bar adsorptive pressure) in He were used and for a gas composition CO_2:N_2 of 10:90, 5% CO_2 and 45% N_2 (2.5 bar adsorptive pressure) in He were used.

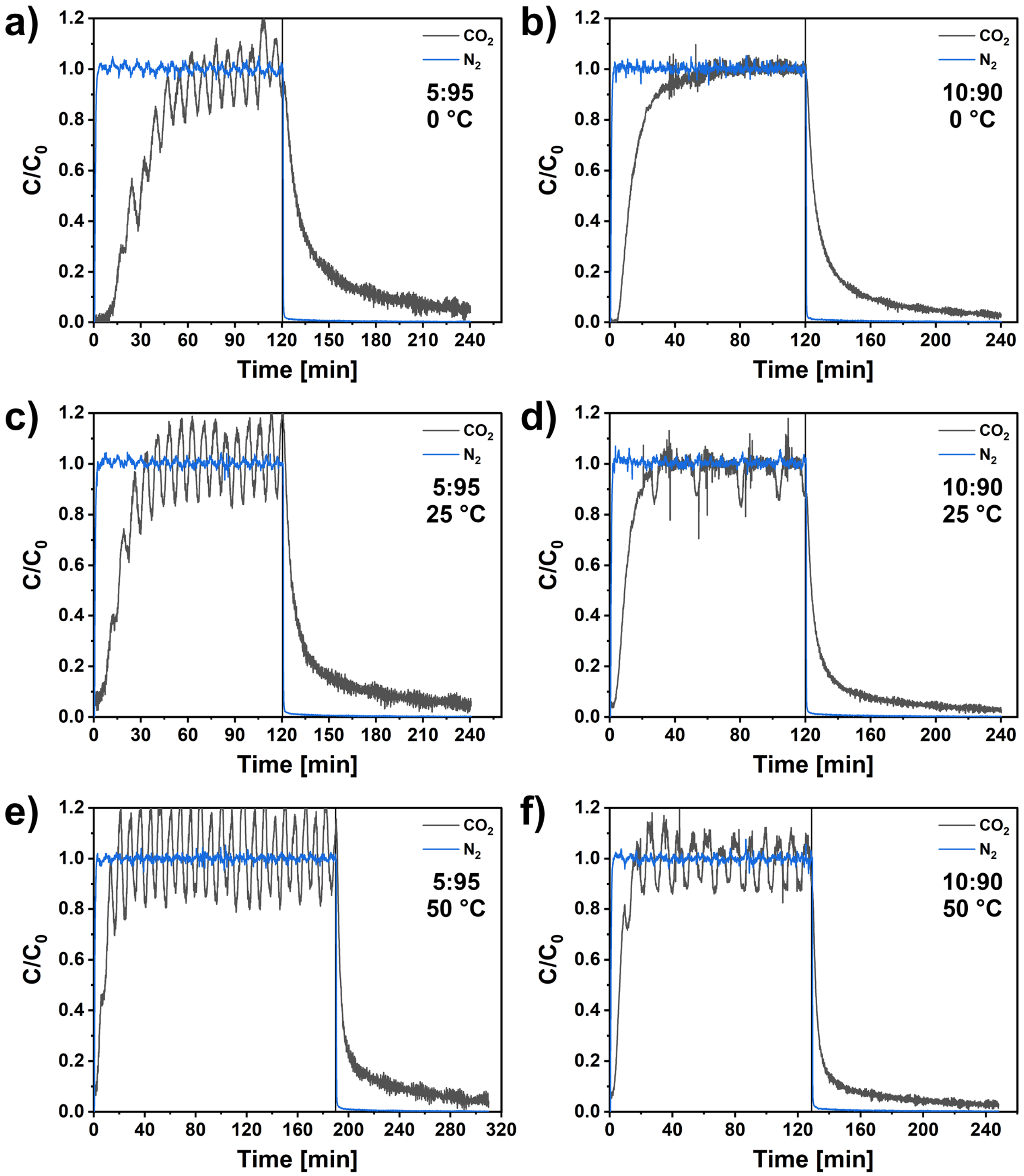

Figure S2-16: CO_2 and N_2 breakthrough curves of a gas mixture with 5:95 (a, c, e) and 10:90 (b, d, f) CO_2:N_2 at 0 °C (a, b), 25 °C (c, d) and 50 °C (e, f) measured on PAN-based CNFs carbonized at 900 °C. The measurements were performed at 5 bar overall pressure and with a flowrate of 100 mL/min. For a gas composition CO_2:N_2 of 5:95, 0.25% CO_2 and 4.75% N_2 (0.25 bar adsorptive pressure) in He were used and for a gas composition CO_2:N_2 of 10:90, 0.5% CO_2 and 4.5% N_2 (0.25 bar adsorptive pressure) in He were used.

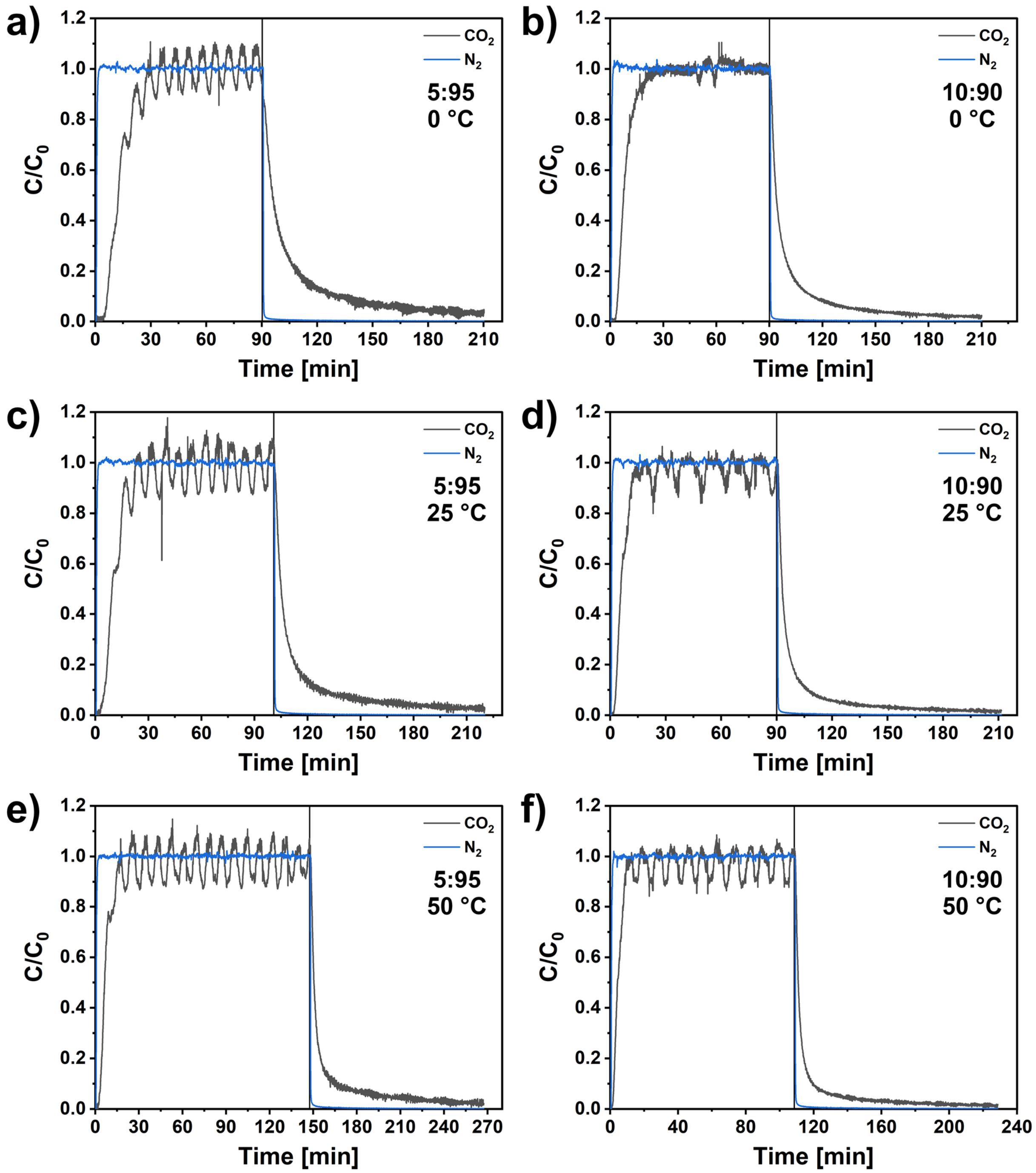

Figure S2-17: CO_2 and N_2 breakthrough curves of a gas mixture with 5:95 (a, c, e) and 10:90 (b, d, f) CO_2:N_2 at 0 °C (a, b), 25 °C (c, d) and 50 °C (e, f) measured on PAN-based CNFs carbonized at 900 °C. The measurements were performed at 5 bar overall pressure and with a flowrate of 100 mL/min. For a gas composition CO_2:N_2 of 5:95, 0.5% CO_2 and 9.5% N_2 (0.5 bar adsorptive pressure) in He were used and for a gas composition CO_2:N_2 of 10:90, 1% CO_2 and 9% N_2 (0.5 bar adsorptive pressure) in He were used.

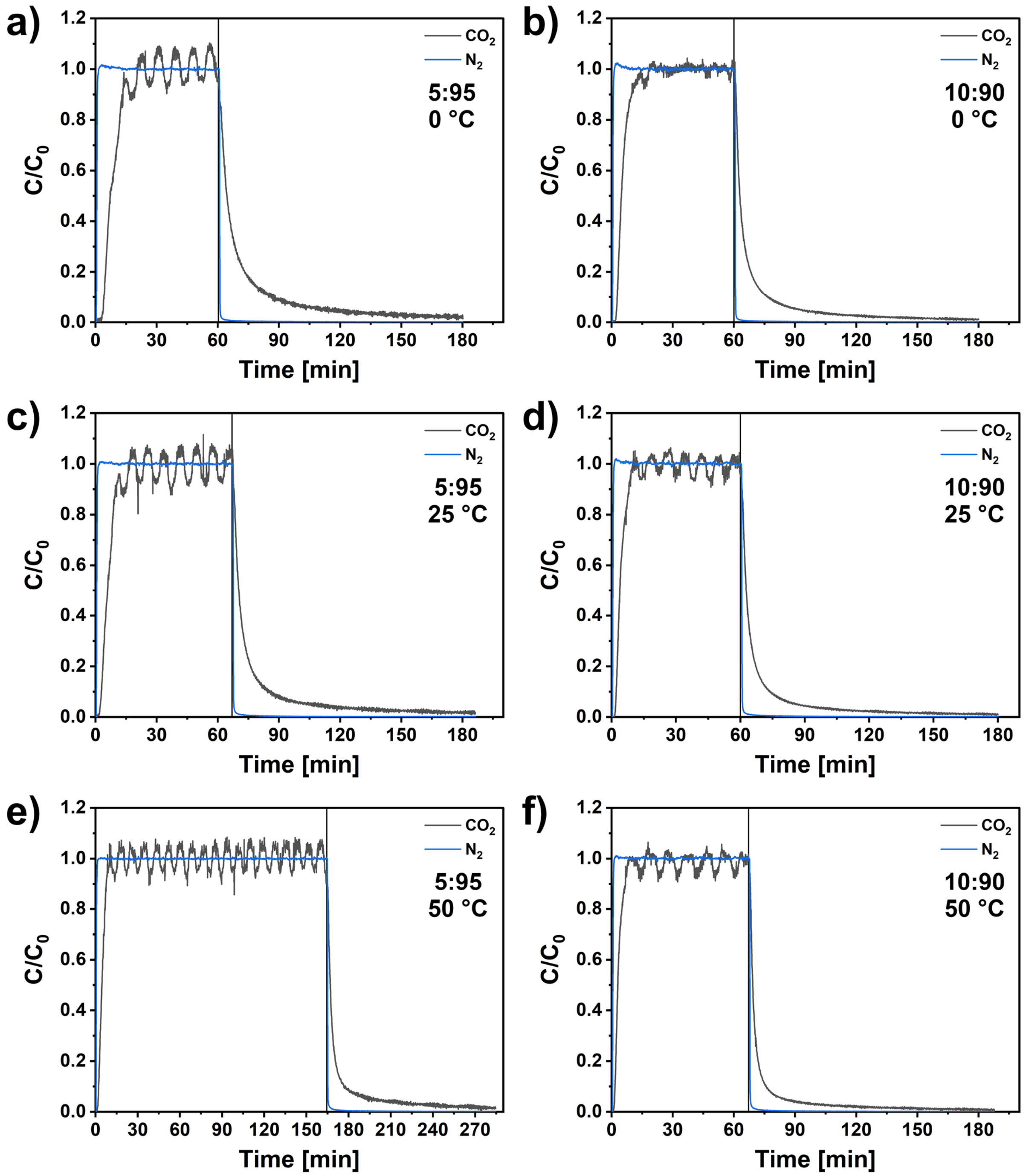

Figure S2-18: CO_2 and N_2 breakthrough curves of a gas mixture with 5:95 (a, c, e) and 10:90 (b, d, f) CO_2:N_2 at 0 °C (a, b), 25 °C (c, d) and 50 °C (e, f) measured on PAN-based CNFs carbonized at 900 °C. The measurements were performed at 5 bar overall pressure and with a flowrate of 100 mL/min. For a gas composition CO_2:N_2 of 5:95, 1% CO_2 and 19% N_2 (1.0 bar adsorptive pressure) in He were used and for a gas composition CO_2:N_2 of 10:90, 2% CO_2 and 18% N_2 (1.0 bar adsorptive pressure) in He were used.

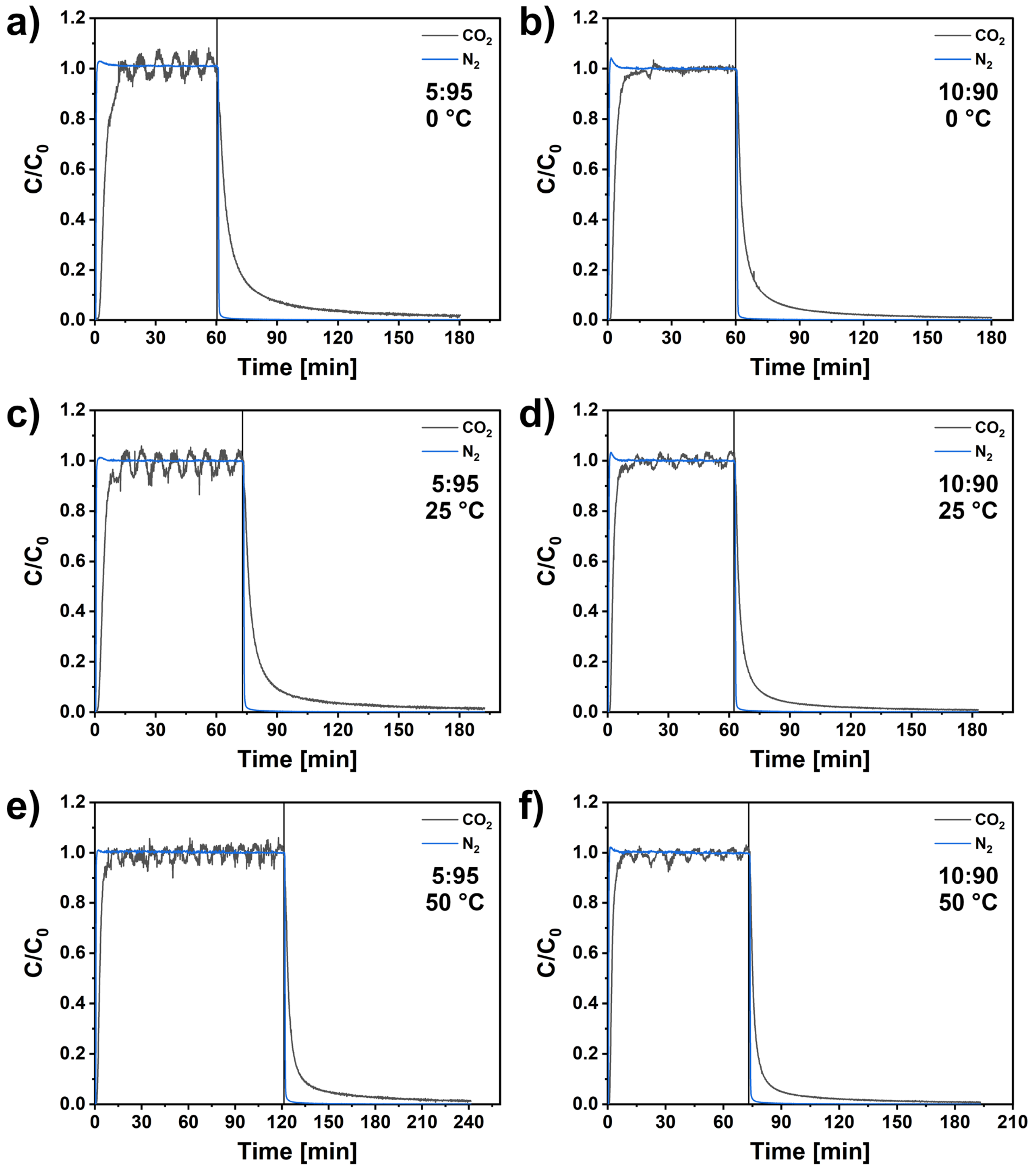

Figure S2-19: CO_2 and N_2 breakthrough curves of a gas mixture with 5:95 (a, c, e) and 10:90 (b, d, f) CO_2:N_2 at 0 °C (a, b), 25 °C (c, d) and 50 °C (e, f) measured on PAN-based CNFs carbonized at 900 °C. The measurements were performed at 5 bar overall pressure and with a flowrate of 100 mL/min. For a gas composition CO_2:N_2 of 5:95, 2% CO_2 and 38% N_2 (2.0 bar adsorptive pressure) in He were used and for a gas composition CO_2:N_2 of 10:90, 4% CO_2 and 36% N_2 (2.0 bar adsorptive pressure) in He were used.

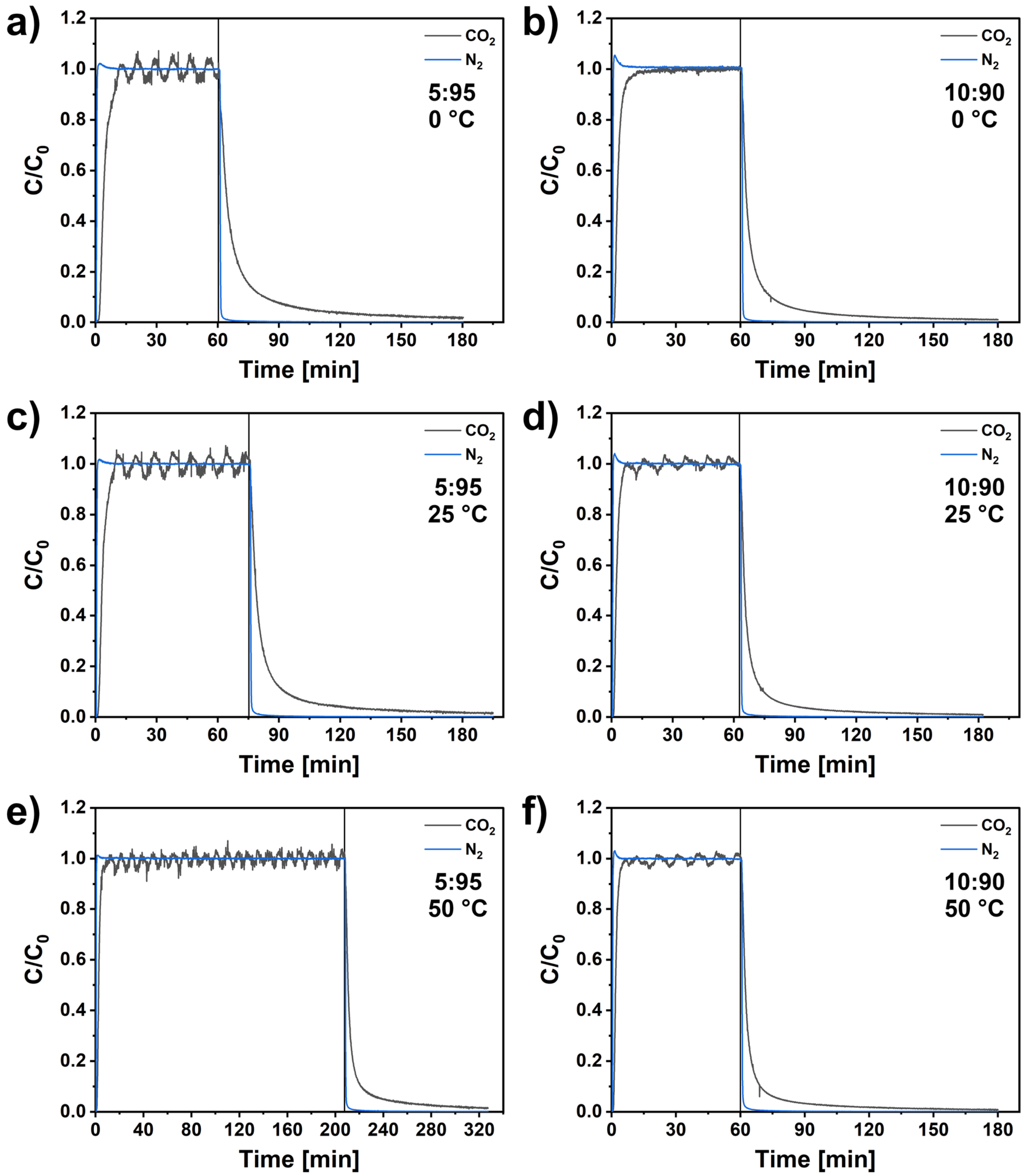

Figure S2-20: CO_2 and N_2 breakthrough curves of a gas mixture with 5:95 (a, c, e) and 10:90 (b, d, f) CO_2:N_2 at 0 °C (a, b), 25 °C (c, d) and 50 °C (e, f) measured on PAN-based CNFs carbonized at 900 °C. The measurements were performed at 5 bar overall pressure and with a flowrate of 100 mL/min. For a gas composition CO_2:N_2 of 5:95, 2.5% CO_2 and 47.5% N_2 (2.5 bar adsorptive pressure) in He were used and for a gas composition CO_2:N_2 of 10:90, 5% CO_2 and 45% N_2 (2.5 bar adsorptive pressure) in He were used.

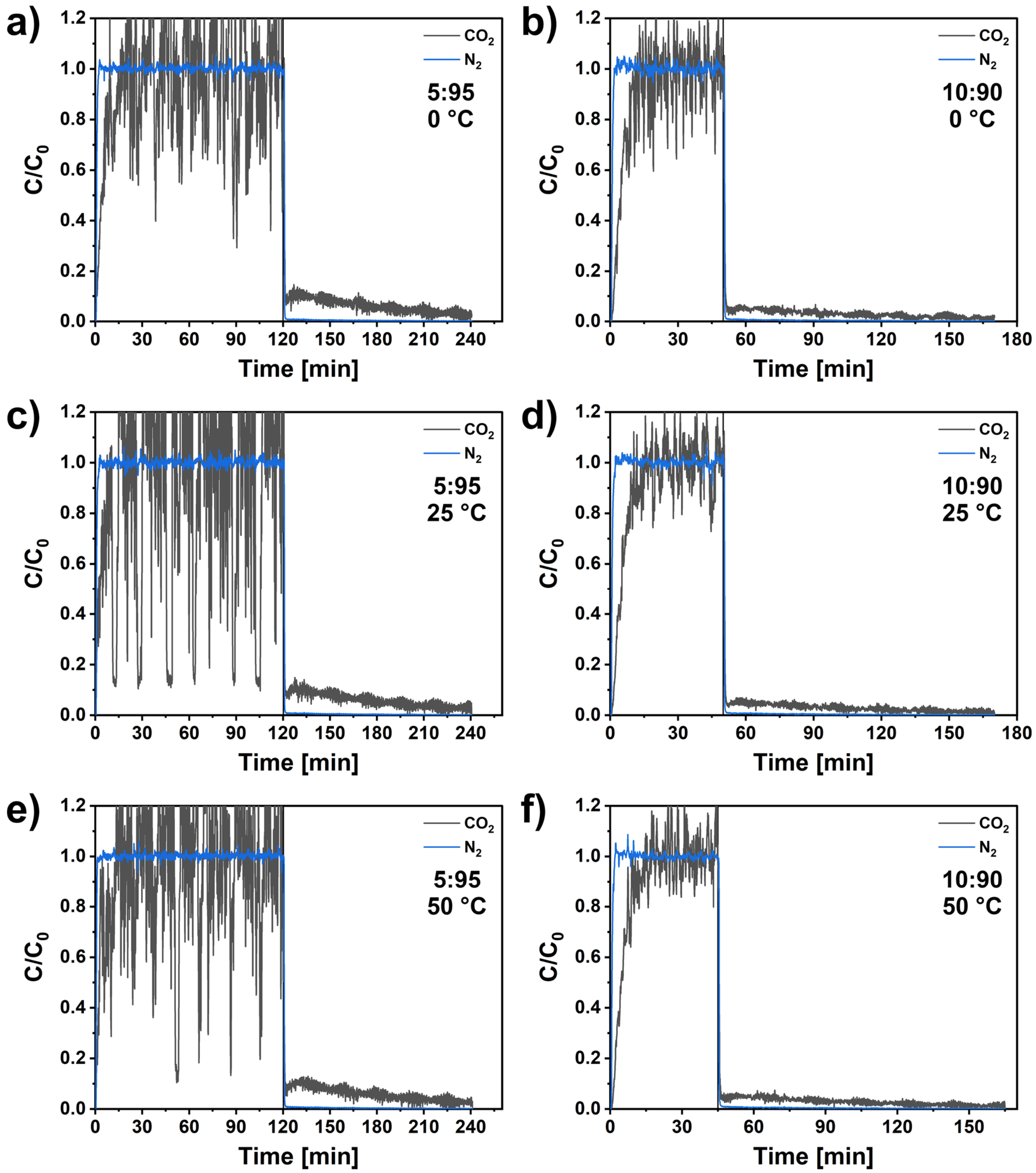

Figure S2-21: CO_2 and N_2 breakthrough curves of a gas mixture with 5:95 (a, c, e) and 10:90 (b, d, f) CO_2:N_2 at 0 °C (a, b), 25 °C (c, d) and 50 °C (e, f) measured with glass beads as filling material. The measurements were performed at 5 bar overall pressure and with a flowrate of 100 mL/min. For a gas composition CO_2:N_2 of 5:95, 0.25% CO_2 and 4.75% N_2 (0.25 bar adsorptive pressure) in He were used and for a gas composition CO_2:N_2 of 10:90, 0.5% CO_2 and 4.5% N_2 (0.25 bar adsorptive pressure) in He were used.

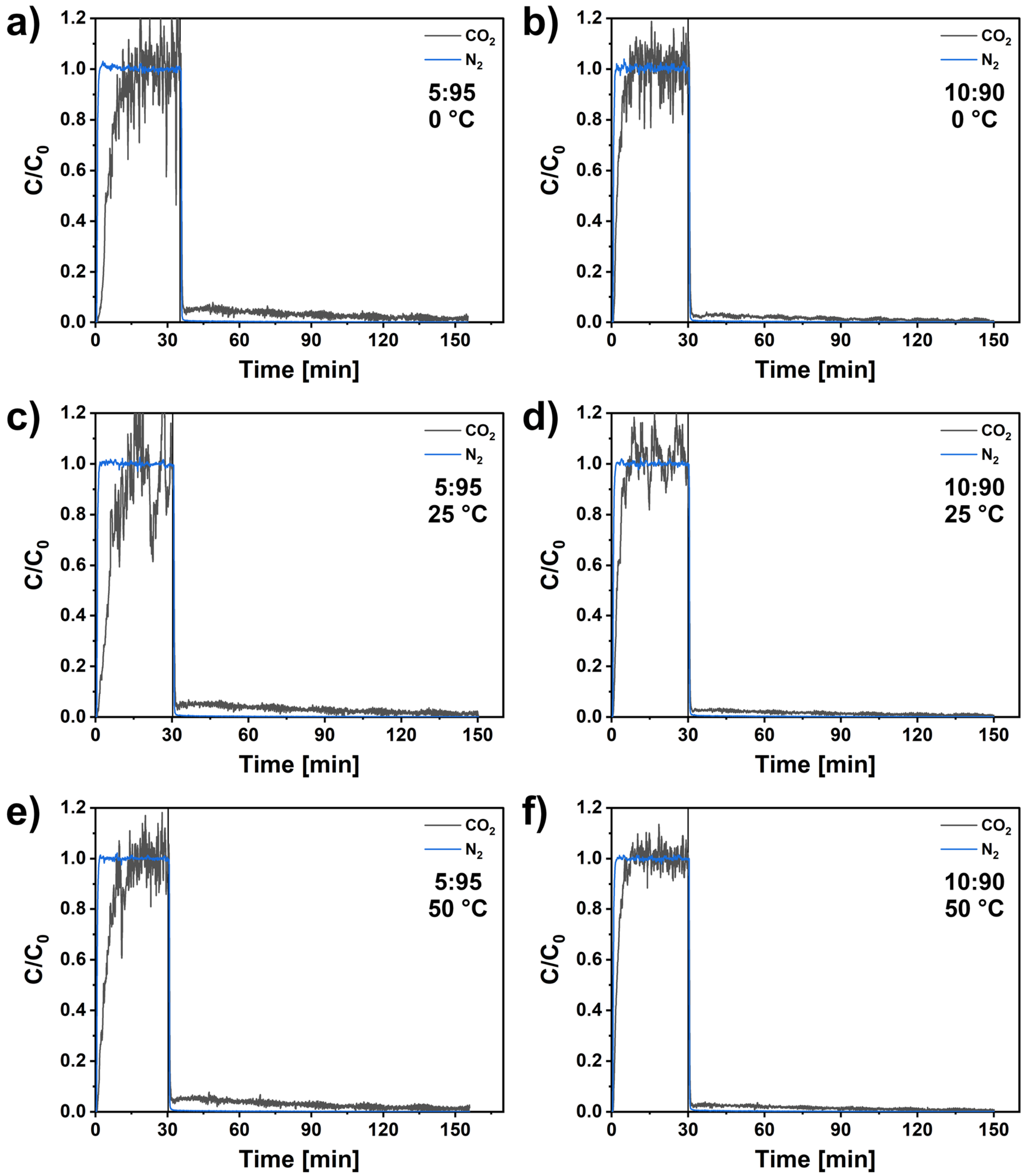

Figure S2- 22: CO_2 and N_2 breakthrough curves of a gas mixture with 5:95 (a, c, e) and 10:90 (b, d, f) CO_2:N_2 at 0 °C (a, b), 25 °C (c, d) and 50 °C (e, f) measured with glass beads as filling material. The measurements were performed at 5 bar overall pressure and with a flowrate of 100 mL/min. For a gas composition CO_2:N_2 of 5:95, 0.5% CO_2 and 9.5% N_2 (0.5 bar adsorptive pressure) in He were used and for a gas composition CO_2:N_2 of 10:90, 1% CO_2 and 9% N_2 (0.5 bar adsorptive pressure) in He were used.

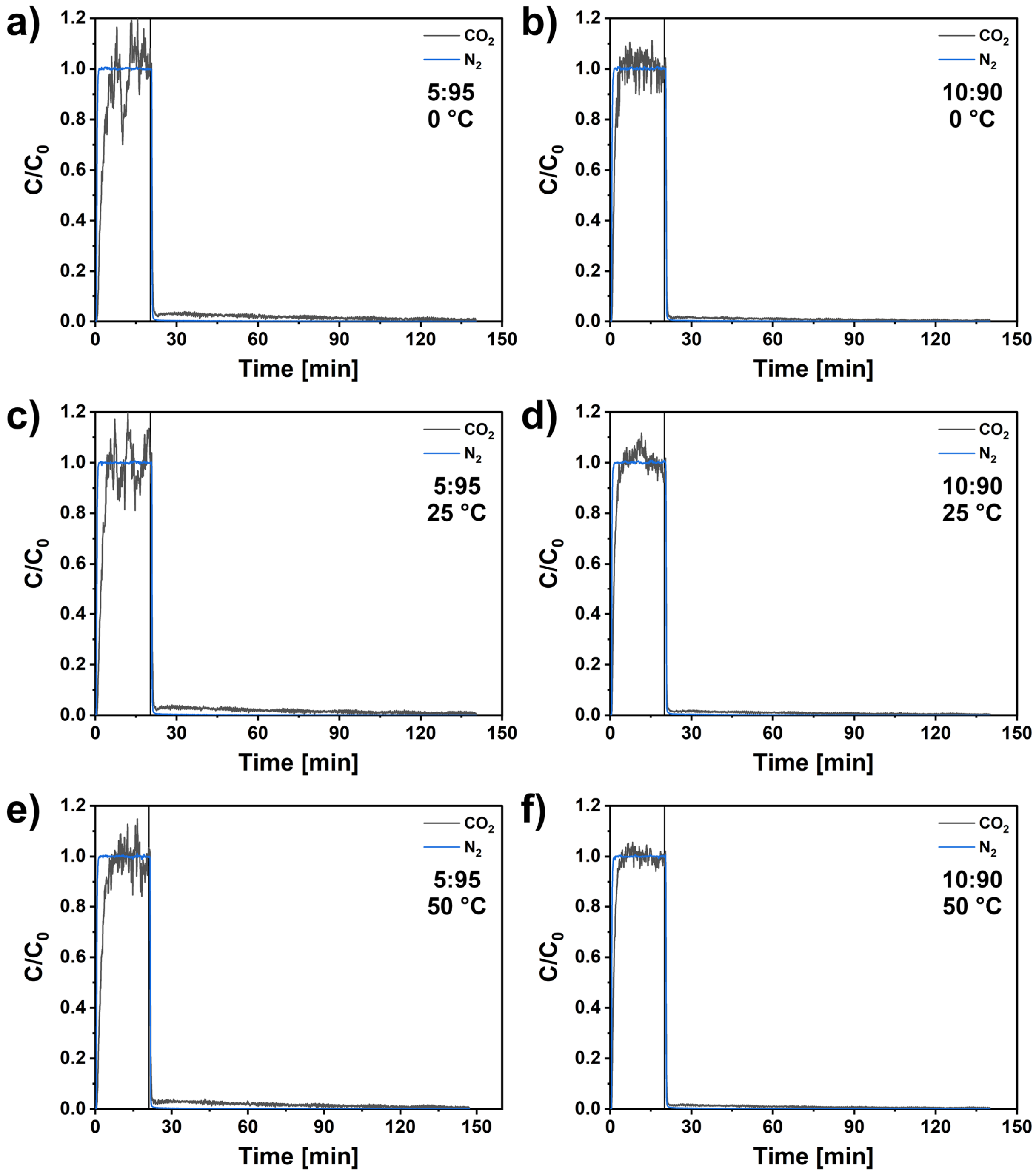

Figure S2-23: CO_2 and N_2 breakthrough curves of a gas mixture with 5:95 (a, c, e) and 10:90 (b, d, f) CO_2:N_2 at 0 °C (a, b), 25 °C (c, d) and 50 °C (e, f) measured with glass beads as filling material. The measurements were performed at 5 bar overall pressure and with a flowrate of 100 mL/min. For a gas composition CO_2:N_2 of 5:95, 1% CO_2 and 19% N_2 (1.0 bar adsorptive pressure) in He were used and for a gas composition CO_2:N_2 of 10:90, 2% CO_2 and 18% N_2 (1.0 bar adsorptive pressure) in He were used.

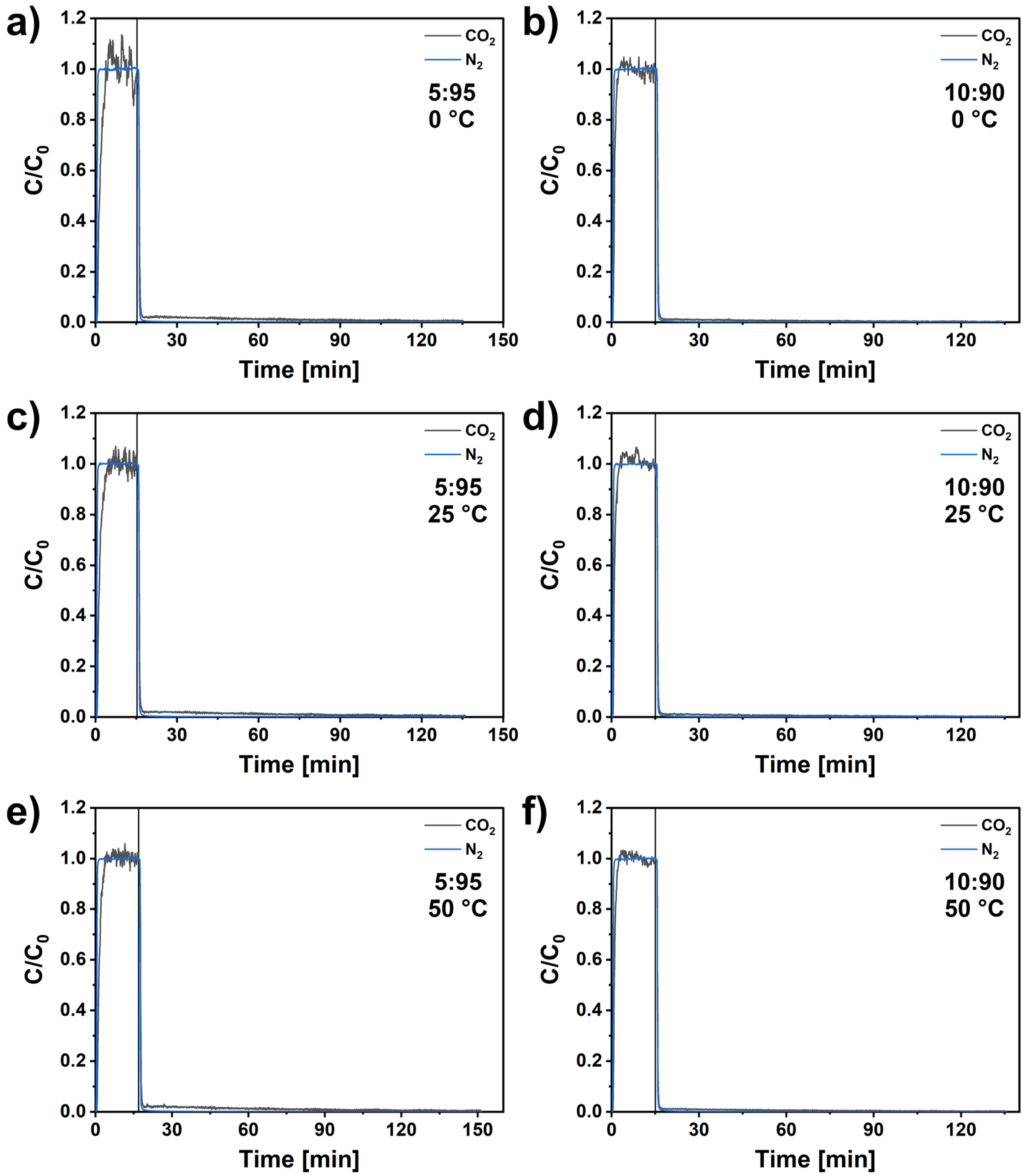

Figure S2-24: CO_2 and N_2 breakthrough curves of a gas mixture with 5:95 (a, c, e) and 10:90 (b, d, f) CO_2:N_2 at 0 °C (a, b), 25 °C (c, d) and 50 °C (e, f) measured with glass beads as filling material. The measurements were performed at 5 bar overall pressure and with a flowrate of 100 mL/min. For a gas composition CO_2:N_2 of 5:95, 2% CO_2 and 38% N_2 (2.0 bar adsorptive pressure) in He were used and for a gas composition CO_2:N_2 of 10:90, 4% CO_2 and 36% N_2 (2.0 bar adsorptive pressure) in He were used.

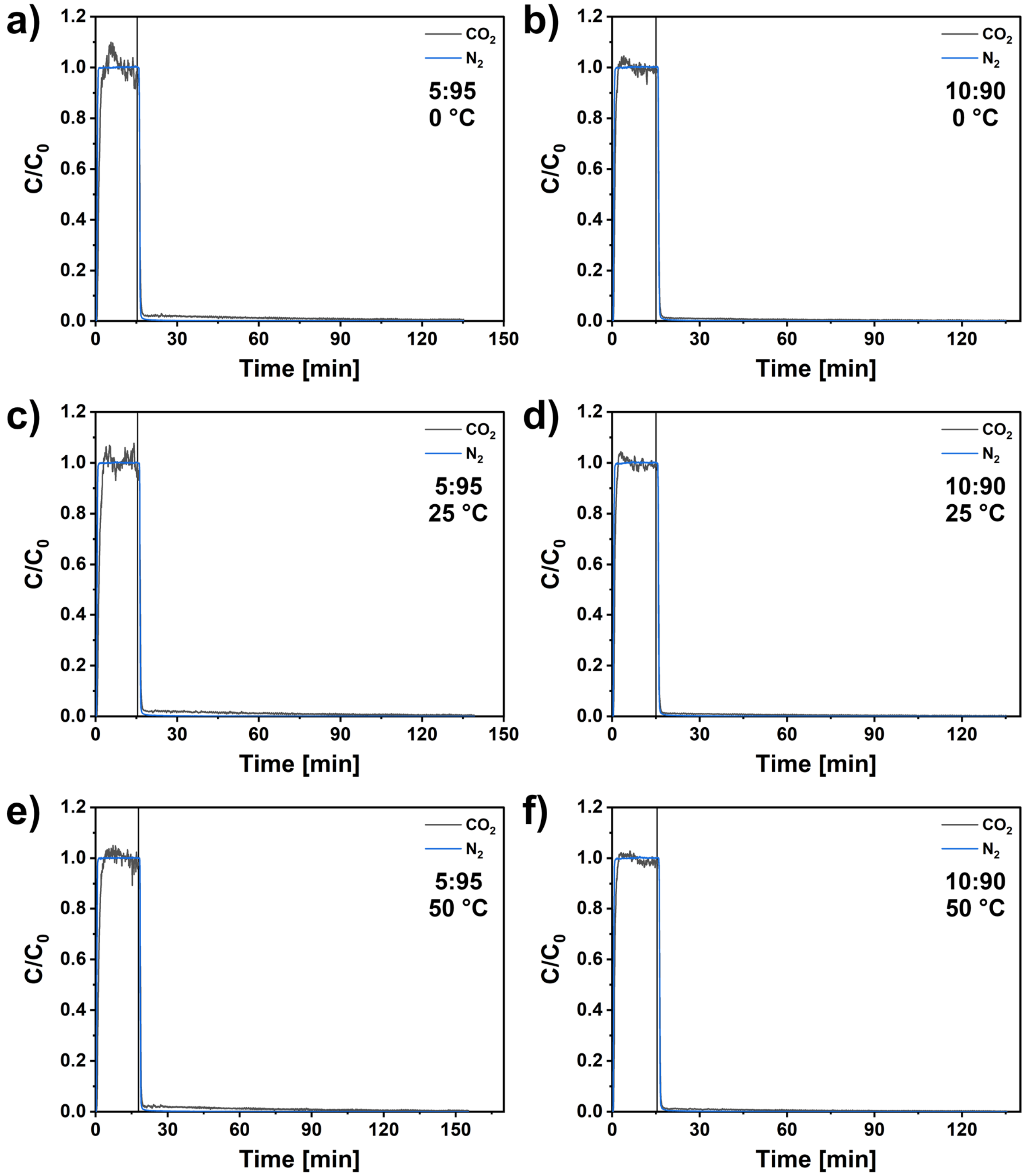

Figure S2-25: CO_2 and N_2 breakthrough curves of a gas mixture with 5:95 (a, c, e) and 10:90 (b, d, f) CO_2:N_2 at 0 °C (a, b), 25 °C (c, d) and 50 °C (e, f) measured with glass beads as filling material. The measurements were performed at 5 bar overall pressure and with a flowrate of 100 mL/min. For a gas composition CO_2:N_2 of 5:95, 2.5% CO_2 and 47.5% N_2 (2.5 bar adsorptive pressure) in He were used and for a gas composition CO_2:N_2 of 10:90, 5% CO_2 and 45% N_2 (2.5 bar adsorptive pressure) in He were used.
